# Uses of Virtual Care in Primary Care: Scoping Review

**DOI:** 10.2196/55007

**Published:** 2025-02-14

**Authors:** Payal Agarwal, Glenn George Fletcher, Karishini Ramamoorthi, Xiaomei Yao, Onil Bhattacharyya

**Affiliations:** 1 Institute for Health System Solutions and Virtual Care Women’s College Hospital Toronto, ON Canada; 2 Department of Family and Community Medicine University of Toronto Toronto, ON Canada; 3 Program in Evidence-Based Care Department of Oncology McMaster University Hamilton, ON Canada; 4 Department of Health Research Methods, Evidence, and Impact McMaster University Hamilton, ON Canada

**Keywords:** primary care, telemedicine, telehealth, virtual care, virtual health, virtual medicine, remote consultation, telephone consultation, video consultations, medical informatics, digital health, digital technology, digital intervention, COVID-19, SARS-CoV-2, coronavirus infections, PRISMA

## Abstract

**Background:**

The COVID-19 pandemic catalyzed an uptake in virtual care. However, the rapid shift left unanswered questions about the impact of virtual care on the quality of primary care and its appropriateness and effectiveness. Moving forward, health care providers require guidance on how best to use virtual care to support high-quality primary care.

**Objective:**

This study aims to identify and summarize clinical studies and systematic reviews comparing virtual care and in-person care in primary care, with a focus on how virtual care can support key clinical functions such as triage, medical assessment and treatment, counseling, and rehabilitation in addition to the management of particular conditions.

**Methods:**

We conducted a scoping review following an established framework. Comprehensive searches were performed across the following databases: Embase, MEDLINE, PsycInfo, Emcare, and Cochrane Database of Systematic Reviews. Other well-known websites were also searched. PRISMA-ScR (Preferred Reporting Items for Systematic Reviews and Meta-Analyses extension for Scoping Reviews) guidelines were followed. Articles were selected by considering article type, language, care provided, intervention, mode of care delivery, and sample size.

**Results:**

A total of 13,667 articles were screened, and 219 (1.6%) articles representing 170 studies were included in the review. Of the 170 studies included, 142 (83.5%) were primary studies, and 28 (16.5%) were systematic reviews. The studies were grouped by functions of primary care, including triage (16/170, 9.4%), medical assessment and treatment of particular conditions (63/170, 37.1%), rehabilitation (17/170, 10%), and counseling (74/170, 43.5%). The studies suggested that many primary care functions could appropriately be conducted virtually. Virtual rehabilitation was comparable to in-person care and virtual counseling was found to be equally effective as in-person counseling in several contexts. Some of the studies indicated that many general primary care issues could be resolved virtually without the need for any additional follow-up, but data on diagnostic accuracy were limited. Virtual triage is clinically appropriate and led to fewer in-person visits, but overall impact on efficiency was unclear. Many studies found that virtual care was more convenient for many patients and provided care equivalent to in-person care for a range of conditions. Studies comparing appropriate antibiotic prescription between virtual and in-person care found variable impact by clinical condition. Studies on virtual chronic disease management observed variability in impact on overall disease control and clinical outcomes.

**Conclusions:**

Virtual care can be safe and appropriate for triage and seems equivalent to in-person care for counseling and some rehabilitation services; however, further studies are needed to determine specific contexts or medical conditions where virtual care is appropriate for diagnosis, management outcomes, and other functions of primary care. Virtual care needs to be adapted to fit a new set of patient and provider workflows to demonstrate positive impacts on experience, outcomes, and costs of care.

## Introduction

### Background

The COVID-19 pandemic led to a significant uptake of virtual care use, with increasing recognition that virtual care will continue to play a key role in Canada’s health care system [1]. Before the onset of the pandemic, 1.2% of all primary care visits in Canada occurred virtually [2]. By contrast, between January 2021 and March 2022, there was a significant uptake of virtual care use in primary care, with 38% of all family physician appointments in Canada being conducted virtually [3]. Similar trends are also observed globally. In the United States, the proportion of primary care physicians using virtual care increased from 5% before the pandemic to 46% during the pandemic [4,5]. In addition, economic evaluations of virtual care suggest that virtual care may be more cost-effective in some circumstances, reducing the cost per episode of care and the cost to attend the appointment (eg, travel and parking costs) compared to in-person care [6]. These cost savings and conveniences for patients contribute to their positive perceptions about using virtual care. By contrast, some studies have found that neither patients nor providers perceive the quality of virtual visits to be better than that of in-person visits [7-10]. In particular, the rapid shift in care delivery without care redesign has raised concerns about appropriateness, effectiveness, and equity [3]. Furthermore, as health systems return to prepandemic visit volumes, there are concerns that these new care modalities could increase workload, leading to increased burnout and eventually reduced staff resource capacity [11].

A broad range of stakeholders have suggested that patients should be the focal point of decision-making and that virtual care should be built into streamlined workflows that cover all aspects of primary care, with meaningful incentives to drive system impact [12]. However, there is little empirical evidence to support putting these principles into practice. Reviews to date looking at outcomes related to virtual primary care have shown mixed or uncertain outcomes, with minimal guidance to drive care decisions [13]. Nevertheless, strong continued interest from patients suggests that virtual care can provide benefits in supporting quality primary care [13]. Studies have suggested that high-quality, sustainable models for primary virtual care require mechanisms to support triage and shared decision-making on the use of different modalities in clinical practice [12,14]. Consensus guidelines have also suggested that the use of virtual care in primary care should be based on multiple factors, including clinical appropriateness, patient preferences, and equity [15,16]. Primary care involves many functions, such as triaging incoming requests, diagnosing acute conditions, managing chronic conditions, providing counseling, and supporting rehabilitation (among others). Despite the ongoing use of virtual care by many providers, questions remain about which primary care functions are best suited to this mode of care.

This paper is based on an evidence summary developed by the Program in Evidence-Based Care (PEBC) at McMaster University at the request of the Population Health and Value-Based Health Systems portfolio of Ontario Health (a government agency in Canada) as one of the inputs into a guidance document, “Clinically Appropriate Use of Virtual Care in Primary Care” [16]. This paper focuses on a subset of the results of this broader review to explore various primary care use cases for virtual care.

### Objectives

The main objective of this review was to identify and summarize clinical studies of virtual care use in primary care. Of particular interest were studies indicating the circumstances when synchronous virtual care (primarily videoconference or telephone interactions) was likely to be equivalent, superior, or inferior to in-person care to inform decisions about appropriate modes of interaction with patients. Rather than make definitive statements on the benefits or harms of virtual care or the value of virtual-only primary care, we have organized the findings in ways that highlight potential benefits across a range of primary care functions to indicate which functions of primary care may be most suitable for virtual care. We have also suggested ways of conducting and reviewing future studies.

## Methods

### Literature Search and Screening

The literature search strategy and screening were performed according to systematic review methodology. The review protocol was developed by the coauthors and approved at the PEBC and by the sponsor (Population Health and Value-Based Health Systems, Ontario Health) before the commencement of the review; a post hoc decision was made to analyze and present the results as a scoping review rather than as a systematic review. A scoping review includes a predefined protocol and systematic approach to literature searches, but instead of focusing on the rigor of included studies, it reviews the literature to identify or map the state of knowledge and gaps and the research conducted, as well as identify further research needs [17-19]. This scoping review follows the guidelines formulated by Arksey and O’Malley [17] and aligns with the PRISMA-ScR (Preferred Reporting Items for Systematic Reviews and Meta-Analyses extension for Scoping Reviews) statement [20]. The PRISMA checklist was also used to support reporting (refer to Multimedia Appendix 1) Research team members, using an inductive process, grouped the results into different categories, representing distinct use cases in primary care. While a formal assessment of the strength or quality of evidence was not conducted for each study—an aspect that differentiates a scoping review from a systematic review—characteristics such as study design (eg, randomized controlled trial [RCT] and prospective or retrospective comparative study), sample size, and type of comparison were extracted. In interpreting and discussing the results, we considered factors such as study size, trial design, consistency of results, and whether the studies addressed very narrow, specific issues or broader topics.

### Definitions

The following subsections provide definitions and explanations of the key terms used throughout this paper.

#### Virtual Care

The term *virtual care* was defined as synchronous remote interaction between patients and clinicians to replace all or a portion of face-to-face (in-person) interactions. Care is considered synchronous when the patient and clinician are both present at the same time, and there is 2-way communication without delays in response (ie, in real time). It can include both video and telephone interactions. By contrast, asynchronous communication does not take place in real time. Asynchronous communication was included only if it was used as part of synchronous communication.

#### Primary Care

*Primary care* was defined as “the first point of contact between a patient and the health care system and includes illness prevention, health promotion, diagnosis, treatment, and rehabilitation and counseling” [21]. It included care by family or general practice physicians and nurse practitioners, general practice pediatricians and geriatricians, midwives, psychologists, psychotherapists, social workers, pharmacists, and physiotherapists. Care provided by dentists, psychiatrists, or medical specialists normally seen only by referral or in a hospital setting was excluded. The definition encompassed a variety of health care providers to reflect the broadest range of staff typically found in a multidisciplinary primary care team.

#### Triage

*Triage* refers to the initial decision process within primary care practice upon first patient contact, resulting in the allocation of patients to either in-person or virtual family practice appointments or, in some cases, to administrative assistance or other types of care. Articles categorized under triage included those that discuss signposting—a strategy designed to direct patients to the right provider at the right time and the right place at the first point of contact with primary care [22]. As the first point of contact for many patients, triage is often considered a core function of primary care.

### Research Question

To explore the effectiveness of virtual care in primary care settings, this study addresses the following research question:

When using virtual modalities to seek or deliver primary care, are there differences in the outcomes of interest (either in general or for specific medical conditions or purposes of appointments) between

- synchronous virtual care by telephone or video compared to exclusive in-person care;
- synchronous virtual care by telephone or video plus asynchronous care (SMS text or secure messaging and email) compared to exclusive in-person care?

### Outcomes of Interest

Outcomes of interest from a patient perspective included equity and accessibility (race, ethnicity, socioeconomic status, urban or rural or remote residence, age, gender, computer literacy, and mobility), availability of appointments (time, location, and wait time to get an appointment), and on-time appointments. Outcomes relevant to multiple stakeholders—patients, care providers, and health systems—included disease stability, improvement or deterioration, complications, or death; satisfactory resolution of the reason for visit; subsequent in-person visit to primary care practitioner because virtual care was not appropriate for assessment or treatment; referral to specialists, emergency department use, or hospital admissions; rates of blood tests, clinical laboratory tests, and diagnostic imaging; prescription of antibiotics or other medications; overall health of patients of primary practice team; and continuity of care.

### Search Strategy

Embase, MEDLINE, PsycInfo, Emcare, and Cochrane Database of Systematic Reviews databases using the Ovid platform were searched from 2014 to November 24, 2021, using terms related to the concepts of *virtual care* and *primary care* (refer to Multimedia Appendix 2 [6,23-240] for the full search strategy). Due to time constraints for the project, a pragmatic decision was made to begin the database search in 2014. Therefore, publications before 2014 were not screened and only included in the literature review if cited elsewhere. Websites that the PEBC routinely consults to identify guidelines and reviews were also searched from 2015 to March 2022 (Multimedia Appendix 2); we did not search the websites of health care organizations devoted to primary care or specific diseases. Articles cited in other publications were included, regardless of publication year, if they met the other inclusion criteria.

### Inclusion and Exclusion Criteria and Screening of Primary Studies

The inclusion and exclusion criteria are outlined in Textbox 1. Articles were screened based on these criteria, which included categories such as article type, language, care provided, intervention, mode of delivery, and sample size.

A post hoc decision was made to include noncomparative studies that did not initially meet the inclusion criteria, but only if they were categorized as relating to triage. This adjustment was made because triage is a core component of first-contact care provided by primary care practitioners.

A review of the titles and abstracts of the primary literature, followed by data extraction for studies meeting the specified criteria, was conducted by GGF, a professional health research methodologist at the PEBC at McMaster University. The coauthors were consulted in cases of uncertainty. An independent audit of the extracted data was conducted by Jilian Sing (refer to the Acknowledgments section). Discrepancies were noted and addressed.

**Inclusion and exclusion criteria.**

Inclusion criteria

- Study type: randomized controlled trial; other comparative studies (virtual vs in-person care)
- Language: abstract or full text in English
- Care provided: primary care study (refer to the definition of *primary care* in the Definitions subsection); care provided in a continuous primary care practice or a clinic that offered both walk-in and virtual care
- Intervention: virtual care provided by the same clinician or primary care team responsible for in-person care
- Mode of care delivery: synchronous virtual care (telephone or video), which replaced in-person care, including replacing of a subset of in-person appointments
- Sample size: sample size ≥30 (this is often considered sufficient for the central limit theorem to hold true, enabling the detection of differences between 2 study groups) [241]

Exclusion criteria

- Article type: trial registries or other study or review protocols without published results; editorials or commentaries; noncomparative surveys or questionnaires about patient or clinician experience; case studies; case series without a comparison group; conference abstracts
- Language: not English
- Care provided: study investigated the role of education, materials, lifestyle adaptation, exercise, diet, and so on, and the main study comparison was not virtual versus in-person delivery of these interventions; replacement of in-person care provided by 1 professional or treatment team or unit with virtual or in-person care provided by a different professional or team
- Intervention: study related to virtual-only practices (eg, “walk-in” telephone clinics or hotlines); app or remote patient monitoring;
- Mode of care delivery: SMS Text, email, or other asynchronous interventions, unless as a supplement to synchronous virtual care components
- Sample size: sample size <30

### Systematic Reviews

Publications described as being systematic reviews or meta-analyses by their authors (primarily in the title or abstract) were included if they reported on primary studies meeting the inclusion criteria (Textbox 1). Reviews were also considered to be systematic reviews if the authors reported the databases searched, search strategy, and inclusion and exclusion criteria; the results section included a list of the reviewed studies along with extracted data; and the review was not otherwise classified by the authors. Systematic reviews on virtual care that passed initial screening but predominantly included studies not meeting the inclusion criteria were excluded; these reviews were used only to identify additional primary studies not found in the database search. Only reviews published in English were considered, with no restrictions placed on the publication date of individual studies.

## Results

### Overview

**Figure 1:**
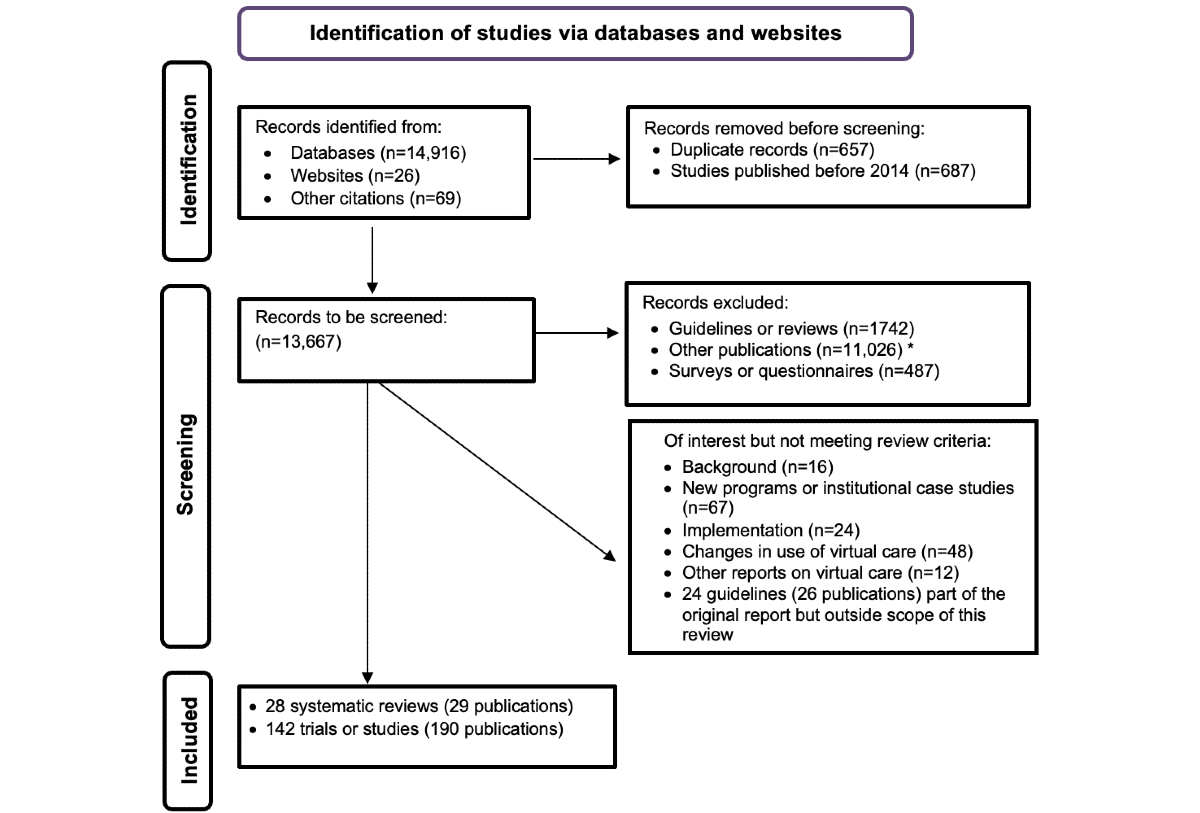
PRISMA (Preferred Reporting Items for Systematic Reviews and Meta-Analyses) diagram showing the number of studies identified, screened, assessed for eligibility, and included in the final analysis. *Studies were excluded for the following reasons: did not meet our definition of primary care or virtual care (3134/11,026, 28.42%); an additional intervention was being studied (2076/11,026, 18.83%); not full publications of completed studies (protocols, trial registrations, or conference abstracts; 2060/11,026, 18.68%); no comparison group for the outcomes of interest (1318/11,026, 11.95%); focused on the education of clinicians or interprofessional consultation (864/11,026, 7.84%); asynchronous interventions or not involving patient-clinician contact (821/11,026, 7.44%); case studies or <30 patients per group (362/11,026, 3.28%); letters, editorials, or commentary (195/11,026, 1.77%); virtual reality (142/11,026, 1.29%); and other (54/11,026, 0.49%).

The database search identified 14,916 citations. In addition, 26 studies were identified through website searches, and 69 studies were found from the reference lists of other publications (Figure 1). The included studies were categorized into primary care functions, including triaging of incoming requests, diagnosis of a range of conditions through general primary care, rehabilitation, and counseling. A breakdown of the 170 included studies is presented in Table 1. Data for each study have been extracted and are reported in the tables in Multimedia Appendix 2. Primary studies on triage (24 publications representing 14 studies) are summarized in Table S1 in Multimedia Appendix 2 [23-46], and systematic reviews on triage are summarized in Table S2 in Multimedia Appendix 2 [47,48]. As the number of studies addressing specific types of virtual care was very limited, we decided not to subdivide results further by this category.

For studies on virtual care other than counseling, there were 64 publications representing 62 primary studies and 19 publications representing 18 systematic reviews (Tables S3-S13 in Multimedia Appendix 2 [6,49-130]). These have been subdivided by the categories listed in Table 1 and consisted of 11 RCTs and 18 possibly prospective nonrandomized trials; the rest were retrospective studies that generally used chart review or registry data. Only a few of the studies used multivariate analysis to control for possibly confounding factors. Table S3 [49-68] and Table S4 [6,69-73] in Multimedia Appendix 2 summarize studies and systematic reviews, respectively, in general primary care. Table S5 in Multimedia Appendix 2 [74-82] summarizes studies conducted during COVID-19–related restrictions; Table S6 in Multimedia Appendix 2 [83-90] and Table S7 in Multimedia Appendix 2 [91,92] summarize studies and systematic reviews of minor infections, respectively; and Table S8 in Multimedia Appendix 2 [93-96] summarizes studies of COVID-19 diagnosis and management. Table S9 in Multimedia Appendix 2 [97-106] and Table S10 in Multimedia Appendix 2 [107,108] summarize studies and reviews of virtual care in chronic disease management, respectively, while Table S11 in Multimedia Appendix 2 [109-113] reports on the use of virtual care in medical abortion. Clinical studies and systematic reviews in rehabilitation are covered in Table S12 in Multimedia Appendix 1 [114-121] and Table S13 in Multimedia Appendix 2 [107,122-130], respectively.

Primary counseling studies (102 publications representing 66 studies) are summarized in Table S14 in Multimedia Appendix 2 [131-232], while systematic reviews on counseling (n=8) are reported in Table S15 in Multimedia Appendix 2 [233-240]. The number of studies on counseling was slightly more than the number of other studies combined. Most of the counseling studies (50/65, 77%) were RCTs, providing stronger and higher-quality evidence on this topic.

**Table 1:** Breakdown of included studies by primary care function.

Reason for consultation		Studies, n (%)		
		Primary studies (n=142)	Systematic reviews (n=28)	Total (n=170)
**Triage**		14 (9.9)	2 (7.1)	16 (9.4)
**Medical assessment and treatment**		54 (38)	9 (32.1)	63 (37.1)
	General primary care	19 (35.2)	5 (55.6)	24 (38.1)
	Primary care during COVID-19–related restrictions	9 (16.7)	0 (0)	9 (14.3)
	Minor infections	8 (14.8)	2 (22.2)	10 (15.9)
	COVID-19 management	4 (7.4)	0 (0)	4 (6.3)
	Chronic disease management	9 (16.7)	2 (22.2)	11 (17.5)
	Medical abortion	5 (3.2)	0 (0)	5 (7.9)
**Rehabilitation**		8 (5.6)	9 (32.1)	17 (10)
**Counseling**		66 (46.5)	8 (28.6)	74 (43.5)

### Triage

#### Overview

The 14 primary studies involving triage (Table S1 in Multimedia Appendix 2 [23-46]) used various designs and often did not allow a direct comparison of virtual versus in-person care. Of these 14 studies, 10 (71%) were conducted in the United Kingdom, while 1 (7%) study each was conducted in Canada, Israel, and the Netherlands, 2 studies were conducted in Denmark. The 2 systematic reviews—1 (50%) on telephone triage [47] and 1 (50%) on remote triage [48]—are summarized in Table S2 in Multimedia Appendix 1.

#### Telephone Triage

The most common practice involved telephone triage by a nurse or other office staff and the assignment of patients to telephone consultation if appropriate and if the patient did not object. Both patient and disease characteristics determined the modality of care. Of the 16 studies on triage, only 2 (13%) were of this design [42,44]; most reports of this type were excluded because there was generally no comparison group. A variation used by 3 (19%) of the 16 studies was a telephone-first approach, where a general practitioner (GP) spoke by telephone with all patients (except those with administrative queries) and either addressed the issue during the call or scheduled an in-person visit [24-29,39]. Of the 16 studies, 2 (13%) involved triage by GPs in Denmark [43,46]. Of these 2 studies, 1 (50%) found that approximately 40% of the calls resulted in face-to-face consultation [43], while 1 (50%) reported medication prescription rates [46]. In 2 (13%) of the 16 studies, patients accessed the medical office using a web-based form, and patients with nonadministrative concerns were allocated to a GP who decided on the mode of contact [30,31,45].

The only RCT was the ESTEEM trial, and 7 (44%) of the 16 publications on triage covered various aspects of this trial [32-38]. Conducted in England, this trial, which used a combination of the aforementioned approaches, may serve as the most significant source of insights into triage. Patients (n=15,394) requesting same-day appointments were randomized to triage by a nurse, triage by a GP, or usual care. Estimated overall contact durations (triage+subsequent contacts on the same day) were 10.3 minutes for GP triage, 14.8 minutes for nurse triage, and 9.6 minutes for usual care. The number of deaths, emergency hospital admissions, and accident and emergency department attendances were not different [36]. Triage by a nurse practitioner resulted in fewer in-person visits than triage by practice nurses (odds ratio 0.19, 95% CI 0.07-0.49); nurse practitioners were more likely to definitively manage patients within the triage consultation [33].

#### Signposting

Another study from the United Kingdom [41] used signposting in 2 practices to reduce GP telephone workload; only patients for whom a physician visit was judged essential were given a telephone appointment. The remaining callers were referred to allied health professionals, alternative services, nonmedical staff, or self-help [41]. Approximately 40% to 60% of telephone appointments were eliminated. The proportion of essential consultations taking place at the 2 practices increased from 28.6% and 27.3% at baseline to 82.6% and 71.4%, respectively.

#### Emergency Assessment

A total of 4 (25%) out of 16 studies on triaging explored the use of virtual care to assist in triaging and managing emergency calls within health systems. Of the 16 studies on triaging, 1 (6%) looked at adding GP support to emergency calls, either in person or by telephone [40]. The study found that 8.1% of all calls to emergency services could be triaged to ambulance personnel plus GP support. Of these patients, 78% were not transported to hospital, although a transfer was more often avoided if the patient was assessed in person by the GP. The remaining study, which was conducted in British Columbia reported experience using HealthLink BC Emergency iDoctor-in-assistance [23]. It looked at patients who called an 811 nurse care navigation service. Callers who were triaged to seek care within 24 hours were referred to a physician by videoconferencing. Of the callers directed to videoconferencing with HealthLink BC Emergency iDoctor-in-assistance physicians, 33.8% were advised to attempt home treatment, 38.3% to contact a primary care physician within 1 week, 15% to attend an emergency department immediately, and 7.1% to contact their primary health care provider right away.

### Medical Assessment and Treatment

#### General Primary Care

A total of 24 (38%) of the 63 studies on medical assessment and treatment were on general primary care. These studies in general primary care are summarized in Table S3 in Multimedia Appendix 2 [49-68] and systematic reviews in Table S4 in Multimedia Appendix 2 [6,69-73]. Of the 19 primary studies classified as general primary care, 15 (79%) involved general practice contacts for all or several reasons [49-58,60-65], while 1 (5%) reviewed those with acute deterioration within 3 days of a primary care visit [59] and found a slightly higher rate of self-referral to emergency services after telephone consultations compared to in-person visits.

Of the 15 studies involving general practice contacts for all or several reasons, 1 (7%) focused on the 11 most common illnesses managed via telehealth: sinusitis, upper respiratory infection, urinary tract infection, conjunctivitis, bronchitis, pharyngitis, influenza, cough, dermatitis, digestive symptoms (nausea, vomiting, or diarrhea), and ear pain [65]. This large retrospective study used insurance company claims and focused on the treatment of acute non-urgent conditions. It found that follow-up rates for both virtual and in-person care were similar, suggesting comparable clinical resolution of these issues through virtual modalities, although antibiotic use was higher and laboratory testing lower for the virtual care group [65].

Of the 2 RCTs that looked at a broad range of virtual primary care issues, 1 (50%) crossover study involved evaluations of patients by 2 physicians [49,50]: patients were randomized to either 1 virtual and 1 in-person assessment the same day or 2 in-person assessments. Follow-up for chronic diseases such as hypertension, elevated cholesterol level, and diabetes and consultations for acute diseases such as upper respiratory illness or sinusitis and musculoskeletal complaints were the most common. The diagnostic agreements between the physicians were 84% between face-to-face and virtual visits and 80% between the 2 face-to-face visits. This study was among the few that directly compared the reliability of diagnosis for common conditions via in-person or virtual care.

The other RCT randomized patients to either a face-to-face appointment on that day or a callback that morning by a physician who either offered advice or arranged an in-person visit [51]. Telephone consultations were shorter (6.7 vs 8.2 min; *P*=.002) but resulted in slightly more follow-up consultations within 2 weeks (0.6 vs 0.4 consultations; *P*=.01), thus offsetting the time savings. Blood pressure was more frequently measured in person (13.3% vs 6.6%), but there were no significant differences in patient perceptions or other secondary outcomes.

Of the 24 studies on general primary care, 1 (4%) [67] compared video to telephone communication in emergency paramedicine response and found that video enhanced clinical evaluation 85% of the time; the odds ratio for emergency transport was 0.80 (95% CI 0.62-1.03).

Of the 9 systematic reviews on medical assessment and treatment, 5 (56%) were on virtual care in general without specifying any diseases or conditions [6,69-73]. The reviews by Totten et al [71-73] for the US Agency for Healthcare Research and Quality [71-73] concluded that telehealth consultations are effective in providing services or improving outcomes, although evidence is stronger for some applications. The reviews found evidence of effectiveness for counseling and the management of chronic conditions. However, overall impact, including cost-effectiveness, varied significantly based on how virtual care was integrated into the larger health service delivery ecosystem.

#### Primary Care During COVID-19–Related Restrictions

Of the 63 studies on medical assessment and treatment, 9 (14%) reported the use of virtual care due to COVID-19–related restrictions and are summarized in Table S5 in Multimedia Appendix 1 [74-82]. Of these 9 studies, 1 (11%) found that there was a rapid increase in the use of virtual care—from 31% in April 2019 to 90% in April 2020. Of the visits conducted virtually, 89% were by telephone and 1% by video [80]. Another study found that 71% of the video visits included visual observation–dependent findings that could not be assessed using a telephone; however, common barriers to using video visits were the lack of appropriate equipment and patient preferences [74]. Among the studies looking at primary care during the COVID-19 pandemic, there were variable results on the impact of age on the use of video visits: the study by Eberly et al [75] found that older individuals were less likely to use video visits, whereas Schenker et al [76] found that age was a significant positive predictor of having video visits.

#### Minor Infections

Of the 63 studies on medical assessment and treatment, 10 (16%) discussed antibiotic prescription (primary studies: n=8, 80%; Table S6 in Multimedia Appendix 1 [83-90]; systematic reviews: n=2, 20%; Table S7 in Multimedia Appendix 1 [91,92]). The 2 reviews did not have sufficient evidence to draw strong conclusions about the impact of virtual care on antibiotic prescribing but noted variable effects by condition, with higher rates for some conditions and lower rates for others. Of the 8 primary studies, 5 (62%) retrospective studies evaluated antibiotic prescribing for acute respiratory infections [83,85-88], while 1 (12%) evaluated antibiotic prescribing for urinary symptoms or infections [84]; in addition, 4 (50%) studies found similar or lower antibiotic prescribing rates with virtual compared to in-person care, while 1 (12%) reported higher antibiotic prescribing rates for telemedicine than in-person visits. This varied by condition within the same health care organization, with a chart review finding lower rates of antibiotic prescribing for sinusitis by SMS text and telephone than face-to-face visits and similar rates for urinary tract infections across all 3 modes [83,84]. By contrast, a review of claims data with matched populations showed higher rates of prescribing for acute respiratory infections for direct-to-consumer telemedicine versus urgent care or care provided by the patient’s usual primary care provider [86]. A similar pattern was found in a study comparing retail health clinics with urgent care and primary care providers [65].

Some of the other general studies [55,63,65] also noted antibiotic prescribing as one of the outcomes. 1 of the 10 studies on minor infections (10%) compared the outcomes of patient-selected modality with the same provider, finding higher rates of antibiotic prescribing for in-person visits (10.6% for video vs 9.7% for telephone vs 13.5% for in-person visits) [55]. Of the 10 studies, 2 (20%) evaluated and found higher antibiotic prescribing rates with telephone consultations for patients with conjunctivitis, a condition that usually requires visual assessment [89,90].

#### COVID-19 Management

Of the 4 studies that reported on the assessment of patients with symptoms consistent with COVID-19 infection (Table S8 in Multimedia Appendix 2 [93-96]), 3 (75%) compared remote assessment (by telephone, video, or unspecified) with in-person care. Some studies conducted during the COVID-19 pandemic used antibiotic prescribing as one of the outcomes. Of these 4 studies, 1 (25%) [94] found that in-person visits involved more testing for influenza and higher antibiotic prescribing rates.

#### Chronic Disease Management

Of the 63 studies focused on medical assessment and treatment, 11 (18%) focused on chronic disease management (primary studies: n=9, 82%; Table S9 in Multimedia Appendix 1 [97-106]; systematic reviews: n=2, 18%; Table S10 in Multimedia Appendix 1 [107,108]). Of the 9 primary studies, 3 (33%) RCTs were conducted in patients with asthma [97-99], and all found higher rates of follow-up appointments in the telephone groups; 2 (22%) retrospective studies found similar diabetes control using either telephone [100] or videoconferencing [101] compared to face-to-face or usual care; and, by contrast, 1 (11%) retrospective study examining the use of telehealth by community health care workers to support Hispanic patients with low-income status with uncontrolled diabetes (defined by glycated hemoglobin level ≥9%) reported that patients were much less likely to achieve glycemic control with telephone visits alone compared to either exclusively in-person visits or a mix of in-person and virtual visits [102]. However, 1 in-person visit plus ≥1 telephone visit was worse (longer time to diabetes control) than 1 in-person visit alone [102], suggesting that adjustment for the confounders was not adequate.

#### Medical Abortion

Of the 63 studies on medical assessment and treatment, 5 (8%) explored the use of virtual care in medical abortion assessment or follow-up (Table S11 in Multimedia Appendix 1 [109-113]). These studies found no difference in successful abortion rates or adverse events, reported similar satisfaction, and suggested that providing care by telephone improved access compared to in-person care.

### Rehabilitation

Rehabilitation studies (17/63, 27%) included those focusing on physiotherapy and cardiac rehabilitation. Of the 17 studies, 8 (47%) were primary studies (Table S12 in Multimedia Appendix 2 [114-121]), and 9 (53%) were systematic reviews (Table S13 in Multimedia Appendix 2 [107,122-130]). Specifically, of the 8 primary studies, 2 (25%) involved cardiac rehabilitation. The noninferiority RCT compared remotely monitored telerehabilitation to center-based programs and found similar benefits remote noninferior in terms of improvement in maximal oxygen consumption and health state [114]. The quasi-experimental study compared home-based videoconference rehabilitation to in-hospital rehabilitation; there was similar improvement in the 6-minute walk test, and the authors concluded that video rehabilitation was feasible [115].

Of the 8 primary studies, 2 RCTs (25%) [116,117] and 1 (12%) retrospective study [118] found that telerehabilitation after total knee or total hip and knee arthroplasty was noninferior or comparable to traditional therapy. Studies on movement assessment in children [119] and physiotherapy assessment [121] found 92% and 83% agreement between video and in-person assessment, respectively; it is unclear whether this is sufficient. A study on physical activity coaching [120] found no significant differences between control (no intervention), in-person, and telephone intervention.

### Counseling Studies

Of the 8 systematic reviews on counseling (Table S15 in Multimedia Appendix 2 [233-240]), 3 (37%) focused on psychotherapy [233,234,236]; 1 (12%) explored interventions targeting smoking, nutrition, alcohol consumption, physical activity, and obesity [235]; 1 (12%) each focused on depression [237], posttraumatic stress disorder [238], and suicide [239]; and 1 (12%) investigated traumatic stress during a pandemic [240]. Overall, these reviews concluded that virtual care is an established medium for delivering counseling, with evidence suggesting equivalence to in-person care in many areas of counseling.

Most of the counseling studies (50/74, 68%; Table S14 in Multimedia Appendix 2 [131-232]) were RCTs. Of the 50 RCTs, 19 (38%) were designed as noninferiority studies. Many studies compared counseling by either telephone or videoconferencing to the same counseling provided in person (face-to-face). Of the 74 counseling studies, 3 (4%) [177,191-193] used real-time SMS text or chat conversations (all other studies involving SMS text-based communication were asynchronous and had been excluded). Cognitive behavioral therapy (CBT) was used in 18 (24%) of the 74 studies for treating depression, anxiety, pain, insomnia, eating disorders such as bulimia nervosa, and obsessive-compulsive disorder. Components of CBT may be present in some other studies that were described as behavioral studies, although they were not explicitly labeled as CBT. The overall evidence indicates that outcomes for telephone and video CBT are similar or noninferior to those for face-to-face CBT, although some differences for specific populations were noted. Other commonly used counseling types were problem-solving therapy, behavioral activation therapy, behavioral treatment interventions, prolonged exposure therapy, and cognitive processing therapy, with the latter 2 names used especially for posttraumatic stress disorder. For lifestyle-type interventions for weight loss, smoking cessation, and alcohol use, the type of counseling was less likely to fit within the traditional psychotherapy categories. While evidence for CBT is the greatest, the overall evidence suggests a role for telephone or video counseling in many other areas.

## Discussion

### Principal Findings

This scoping review synthesized studies that compared in-person and virtual care in primary care, with a mix of studies conducted before and during the COVID-19 pandemic. The studies were categorized into primary care functions, including triaging of incoming patient requests, medical assessment and treatment (including general primary care, minor infections, COVID-19 management, chronic disease management, and medical abortion), rehabilitation, and counseling. The review showed strong evidence that virtual care is equivalent to in-person care for counseling and comparable for some types of rehabilitation. The results suggest that virtual triaging is clinically appropriate and may lead to fewer in-person visits, especially when conducted by a physician or a nurse practitioner. However, for other primary care functions, outcomes from the included studies varied. Several studies demonstrated that many general primary care issues could be resolved virtually without the need for in-person follow-up, but only a few looked at diagnostic accuracy. A study that focused on diagnostic agreement between modalities for common primary care conditions found strong alignment [49,50]. Studies on chronic disease management observed variability in their benefit in using virtual care. Studies comparing appropriate antibiotic prescription between virtual and in-person care found variable impact depending on the clinical condition, whether patients chose a modality or were randomized to one, and the clinical context. A few of the studies measured the impact on emergency department visits or hospitalizations and found no difference, but they did not adjust for the type or severity of the illness [53,55]. Overall, the variation in impact may be a result of the relative effectiveness of the modality for a given condition or use case, but in some of the studies the differences may be driven by how the modality is selected, the practice context, and health care provider incentives within a health care organization.

The evidence for the overall experience and value of virtual care is evolving, but our review suggests some advantages for patients and unclear impacts on provider workload and system costs. This aligns with previous reviews that have found uncertain outcomes for virtual primary care as a whole [6,13]. In our review, most patients reported a similar overall experience between virtual and in-person interactions; however, certain benefits of virtual care were noted, including in terms of cost, travel time, convenience, waiting time, and access to more specialized care [39,42,69,70]. Therapeutic alliance was similar between in-person and virtual counseling in the studies reviewed. This contrasts with an earlier systematic review of virtual care in cancer, where some clinicians reported that they found it more difficult to comfort patients during vulnerable times, found virtual care more stressful, and had concerns about therapeutic alliance [242].

The included studies did not assess the impact on continuity of care or health equity, both of which are key components of primary care and patient experience. Other studies that aim to explore continuity of care after a primary care visit only do so by examining primary care as a whole; for example, the study by Reed et al [243] found a slightly higher percentage of in-person return visits and emergency department visits after video or telephone visits compared to in-person primary care visits, but the authors did not examine the differences based on the type of primary care function. Given that continuity of care is a defining characteristic of primary care and has been shown to be instrumental in driving primary care’s positive impact on equity and quality of care, future studies should explore the impact of virtual care use on different primary care functions in the context of its impact on continuity of care [244,245]. In addition, the impact of virtual care on provider workload and system costs was less clear in our review. Telephone visits tended to be shorter than in-person interactions, but it was unclear how the addition of virtual care affected overall workloads. An initial telephone-based clinical triage by nonphysicians resulted in a higher proportion of visits being deemed essential by primary care physicians, suggesting that triage reduced low-value appointments with a physician [48]. Having said that, although the overall workload was not addressed, other studies in the literature highlight the value of virtual triage to physicians, notably that it can improve a clinician’s experience by streamlining patient-clinician communication and could help clinicians better manage their time by reducing their administrative workload [246].

To our knowledge, this is one of the first comprehensive reviews to examine the appropriateness of virtual care in terms of key functions of primary care. Studies examining virtual care use in primary care tend to examine primary care as a whole; for example, the study by Reed et al [243] found that 50% of primary care visits used telemedicine (19% by video and 31% by telephone); however, the impact on these numbers based on primary care function was not studied. Similarly, previous reviews have also focused on examining virtual care use more broadly through categories such as cost and health care use. However, given the shift in practice since the COVID-19 pandemic, it is now more important to understand how and when in the clinical process virtual care should be used [6,13]. In addition, these reviews only included publications from 2020 onward and did not include RCTs, which are a focus of our review. Our review shows that there is significant variability in evidence across different clinical use cases in primary care, making it difficult to provide broad conclusions about the appropriateness of virtual primary care for specific conditions. Instead, our work provides evidence that the impact and outcomes of virtual modalities in primary care should be examined according to the various interaction points along a patient’s journey; for example, there is strong evidence for equivalence between in-person and virtual care for counseling, and thus counseling could be more readily integrated into sustainable, high-quality primary care workflows. By contrast, the use of virtual care for situations that are likely to result in antibiotic prescribing requires further study to understand whether it is indeed equivalent to in-person care. Our review suggests that virtual triage can play a role in supporting primary care workflows, but more research is required to determine the best implementation model. A rapid review by Barnabe et al [247] highlighted some implications for health care organizations to consider before implementing virtual triage. These include ensuring that the platforms are locally based; that they are flexible, responsive, and tailored to regional circumstances; and that implementation is carried out, keeping partnerships and collaborations in mind [247]. These results contrast with those of other reviews on the use of virtual modalities in primary care, which analyzed clinical appropriateness and outcomes across all use cases and were generally unable to make strong recommendations to guide clinical care [15]. Several authors have suggested that a more nuanced approach, one that looks at different uses of primary care, might ultimately provide more insight into driving decisions around patient care and system planning [6,248,249].

Virtual care during the COVID-19 pandemic has expanded to cover a wide range of conditions, and our review suggests that there is still limited evidence to broadly define the optimal mode of interaction (in-person, video, telephone, or asynchronous contact); however, the evidence does provide some guidance. Our review suggests that virtual care is likely at least as good as in-person care for a wide range of primary care issues and should be offered as part of routine practice. Overall, for any given visit, the choice of modality remains a question of clinical judgment and shared decision-making between a patient and their care team. Patient factors, including access to technology, comfort with virtual visits, and maintaining a strong therapeutic relationship, must be considered when deciding the best modality for a given interaction [250]. Virtual care should be embedded in larger service models that allow for easy escalation to in-person follow-up when required. Enabling virtual triage can provide clinics with certain advantages, including improving overall access, better prioritization for the most appropriate modality, and determining whether any information should be provided in advance of the visit. Furthermore, providing counseling, self-management support, education, and ongoing monitoring virtually also offers advantages over regular in-person appointments, including improved convenience and similar outcomes for many patient populations. Virtual care could also be offered to support chronic disease management but may not work well for all patients. Options such as providing smartphones, tablet devices, or computers and limited telephone or internet service were used in some of the studies for individuals with limited technology accessibility [139,152-154,167,195]. However, health organizations and systems would benefit from continued investment in understanding how to better integrate and optimize virtual care to achieve the quintuple aim of improving patient experience, improving population health, improving clinician well-being, reducing health care costs, and advancing health equity [251], using human-centered methods such as co-design, service design, and participatory research or other frameworks [252]. Now that the practice of virtual care is so widespread, ongoing research can help in understanding how it can be leveraged to improve access and quality of care [248].

### Limitations

This review has several limitations. First, it was restricted to studies comparing virtual care to in-person care instead of care (regardless of delivery method) to an objective standard. Therefore, it is applicable only to clinical areas in which there is established in-person care for evaluation and treatment. While many of the included studies were designed to measure the noninferiority of virtual care to in-person care, in-person treatment should not necessarily be the gold standard, especially for counseling studies where effectiveness is often based on a validated tool to measure disease, symptom intensity, or patient-reported outcomes. Second, this is a scoping review rather than a systematic review; therefore, we did not evaluate the methodological quality of the studies or determine which studies might provide more reliable results. In addition, we did not include gray literature in our search to ensure the reliability of the evidence underpinning the recommendations provided in the paper. Having said that, although we supplemented our search using trusted websites, it is possible that the exclusion of gray literature resulted in underrepresented perspectives from policy makers and community organizations that are not captured in academic publications. This could mean that insights from the practical applications of real-world data could have been missed. Furthermore, articles were only screened by a single reviewer, which could have introduced bias in the results if personal biases impacted the interpretation of the literature. Although these limitations were mitigated through an audit conducted by another member of the team, the likelihood of errors is increased when only 1 reviewer is involved. Third, the study contexts varied substantially. Most were cross-sectional studies focused on the initial consultation or ongoing chronic disease management and did not assess the impact on continuity of care, a key component of primary care. Finally, given the time frame for the database search, it is possible that new literature has become available that contributes additional evidence to this topic. Nonetheless, the study demonstrates a novel approach to organizing the literature on virtual care, moving from the question of whether virtual care is as effective as in-person care for a given health problem to thinking about how virtual care supports the core functions of primary care. It argues for a different way of conducting studies in the future and provides a framework for organizing this research. Much of the included literature covers the period during the COVID-19 pandemic, when most ambulatory care was virtual in many jurisdictions. This context is not as broadly applicable to the current state of care. However, we believe that our findings still provide valuable insights into how virtual care can be leveraged in primary care and can suggest appropriate ways of structuring future studies to better understand when and how voice, video, and SMS text-based interactions are best used in clinical care.

### Conclusions

This scoping review found no consistent differences in the appropriateness of care or patient outcomes between virtual and in-person care across the included studies. Virtual care is perceived as equivalent to in-person care for many common uses, despite some variation by condition. The overall impact of virtual care will likely depend on whether it is used for triage, diagnosis, treatment, or counseling and whether patients or providers determine the choice of modality. Moreover, we found that the overall value of virtual care for the health system, including potential cost savings, remains unclear. Our findings highlight that although the rates of virtual care use may decrease as concerns about COVID-19 infection lessen, for functions such as triage, counseling, and rehabilitation, virtual care may remain the norm. Further studies, incorporating options for patient and provider choice, are needed to determine the optimal use of virtual care from a resource and outcome perspective. This comprehensive review is one of the first that aims to understand how virtual care can be leveraged in the various components of routine primary care functions. Given the recent changes in practice, this evidence base will continue to grow, and timely reviews will be needed to keep up with the literature. These modalities are no longer just a temporary measure to get through the pandemic and now need to be adapted to fit a new set of patient and provider workflows to demonstrate positive impacts on experience, outcomes, and costs of care. Future reviews should focus on when to use virtual modes of communication in a patient’s primary care journey.

## Appendix

Multimedia Appendix 1 PRISMA-ScR (Preferred Reporting Items for Systematic Reviews and Meta-Analyses extension for Scoping Reviews) checklist.

Multimedia Appendix 2 Search strategy and data extraction tables.

## Abbreviations

CBT: cognitive behavioral therapy
GP: general practitioner
PEBC: Program in Evidence-Based Care
PRISMA-ScR: Preferred Reporting Items for Systematic Reviews and Meta-Analyses extension for Scoping Reviews
RCT: randomized controlled trial

## Data Availability

Data sharing is not applicable to this paper as no datasets were generated or analyzed during this study.
