# Supplementary material for: Uses of Virtual Care in Primary Care: Scoping Review
Source: J Med Internet Res. 2025 Feb 14;27:e55007. doi: 10.2196/55007 (PMC11888022; doi:10.2196/55007)
Supplement: Multimedia Appendix 2 [file jmir_v27i1e55007_app2.doc]

# Literature search strategy.

Database: Embase <1996 to 2021 November 24>, OVID Medline Epub Ahead of Print, In-Process & Other Non-Indexed Citations, Ovid MEDLINE(R) Daily and Ovid MEDLINE(R) 1946 to Present, APA PsycInfo <1987 to November Week 3 2021>, EBM Reviews - Cochrane Central Register of Controlled Trials <October 2021>, Ovid Emcare <1995 to 2021 Week 46>

Search Strategy:

--------------------------------------------------------------------------------

1 exp Telemedicine/ or Remote Consultation/ or exp Teleconsultation/ or exp Telerehabilitation/ or exp Telehealth/ or (Telemedicine or Tele-medicine or Remote Consultation or Teleconsult: or Tele: Consult: or Phone: Consult: or Telerehabilitation or Telehealth or Tele-health or eHealth or e-Health or mHealth or m-Health).ti,kw,ab. (195369)

2 (exp Consultation/ or exp Disease Management/ or exp Patient Monitoring/ or exp Follow up/ or exp Patient Care/ or exp "Evaluation and Follow up"/ or exp Medical Examination/ or exp Physical Examination/ or exp Treatment Outcome/) and (exp Teleconference/ or exp Videoconferencing/ or exp telephone/ or mobile phone/) (87960)

3 ((virtual or distance or distant or remote or video: or phone or telephone or teleconferenc:) adj3 (consult: or manage: or monitor: or follow-up or care or evaluat: or exam: or treat: or intervene or intervention: or assess: or diagnos: or navigat: or "after-care" or deliver: or service: or contact:)).ti,kw,ab. (254219)

4 1 or 2 or 3 (459886)

5 4 not ((abstract or "conference abstract" or news: or case report: or comment or editorial or note).pt. or letter/ or conference abstract/ or single-case study/) (385091)

6 limit 5 to (English language or no language specified) [Limit not valid in APA PsycInfo,Ovid Emcare; records were retained] (365311)

7 limit 6 to yr="2014 -Current" (211330)

8 Primary health care/ or Primary medical care/ or General practitioners/ or General Practice/ or Primary care nursing/ or General practitioner/ or Physicians, family/ or Physicians, primary care/ or family nurse practitioner/ or (primary health: or primary medic: or general practi: or primary care or (family adj2 (physician: or doctor: or nurse: or medicine or practice:))).ti,kw,ab. or ((gp and (medic: or doctor: or physician: or patient:)) not (glycoprotein or gemcitabine)).ti,kw,ab. (955260)

9 pediatrician/ or pediatrics/ or (pediatrician or paediatrician).ti,kw. (215832)

10 psychologist/ or psychotherapist/ or social worker/ or social work/ or (psychologist: or psychotherapist: or social work:).ti,kw. or exp *counseling/ or exp *psychotherapy/ or exp *depression/rh, th [Rehabilitation, Therapy] (399177)

11 midwife/ or (midwife: or midwive:).ti,kw. or geriatrician/ or geriatrics/ or geriatrician:.ti,kw. or community pharmacist/ or (community: adj2 pharmacist:).ti,kw. or physiotherapist/ or physiotherapist:.ti,kw. (218811)

12 7 and (or/8-11) (25493)

Lines 13-47 to remove duplicates (14916)

Websites search for systematic reviews (March 2-16, 2022):

- WHO: <https://www.who.int/publications/who-guidelines>
- G-I-N library: <https://guidelines.ebmportal.com/>
- ECRI Database: <https://guidelines.ecri.org/>
- SIGN (UK): <https://www.sign.ac.uk/our-guidelines/>
- CMA Infobase: <https://www.cma.ca/En/Pages/clinical-practice-guidelines.aspx>
- NICE (UK) – NICE Guidance: <https://www.nice.org.uk/guidance>
- National Health and Medical Research Council Australia: <https://www.nhmrc.gov.au/guidelines>
- AHRQ (USA): <https://www.ahrq.gov/research/findings/evidence-based-reports/search.html?search_api_fulltext=mhealth>
- NIHR (UK) HTA: <https://evidence.nihr.ac.uk/browse-content/?_sf_s=virtual+care>
- CADTH (Canada): <https://www.cadth.ca/search?keywords>

The following search terms were used for the websites searched: virtual, telemedicine, ehealth, remote consultation, teleconsultation, teleconference, telerehabilitation, telehealth, telephone, mobile, smartphone, phone, video, online, in-person, distance, distancing.

**SUPPLEMENTARY MATERIALS**

Table S1 Primary studies on virtual/remote triage

Table S2 Systematic reviews on virtual/remote triage

Table S3 Primary studies of virtual care versus in-person care in general primary care

Table S4 Systematic reviews of virtual care versus in-person care in general primary care

Table S5 Primary studies of virtual care versus in-person care during COVID-19 restrictions

Table S6 Primary studies on virtual care versus in-person care of minor infections

Table S7 Systematic reviews on virtual care versus in-person care of minor infections

Table S8 Primary studies of virtual care versus in-person care in COVID-19 management

Table S9 Primary studies on virtual care versus in-person care in chronic disease management

Table S10 Systematic reviews on virtual care versus in-person care in chronic disease management

Table S11 Primary studies of virtual care versus in-person care in medical abortion

Table S12 Primary studies of virtual care versus in-person care in rehabilitation

Table S13 Systematic reviews of virtual care versus in-person care in rehabilitation

Table S14 Primary studies on virtual versus in-person counselling

Table S15 Systematic reviews on virtual versus in-person counselling

Table S1. Primary studies on virtual/remote triage

| **Source** | **Topic** | **Type of study** | **Population or number of patients** | **Results** |
| --- | --- | --- | --- | --- |
| Ho, 2021 [19]  Apr 6 – Aug 2, 2020 | HealthLink BC Emergency iDoctor-in-assistance (HEiDi) | Calls to 811 service in BC screened by registered nurses; those determined to require care within 24 hours were referred to HEiDI physicians by videoconferencing | 7,687 consultations by physicians  HEiDI visits: gastroenterology (n=1275, 16.6%), respiratory (n=877, 11.4%) and dermatology (n=874, 11.4%) | Of callers directed to videoconferencing with HEiDi physicians, 33.8% were advised to attempt home treatment, 38.3% to contact a primary care physician within 1 week, 15.0% to attend an ED immediately and 7.1% to contact their primary provider now |
| Newbould, 2019a,b [20, 21]; Ball, 2018 [22]; Newbould, 2017 [23]  July 2011-Apr 2016 | Telephone-first approach in England: views of GP/staff and patients | All patients speak to receptionist; those requesting GP appointment called back by GP prior to scheduling face-to-face appointment  Problem either resolved over telephone, seen by/referred to another healthcare professional, or given face-to-face appointment with GP (usually on same day) | 147 general practices using telephone-first plus random 10% sample of other practices; interviews with staff and patients; England | Reduced time for appointment from 3-4 weeks to 1 day  8% increase in GP interaction with patients, but less face-to-face; 2.0% increase in hospital admissions (p=0.006), no initial change in ED attendance, 2% per year decrease in the subsequent rate of rise of ED attendance (1% to 3%; p=0.005). |
| Miller, 2019 [24]  2014-2017 | Telephone-first consultation in general practice, UK | Interrupted time series of preplanned outcomes for 2 yr before and 1 yr post-introduction of a telephone-first system | General practice of 11,500 patients in deprived inner-city area, Northern Ireland; >79% contacted care team each year | Prior to telephone-first system, patients could not get appointments  The telephone-first system resulted in all face-to-face consultations occurring on the day of initial contact  20% increase in total GP consultations (telephone + face-to-face, p=0.001) after telephone-first system implemented  Telephone consultations increased by 131% (from 913 to 2523/month; p<0.001) and face-to-face consultations decreased by 39% (from 2394 to 1472/month; p<0.001) |
| Jiwa, 2002 [25]  Jul 1999 – Jun 2000  Control years 1997-1998 | Telephone-first consultation by GP, UK | Interrupted time series, 2 yr before and 1 yr after introduction of GP-led telephone triage  All patients requesting same-day appointments were told that a GP would call them later | Group practice in a market town, UK. Levels of deprivation were close to the national average (Jarman score is ‑4.26)  3,680 calls in second half of study | Demand for face-to-face appointments with a GP was reduced by 39% (p<0.001)  >92% of telephone calls lasted <5 min  For a selected month, 43.3% of callers were offered same-day appointments, 29.3% advice only, 22.4% prescription without face-to-face consultation, 2.4% visit, 4.1% routine appointment (not same day), 0.8% visit with nurse |
| Edwards, 2017 [26]; Farr, 2018 [27]  April 2015-June 2016 | Primary care online consultation system in South West England (One Care): interview with staff and patient survey | Patients accessed self-help, pharmacy advice, 111 (National Health Service non-emergency telephone advice), administrative help (such as repeat prescriptions) or submitted an online form to GP with details of their condition  If the system identified signs or symptoms that required immediate medical attention patients were redirected to appropriate services; otherwise, patients were informed that GP would contact them by the end of the next working day | eConsult system in 36 general practices accessed through own GPs website  7,472 e‑consultations submitted, including 22.5% administrative requests (repeat prescriptions, test results, letters)  Random sample of 485 patient looked at in detail | Some administrative triage: calls allocated to GP who decided on action (38% face-to-face, 32% telephone call) or to administrative staff  Main issues were musculoskeletal/ limb pain, infection/ immunological, neurological, sexual/reproductive health, dermatological, respiratory, mental health, digestive, medication query, administrative (notes, prescription renewal, test results) |
| Holt, 2016 [28]; Varley, 2016 [29]; Warren, 2015 [30]; Calitri, 2015 [31]; Campbell, 2015 [32]; Campbell, 2014 [33]; Murdoch, 2015 [34]  2011-2013 | ESTEEM Trial  Telephone triage in UK general practice | RCT; triage requests for same-day appointments were randomized to triage by nurse (supported by computer decision support software) vs. GP vs. usual care.  Duration of face-to-face contacts with a GP after a triage call were compared with those occurring in usual care  Patients reported experiences via postal questionnaire | 15,394 patients (5,138 in usual care; 5,001 in GP triage; 5,255 in nurse triage)  Participants were consecutive patients (aged ≥16 yr or <12 yr) seeking a same-day face-to-face consultation with a GP | Initial contact time: GP triage 4.0 (±2.8) min; nurse triage 6.6 (±3.8) min; and usual care 9.5 (±5.0) min  Estimated overall contact duration (triage + subsequent contacts on the same day) 10.3 min for GP triage, 14.8 min for nurse triage, and 9.6 min for usual care  Triaged patients more likely to require further consultations over the subsequent 28 days  Nurse practitioners compared to practice nurses were less likely to recommend patients for follow-up (OR=0.19); nurse practitioners were more likely to definitively manage the patient  The number of deaths, emergency hospital admissions, and accident and ED attendances were not significantly different |
| Lawless, 2016 [35]  2013-2015 | General practice, Greenwich, London, UK | 3 pilot approaches in 3 to 5 practices each: GP telephone triage of all appointment requests; analysis and comparison of practice data including demand and capacity; online self-help and GP consultation as last resort |  | Waiting time reduced from 4 days (range 1-6 days) to <1 day in scheme 1; acceptability to GP and staff varied between practices |
| Villarreal, 2017 [36]  Oct 2012-Nov 2013 | Partnership between GP and ambulance services to reduce transport to EDs, Worcestershire, West Midlands, England | GP-supported assessment at scene or by telephone for non-trauma cases  Triage by emergency call centre to paramedic crew team and/or on-call GP who attends in own vehicle; ambulance crew could also contact on-call GP for telephone advice or request attendance | 23,395 emergency contacts of which 1903 (8.1%) triaged to GP- supported assessment (1221 face-to-face, 682 telephone) | 78.8% of those with GP support not transported to hospital |
| Siddiqui, 2017 [37]  Dec 2014 baseline; Feb-April 2015 | Active signposting to streamline GP workload | Quality improvement project to investigate types of consultations and whether essential (GP required) or possibly avoidable (GP not required)  New intervention with reception staff signposting non-essential cases to other allied professionals, non-medical staff, alternate services | 2 practices in London (UK) | Many telephone consultations (TC) booked with GP could be signposted to self-help or alternative services  After 2 Plan, Do, Study, Act cycles, the proportion of essential consultations taking place at both practices increased from 28.6% and 27.3% at baseline, to 82.6% and 71.4%, respectively. A lower number of possibly avoidable teleconsultations equates to more time for essential teleconsultations and greater value for money |
| Elliot, 2020 [38]  Dec 2015-Nov 2017 | Nurse-led triage in primary care | 2 studies: 2-yr pilot of standard nurse-led triage to manage all same-day appointment requests either in person or via telephone; 6-month pilot of total nurse triage which managed appointment requests for both same-day and routine appointments made by telephone | Standard triage in 4 practices in South Powys GP cluster in Wales; total triage subsequently in one of these practices  South Powys cluster in Wales  24,060 patients with standard triage (72% telephone and 28% face-to-face); 5,298 telephone encounters in total triage | Possible clinical outcomes for patients who had undergone either of the two nurse-led triage services included: a routine appointment with a GP in an appropriate timescale; face-to-face treatment or advice from a nurse; encouragement to self-manage; referral to emergency services; or direction to a pharmacist, optician, physiotherapist, or other healthcare professional  In the standard nurse-led triage service, a total of 13,113 GP appointments were saved over the study  11% of telephone and 9% of face-to-face triage resulted in routine GP appointment; 35% of telephone and 25% of face-to-face triage resulted in same day GP appointment; 23% of telephone and 0% face-to-face resulted in nurse appointment  In the Total Nurse Triage service, 2,270 GP appointments were saved over the study period by directing patients to other services; 57% of patients received GP appointments; waiting time for GP appointments were reduced to 48-72 hours from 2-3 weeks |
| Huibers, 2016 [39]  Jul 2010 – May 2011 | Telephone triage by GPs in out-of-hours primary care | GPs perform most triage in Denmark  Data from a prospective observational study, the LV-KOS study: GP filled questionnaire about a subset of calls; GPs with in-person visits assessed whether it was necessary | 4,620 telephone consultation only; 3,190 triaged to face-to-face | 59.2% of calls ended with a telephone consultation  Patient age >40 yr (40–64: RR=1.13; >64: RR=1.34), persisting problem for 12–24 hours (RR=1.15), severe problem (RR=2.60), potentially severe problem (RR=5.81), and non-severe problem (RR=2.23) associated with triage to a face-to-face contact  Face-to-face contacts assessed as irrelevant for 12.7% of clinic consultations and 11.7% of home visits  Statistically significant higher risk of irrelevant face-to-face contact was found for a persisting problem of >24 hours (RR=1.25), contact on weekday nights (RR=1.25), and contact <2 hours before the patient’s own GP’s opening time (RR=1.80) |
| Jansen, 2021 [40]  Jan-Dec 2017 | Income-related differences in out-of-hours primary care telephone triage | Retrospective data derived from Nivel Primary Care Database linked to sociodemographic population registry data and analyzed using multilevel logistic regression analyses | Electronic health record data of 1.3 million patients from 28 OPCSs in 2017 in the Netherlands | Telephone triage conducted by certified and medically trained triage nurses supervised by a GP  Patients contacted an OPCS by telephone, then the triage nurse formed an impression of the health condition of the patient by enquiry  The most frequently presented symptoms deduced during triage slightly differed across socioeconomic status groups, with a larger relative share of trauma in the high-income groups; no socioeconomic status differences were observed in urgency assessment  Low income was associated with a higher probability of receiving telephone advice and home visits, and fewer consultations at the OPCS. |
| Eccles, 2019 [41]  May 19-Jul 31 2017 | Patient use of an online triage platform | Retrospective analysis of routinely collected data in UK primary care | All practices using the ‘askmyGP’ platform, National Health Service, UK  5,447 patients | Users accessed the ‘askmyGP Version 2’ platform via their GP website and completed an online form detailing their background and query using a combination of tick-boxes and free-text boxes  Receptionist either completed an administrative request or passed a medical request to a GP, who then arranged either a face-to-face or telephone consultation with the patient; requests outside of practice opening hours were processed the next working day.  Highest levels of use were observed in females (65.5%, n=3,570) and those aged 25–34 yr  Patterns of use were high between 08:00 and 09:59 and on Mondays and Tuesdays; use outside of GP practice opening hours was low  Frequently used for medication-related enquiries, for administrative requests, and to report a specific symptom |
| Christensen, 2016 [42] | Drug prescriptions in out-or-hours primary care cooperatives | Population-based retrospective observational study using registry data | All contacts to out-of-hours primary care in Central Denmark Region  644,777 contacts; 24.0% with medication prescriptions | All contacts triaged by a GP by telephone and then either handled as telephone consultation or referral to clinic consultation or home visit  Medication prescriptions: 21.9% of telephone consultations, 32.9% of clinic consultations, 14.3% of home visits. Around 53% of all drug prescriptions were made in telephone consultations  Prescription by type: 45.5% anti-infectives for systemic use, 10.5% ophthalmological anti=-infectives, 6.4% NSAIDs, 3.9% opioids, 3.0% adrenergic inhalants, 2.3% antihistamines |

Abbreviations: ED, emergency department; GP, general practitioner; NSAID, nonsteroidal anti-inflammatory drug; OPCS, out-of-hours primary care service; OR, odds ratio; RCT, randomized controlled trial; RR, relative risk

Table S2. Systematic reviews on virtual/remote triage

| **Source** | **Topic** | **Patient population** | **Number of included RCTs** | **Number of included non-RCTs** | **Review conclusions** |
| --- | --- | --- | --- | --- | --- |
| **Triage** | | | | | |
| Lake, 2017 [43] | Telephone triage | Patients who received telephone-based triage and general practitioner consultation related to general primary care | 10 systematic reviews included |  | Current evidence does not provide definitive answers about the quality of care provided, access and equity of the service, costs, and outcomes  Evidence suggests interactional factors (e.g., relationship with other health service providers) which can impact on measures of performance, and affect the external validity of findings |
| Rushton, 2019 [44] | Remote triage | Patient or family member initiating remote triage services focused on a clinical care issue | 5 | 30 (including 4 systematic reviews) | Remote clinical triage systems may reduce medical workload, improve access to primary care advice, reduce inappropriate use of urgent and emergency department services, but may not be meetings its goals  Limited evidence suggests remote triage reduces the burden on primary care and subsequent use of emergency department services |

Abbreviations: RCT, randomized controlled trial

Table S3. Primary studies of virtual care versus in-person care in general primary care

| **Source** | **Topic** | **Type of Study** | **Population** | **Number of patients** | **Results and conclusions** |
| --- | --- | --- | --- | --- | --- |
| **General practice (various)** | | | | | |
| Dixon, 2009 [45]; Stahl, 2010 [46]  Sept 2007-Mar 2008 | Virtual (videoconference) visits in general medicine | RCT, randomized cross-over design  Arm 1: patients completed a visit (virtual or face-to-face) with a physician; they then completed a second visit via the other modality with another physician.  Arm 2: subjects had both visits face-to-face; different physicians conducted the two face-to-face consultations | Patients aged 18-85 yr with range of commonly presenting problems in adult primary care practice in Boston.  The most common complaints included follow-up for chronic diseases including hypertension, elevated cholesterol and diabetes. Acute illnesses that subjects presented with included musculoskeletal complaints and upper respiratory illness/sinusitis. | 175 scheduled, 152 participated | Virtual and face-to-face visits were found to be similar on most measures, including, time spent with the physician, ease of interaction, and personal aspects of the interaction  High physician satisfaction with the virtual visit modality  The diagnostic agreement between physicians was 84% between face-to-face and virtual visits; it was 80% between the two face-to-face visits |
| McKinstry, 2002 [47] | Telephone consultation for same-day appointments | RCT  Randomized to either a face-to-face appointment on that day or to a callback that morning by doctor who, after discussion, would either offer them advice or treatment on the telephone or, if necessary, would arrange to see the patient later in the day. | All patients phoning for same-day appointments for themselves or their children in each surgery in two urban practices (total population = 10,420) over a four-week period | 388  Patients specifically asking to speak to the doctor by telephone for advice, those deemed very urgent cases, and those with no contact telephone number were to be excluded | Consultation duration was 6.7 min telephone vs. 8.2 min face-to-face, p=0.002  Reconsultation in subsequent 2 weeks was 0.6 vs. 0.4 consultations per patient, p=0.01  Blood pressure was more frequently measured in the face-to-face appointments (13.3% vs. 6.6%)  No significant differences in secondary outcomes, including patient perceptions |
| McKinstry, 2010 [48] | Quality, safety, and content of telephone vs. face-to-face consultation | 106 audio-recordings (from 19 doctors in nine practices) of five consecutive telephone and five face-to-face consultations  Results stratified at doctor level, were compared using the Roter Interaction Analysis Scale (content measure), the OPTION (observing patient involvement in decision making scale) and a modified scale based on the Royal College of General Practitioners consultation assessment instrument (measuring quality and safety) | Doctors used portable digital recorders for the face-to-face consultations so that the entire conversation from the time the patient was called from the waiting room was recorded. A similar recorder, linked to a bridge device, recorded telephone calls | 105 patients; 46 telephone and 59 face-to-face recordings matched at doctor level | Telephone consultation use ranged from 14% to 40% across participating practices, with a median of 24%  45 (37%) telephone consulters and 43 (30%) face-to-face consulters did not return their second written consent to analyze recorded visits  Significant differences between telephone and face-to-face consultations included shorter duration (4.6 vs. 9.7 min, p<0.001), fewer problems presented (1.2 vs. 1.8, p<0.001), less data gathering (p<0.001), less counselling/advice (p<0.001), and less rapport building (p<0.001)  Telephone consultations were less likely to include sufficient information to exclude important serious illnesses.  Patient involvement and patient satisfaction outcomes were similar |
| Gujral, 2021 [49]  2014-2018; 2013 control prior to V-IMPACT | Telehealth to improve access in primary care | Observational longitudinal study with repeat patient cross-sections  V-IMPACT program set up regional hub that provides primary care via telehealth to patients in outlying spoke sites | Veterans, Veterans Health Administration (VHA) with primary care team, regional hub in Boise (Idaho) and spokes across Washington, Idaho, Oregon, Alaska, and Montana | 208,612 veterans at 22 spoke sites | Patients attended spoke clinic for video visits and often seen by nurse or other staff who assist with examination and video encounter with hub physician  The analysis adjusted for patient characteristics: age, VHA enrollment priority, gender, marital status, race/ethnicity, hierarchical condition category (HCC), and drive distance to patients’ closest VHA primary care and secondary care sites  Increased telehealth visits for primary care (IRR=2.42) and primary care mental health (IRR=7.25) after V‑IMPACT adopted  V-IMPACT not associated with acute hospital stays, emergency department visits, or VHA costs |
| Llorian, 2021 [50]  2007-2016 | Healthcare utilization and telemedicine in Manitoba | Retrospective observational study using administrative data from Manitoba Health Insurance Registry, Manitoba Population Research Data Repository, MBTelehealth Programme; propensity-weighted regression for telemedicine users and non-users | Included in Manitoba Health Insurance Registry, had Manitoba health coverage from December 2007–December 2016, and 18 yr of age or older | 15,663 telemedicine (video) at least once; 257,132 never used telemedicine | Telemedicine users participated in videoconferencing from a television screen at a healthcare site for visit with remote physician  Compared to non-users, telemedicine patients showed a higher number of in-person visits. Using incidence-rate ratios: 1.32 more ambulatory visits, 1.26 more visits to primary care practitioners 1.38 more visits to specialists and 1.14 more hospitalizations  Patients who participated in frequent virtual visits with a specialist had decreased in-person visits with the same type of care (incidence rate ratio=0.48)  There were differences in utilization patters between telemedicine users and non-telemedicine users |
| Reed, 2021 [51]  Jan 2016-May 2018 | Physician prescribing in primary care telemedicine | Cohort study of all completed primary care appointments booked through patient portal, Kaiser Permanente Northern California  Patients booking through patient port select office, video, or telephone visit except for routine physical exams in-person only (not included in this study) | All patients who scheduled primary care appointments through patient portal of primary care in large integrated health system, Kaiser Permanente Northern California | 1,131,722 patients, 14% of visits by telemedicine; 7% of telemedicine visits were by video | Appointment availability was generally within 3 days, often same day, and was similar across the visit types  Covariate adjusted data: medication was prescribed for 38.6% of video visits, 34.7% of telephone visits, and 51.9% of office visits; laboratory tests or imaging were ordered for 29.2% of video visits, 27.3% of telephone visits, and 59.3% of clinic visits; antibiotics were prescribed in 10.6% video, 9.7% telephone, 13.5% office visits; follow-up visits occurred within 7 days in 25.4% of video visits, 26.0% of telephone visits, and 24.5% of office visits  There were no statistically significant differences in emergency department visits and rates of hospitalization between the groups. |
| Graetz, 2020 [52]  Jan 2016-May 2018 | Patient choice of telemedicine, wait time | Observational, multivariate logistic regression; association between visit type and timeliness of appointment | Patient-initiated appointments with own primary care provider, Kaiser Permanente Northern California | 2,178,440 primary care visits by 1,131,722 patients  14% telemedicine (telephone or video) | Patients chose their own visit type: office, video, telephone; each visit type had similar scheduling availability except for ‘routine physical’ which was only offered in office  Visits were categorized as timely if the visit took place before the end of the following calendar day after the scheduling of the appointment  The mean calendar days after scheduling to the actual date of the appointment was 3.52 days for in-person visits, 2.29 days for video visits, and 1.80 days for telephone visits; adjusted data indicated 46.49% in-person, 56.58% video, and 66.61% telephone visits were scheduled within 1 day |
| Reed, 2020 [53]  Jan 2016-May 2018 | Patient characteristics in telemedicine visits | Cross-sectional study: multinomial logistic regression to examine the association between the chosen visit type (using office visit as the reference type) and patient characteristics | Patient-initiated appointments with own primary care provider, Kaiser Permanente Northern California | 2,178,440 primary care visits by 1,131,722 patients  14% telemedicine (telephone or video) | Patients chose visit type: office, video, telephone.  Telemedicine was statistically significantly associated with patient sociodemographic characteristics (age ≥65 yr less likely than patients 18-45 yr to choose telemedicine), internet access in neighborhood, and barriers to in-person visit |
| Ryskina, 2021 [54]  March -May 2020 | Older adult primary care and telemedicine disparities | Retrospective cross-sectional study; multivariable logistic regression | Primary care patients, 32 clinics in Mid-Atlantic states in USA  Abstract indicates age ≥65 yr, text indicates both ≥50 yr and ≥65 yr | 17,103 patients; 60.3% had first visit by telemedicine  Scheduled patients were called by nurse to determine urgency and whether in-person visit was needed; preferentially triaged to telemedicine unless strong preference or physical examination required | Measured the odds of being seen via telemedicine vs. in person as a function of patient demographics, comorbidities, and week of study period; telemedicine included a video option but not required (could use telephone)  60.3% of patients used telemedicine; higher in Black patients (aOR=1.30) and lower in Hispanic patients (aOR=0.63)  Patients using telemedicine had lower odds of ambulatory care sensitive conditions hospitalization (aOR=0.78), but Black patients with telemedicine had higher odds of hospitalization (OR=1.43) than White patients |
| Cecil, 2021 [55]  April 2014 – Dec 2017 | Acute deterioration in primary care | Population-based observational study | Patients of all ages who experienced an acute deterioration in health resulting in emergency hospital admission within 3 days of a general practitioner consultation, England | 116,097; of these 87% face-to-face, 19.8% telephone, 4.7% out-of-hours | Potentially missed acute deterioration defined as patient having a self-referred admission to a hospital within 3 days following a primary care visit with a GP: patients with sepsis (aOR=1.09) or urinary tract infections (aOR=1.09) were more likely to self-refer to a hospital  Differences in duration of appointments with GP was associated with subsequent self-referral admissions, where an average 5-minute increase in appointment time resulted in a 10% decrease (aOR=0.90)  19.5% of admissions had a previous hospital admission within 30 days  Patients having a telephone consultation (compared with face-to-face consultation) (aOR=1.14) previous health service use, and presence of comorbidities were also associated with self-referred admission  18.8% of patients admitted with ectopic pregnancy, 35.3% with pulmonary embolism, 28.7% with sepsis, and 33.7% with urinary tract infections had contact with general practitioner within 3 days before admission |
| Gonzalez, 2018 [56]  2014-2015 | Telephone consultation in primary care | Retrospective longitudinal observational study | Primary care GP consultations, age ≥15 yr, in Galicia (region in the Northwest of Spain) | 28,472,852 consultations; 9.0% telephone | Patient set up appointments on web and could choose face-to-face or telephone consultation  Consultations increased with age; face-to-face in 2014 was 2.92 consultations/inhabitant for age 15-25 yr and 9.12 for age 76-85 yr; for telephone was 0.18 consultations/inhabitant aged 15-25 yr and 1.44 age >85 yr. There was a 35% increase from 2014 to 2015 in telephone consultations.  8.6% of patients missed face-to-face appointments; 0.7% missed telephone appointments  Women requested more telephone consultations (9.9% of consultations) than men (7.7%)  Telephone consultations in urban areas (0.53 and 0.69 per person for years 2014 and 2015) greater than rural areas (0.34 and 0.47)  10.9% of telephone consultations required subsequent face-to-face consultation |
| Ross, 2016 [57]  Sept-Dec 2015 | Pilot project of telehealth for primary care access in Northern Alberta | Telehealth for primary care; private consultation space in community | Two First Nations communities in northern Alberta | 60 patients | Licensed practical nurse screened patients for appropriateness of telehealth; remote physician conducted physical exam with control of high-resolution camera and assistance of community health representative if needed to help with digital stethoscope or other assessment tools  Most frequent concerns were rashes (36%), prescription refill (24%), pain (14%), upper respiratory tract infection/flu (10%), contraception, anti-hypertensives  17% no-show rate, compared to 4.8% to 6.6% previously with clinic open 3-4 days every 6-8 weeks  Pilot project demonstrated primary care telehealth is feasible and sustainable; additional equipment (otoscope) could expand scope  Only 5 patients referred to Slave Lake Family Care Clinic that is >280 km away; travel and time saved by this initiative |
| Bernstein, 2021 [58]  Nov 2015 – Mar 2019 | Urgent and non-emergent telehealth care for seniors | Retrospective cohort, telehealth for initial (index) visit vs. in-person primary care or office index visit | Age ≥60 yr  3 health systems in USA; 2 systems used structured models for specific conditions (20 or 8 categories, 1 used all-calls approach (13 conditions) | Index Visits: 313,516 telehealth visits (Kaiser Permanente 313,241 visits, 20 conditions; Jefferson Health 135 visits, 8 conditions; Spectrum Health 140 visits, 13 conditions). In-person 2,597,132 visits; 1025 visits; 1423 visits | Main outcomes were visit resolution and episodes of care.  Telehealth encounters were successful in resolving urgent and non-emergent needs in 84.0–86.7% of cases  When visits required follow-up, over 95% were resolved in less than three visits for both telehealth and in-person cohorts.  Resolution: Kaiser Permanente 86.7% vs. 90.8%; Jefferson Health 84.0% vs. 90.7%; Spectrum Health 85.7% vs. 91.3% |
| Lovell, 2021 [59]  April 1, 2016 to March 31, 2017 | Low-acuity urgent conditions | Retrospective, cross-sectional review of virtual care at Intermountain Healthcare (a not-for-profit integrated healthcare system in Utah and Southern Idaho that includes hospitals, clinics, employed caregivers, an insurance organization, and a system-sponsored telemedicine programme) | Claims in SelectHealth (owned by Intermountain Healthcare) for the nine most common diagnosis categories for virtual care: sinusitis, conjunctivitis, urinary tract infection, upper respiratory infection, influenza/ pneumonia, bronchitis, dermatitis/eczema, ear pain, digestive symptoms, and cough; patients ≤65 yr | 1,531 virtual care  45,344 urgent care ; 4377 matched  126,932 primary care practice, 4388 matched  9046 emergency department, 2285 matched | Follow-up rates were similar between virtual care and urgent care  There were no differences between virtual care and urgent or primary care in antibiotic use  Utilization of laboratory and imaging services, index visit cost and total costs over 21 days were significantly lower in virtual care than any other care setting |
| Ohta, 2017 [60] | Diagnosis: telediagnosis vs. face-to-face | General medicine outpatients at Chiba Prefectural Togane Hospital (Japan); diagnosed face-to-face; complaint, medical history, vital signs shared with 2^nd^ doctor who then did teleconference assessment | General medicine outpatients, age 19-84 yr (mean 52 yr) | 97 patients, | Note that this was not complete virtual assessment, as first face-to-face doctor shared information patient history, complaint, and vital signs instead of these being determined by videoconference assessment  The correct diagnosis rate was 80.4% (78/97 cases) for teleconference and 82.5% (80/97 cases) face-to-face  The telediagnosis group was correct in 32% (8/25) of the cases in which the telediagnosis and face-to-face diagnoses did not agree, compared to 40% (10/25) in the face-to-face group |
| Gordon, 2017 [61]  Jan 2014 – May 2015 | Insurance claims for conditions of interest  11 most common through telehealth platform: sinusitis, upper respiratory infection, urinary tract infection, conjunctivitis, bronchitis, pharyngitis, influenza, cough, dermatitis, digestive symptoms (nausea/vomiting/diarrhea), or ear pain | Cross-sectional retrospective study of claims from large health insurer receiving care for condition of interest  Matched virtual care visits to other settings (other settings matched 3:1 on acute condition, geography, child/adult)  Episode = index visit pls 3 weeks following | Adults <65 yr of age, children >6 months, no serious/ expensive health conditions  Patients at retail health clinics (RHC), urgent care centres (UCC), emergency departments (ED), or primary care physicians (PCP) for acute non-urgent conditions. | 59,945 visits: 4635 virtual, 55,310 non-virtual; USA  General linear models with gamma distribution and log link were used to compare costs and were adjusted to account for differences in age category and common baseline comorbidities | Care provided was assessed by follow-up outpatient visits, ED visits, or hospitalizations; laboratory tests or imaging performed; and antibiotic use after the initial visit. Episode costs included the cost of the initial visit, subsequent medical care, and pharmacy.  During the episode:  **Antibiotic fills (excluding influenza) within 3 days**: higher in the virtual group (70.51%) compared with all other sites (RHC 64.18%; p<0.001, UCC 67.94%; p=0.02, ED 56.73%; p<0.001, PCP 68.19%; p=0.03); varied by infection type  **Follow-up visits**: virtual care group (28.09%), RHC (28.59%; p=0.54), PCP groups (28.10%; p=0.96), UCC (25.62%; p<0.001), ED (34.19%; p<0.001)  **ED visits within 3 weeks**: virtual care group (1.32%) lower than UCC (2.68%; p<0.001), ED (6.47%; p<0.001), and PCP groups (1.84%; p=0.02) but similar to the RHC group (1.61%; p=0.14).  **Hospitalizations within 3 weeks**: virtual group (0.15%) lower than the UCC (0.41%; p=0.01), ED (0.96%; p<0.001), and PCP groups (0.37%; p=0.02) and similar to the RHC group (0.28%; p=0.12).  **Lab test rates** for virtual visits (12.56%) were lower than in-person locations (RHC: 36.79%, p<0.001; UCC: 39.01%, p<0.001; ED: 53.15%, p<0.001; PCP: 37.40%, p<0.001)  **Imaging rates** for virtual visits (6.62%) were typically lower than in-person locations (RHC: 5.97%, p=0.11; UCC: 8.77%, p<0.001; ED: 43.06%, p<0.001; PCP: 11.26%, p<0.001)  **Cost**: RHC, UCC, ED, and PCP were estimated to be $36, $153, $1735, and $162 more expensive than virtual visit episodes, respectively, including medical and pharmacy costs.  Limitations: cannot determine disease severity from a diagnosis code so ED, for example, may have been more severe; no information on reason for choosing site of care; no information on patient characteristics |
| **Emergency** | | | | | |
| Grech, 2017 [62]  Sept 2012 – Aug 2013 | Emergency contact after pediatric clinic visit | Retrospective chart review of pediatric ED visits | 368 children with contact with general pediatric clinic at Michigan Medicine within 2 days preceding ED visit | 474 clinic contacts (149 in-person, 216 telephone when clinic open, 109 telephone when clinic closed) | In-person visits with advice to go to an ED resulted in significantly greater rates of testing and admission than consultations by telephone with advice to visit an ED or for patients never advised to go to the ED |
| **Paramedic-led** | | | | | |
| Abrashkin, 2021 [63]  2015-2017 | Emergency treatment in home by paramedics in consultation with clinician or transport to ED | Retrospective: telemedicine-capable community paramedicine program in New York state operating within an advanced illness management program that provides home-based primary care | 2,000 housebound predominantly older individuals with multiple chronic conditions, activities of daily living dependencies, and high rates of acute care use in the previous year who enrolled in advanced illness management program | 1,707 paramedicine responses (899 video, 808 telephone contact with physicians) to patients in program who call special clinical call centre staffed by a nurse instead of calling 911 | Odds of ED transport between community paramedicine responses with video vs. telephone communication to a clinician was the primary outcome  Video availability had no significant effect on ED transport (OR=0.80. 95% CI=0.62-1.03); video enhanced clinical evaluation 85% of time but did not affect odds of ED transport |
| **Pharmacists** | | | | | |
| Al Mazrouei, 2021 [64]  Aug 1 to Oct 1, 2020 | Pharmacist interventions for abuse of over-the-counter medications | Prospective observational study, pharmacies with remote/ virtual services (remote consultation, refills, home delivery, medication review) vs. without | 12 community pharmacies in the United Arab Emirates |  | Pharmacist interventions on over-the-counter medicines with abuse and misuse potential respectively occurred at a frequency of 83.2% and 79.8% in pharmacies with virtual services and 91.0% and 41.2% in pharmacies without virtual services  19.7% of interventions were clinically significant in pharmacies with virtual services compared to 10.5% without  Pharmacies with virtual care dispensed significantly more cough medicines (25.6% vs. 9.7%, p=0.04)  Pharmacies with virtual services were more likely to ask patients to seek the advice of an addiction specialist over refusing to sell the drug (aOR=4.11; p=0.001)  Antihistamines and analgesics most common with abuse potential |

Abbreviations: OR, odds ratio; LSUP, low-sensitivity urine pregnancy test; RCT, randomized controlled trial; VHA , Veterans Health Administration; IRR, incidence rate ratio; aOR, adjusted odds ratio; ED, emergency department

Table S4. Systematic reviews of virtual care versus in-person care in general primary care

| **Source** | **Topic** | **Patient population** | **Number of included RCTs** | **Number of included non-RCTs** | **Review conclusions** |
| --- | --- | --- | --- | --- | --- |
| **Not specific context/disease** | | | | | |
| Carrillo de Albornoz, 2021 [65] | Teleconsultations in primary care | Adults receiving telephone/video consultations for primary care and mental health services | 5 | 6 | Teleconsultations via telephone or videoconference are an effective alternative for primary care and mental health services  Teleconsultations have the potential to deliver time-efficient and lower-cost interventions at a distance while improving access to healthcare |
| Hui, 2022 [66]  CADTH health technology review | Virtual care; limited to 2019-Oct 2021 publications | Patients using any forms of communication or information technologies to receive remote care | 3 systematic reviews [2 included elsewhere [65, 89] and 1 not relevant] | 8 non-randomized comparative studies, 7 grey literature publications | Virtual care decreased symptom severity in primary care with integrated mental health services and had a similar effect compared to in-person care for depression |
| Gray, 2021 [67]  CADTH Horizon Scan | Direct-to-patient virtual visits | Patients receiving health care services in a second location using videoconferencing, secure messaging, or audio digital tools. | No search details provided |  | Virtual visits could overcome many barriers associated with in-person care, including improved access, convenience, and cost savings  Risk that health inequities will worsen if barriers such as reliable access to an internet-connected device, technology literacy, and language barriers are not addressed |
| Totten, 2019 [68]; discussed in Totten, 2020 [69]  Agency for Healthcare Research and Quality (USA) | Telehealth for acute and chronic care | Patients using telehealth for acute and chronic care consultations | 44 | 190 | Telehealth consultations can improve outcomes or provide services with no difference in outcomes; evidence is stronger for some applications  Telehealth can expand critical care, speed emergency care decisions, and replace much face-to-face care while reducing exposure to infection |
| Totten, 2016 [70]; discussed in Totten, 2020 [69]  Agency for Healthcare Research and Quality (USA) | Telehealth | Patients receiving care through a telehealth modality for any clinical focus | 58 systematic reviews included |  | There is sufficient evidence to support the effectiveness of telehealth for specific uses with some types of patients, including remote patient monitoring for patients with chronic conditions, communication and counselling for patients with chronic conditions, psychotherapy as part of behavioural health |

Abbreviations: CADTH, Canadian Agency for Drugs and Technologies in Health

**Table S5. Primary studies of virtual care versus in-person care during COVID-19 restrictions**

| **Source** | **Topic** | **Type of Study** | **Population** | **Number of patients** | **Results and conclusions** |
| --- | --- | --- | --- | --- | --- |
| **COVID-19 restrictions** | | | | | |
| Schifeling, 2020 [71]  Apr 23 to May 22, 2020 | Video vs. telephone visits during COVID-19 restrictions on in-person visits | Retrospective, cross-sectional study of patients at 2 geriatric clinics; chart review (randomly selected) of ≈25% of patients with telephone and video appointments | Geriatric primary care in Colorado (average age 82.5 yr); telemedicine only implemented after start of COVID-19 pandemic  Patients who requested a routine or acute care visit were offered choice of video or telephone-based visit | 190 appointments: 47.4% video | Videoconferencing appointments were longer by an average of 7 min (p<0.001) and had more visit diagnoses (p=0.001)  No differences were found between the groups for rates of advance care; hearing, vision, and cognitive impairment did not affect appointment type  White patients, those who did not need interpreter, and those without Medicare were more likely to have video visits (p=0.003, p=0.01, p<0.001)  Patients participating in videoconferencing were younger (mean 81.3 yr vs. 83.5 yr, p=0.01), more likely to have an active patient portal account (59.1% vs.40.9%, p<0.001), and more likely to be accompanied by a caregiver during the video appointment (64.6% vs. 35.4%, p=0.01)  71.1% (64/90) of video visits included visual observation-dependent findings what could not have been assessed by telephone. The most cited reason for not using video was a lack of equipment (54/100, 54%), with other common reasons including patient preference (32%, 32/100) and cognitive problems (23%, 23/100). |
| Eberly, 2020 [72]  March 16-May 11, 2020 | General practice – demographics | Retrospective medical record review, all patients scheduled for telemedicine visits in primary care and specialty ambulatory clinics at a large academic health system (University of Pennsylvania; covers patients in Pennsylvania and New Jersey) | Primary and Specialty Ambulatory Care During the COVID-19 Pandemic | 148,402 unique patients with scheduled telemedicine visits; 80,780 (54.4%) completed visits (35,824 (45.6%) were conducted via video, whereas 42,715 (54.4%) had telephone visits | In multivariable models:  Older age (aOR of 0.67, 0.75, 0.85 for ages ≥75 yr, 65-74, and 55-64 yr), Asian (aOR=0.69), non-English preferred language (aOR=0.84), Medicaid (aOR=0.93) were associated with less completion of telemedicine appointments  Less video use was found in those of older age (aOR of 0.49, 0.78, 0.79 for ages ≥75 yr, 65-74 yr, and 55-64 yr female sex (aOR= 0.92), **B**lack race (aOR=0.65), Latinx ethnicity (aOR=0.90), and lower household income (aOR=0.57 for income <$50 000; aOR=0.89 for $50 000-$100 000) |
| Schenker, 2021 [73]  March 15 to Aug 31, 2020; March15 to Aug 31, 2019 pre-COVID comparison | Telehealth inequity in pediatric primary care - demographics | Retrospective pre-post cross sectional cohort study.  Changes due to COVID-19. Compared in person and video visits (exclude telephone calls) | The University of California, San Francisco (UCSF) Pediatric Clinic at Mount Zion | 5385 in 2020  6576 in 2019  In 2020: 2595 in-person and 3727 video | There were significant differences in patient demographics pre-COVID-19 and post-COVID-19  Patients were more likely to be younger, White/Caucasian or Asian, English speaking, and have private insurance during COVID-19 compared to pre-COVID-19  Older age was a significant positive predictor of having a video visit and public insurance was a significant negative predictor in a multivariate regression. |
| Lopez Sequi, 2021 [74]  2019 and 2020 | Variations in types of primary care visits before and during the COVID-19 pandemic | Retrospective, pre-COVID-19 and during COVID-19 to determine types of primary care visits that varied | Primary care, Catalonia, Spain | 2,824,185 | Although non-face-to-face visits increased (+267%), this did not counterbalance the decreased number of face-to-face visits (-47%) and net decrease was 1.36%  There was an increase in codes related to COVID-19 and economic/housing, while most others decreased  Visits for obesity declined 48.58%, bodily injuries 33.70%; chronic pathologies also declined: arterial hypertension 32.7% and diabetes 21.13% |
| Van der Velden, 2021 [75]  Jan-Feb 2020 (PPAS1) and Mar-May 2020 (PPAS2) | Primary care for respiratory infections | Prospective point prevalence audit survey (PPAS) before and early in COVID-19 pandemic; 16 European countries | Patients with respiratory tract infection in primary care networks in 16 selected high, upper-middle, and lower-middle income European countries (1-10 per country) | 4376 consultations in PPAS1 and 3063 in PPAS2.  This was 221-381 per practice in PPAS1 and 114-238 per practice in PPAS2 | 1304 patients (42.6%) suspected of COVID-19 in PPAS2; ranged from 4% in Georgia to 84% in Spain  Many countries were able to rapidly switch from face-to-face consultations to telephone/video consultations early in the pandemic: clinic 38.3%, telephone 50.9%, video/Skype 8.3%, home 2.5%.  Belgium, Denmark, Ireland, Moldova, Netherlands, Romania, and UK had over 70% of consultations by telephone/video.  Antibiotic prescribing decreased (31.6% pre-pandemic, 17.6% early pandemic, 9.3% in patients suspected of COVID-19) overall, but not in Greece, Poland, Romania, and UK)  Hospital referral was 3% pre-pandemic and 8% in early pandemic. During pandemic, hospital referrals were made for 10% in-person and 6.8% telephone/video; for COVID-19 suspicion referral was 21.2% and 12.7%  Antibiotics prescribed in 23.0% face-to-face, 13.9% telephone/video; in cases with COVID-19 suspected prescribing was lower (11.6% and 8.2%) |
| Brown, 2021 [76]  April 2020 and April 2019 | Impact of COVID-19 on Pediatric Primary Care Visits | Retrospective chart review | Pediatric (age 0-18 yr) primary care at 4 large academic institutions in 2 states (North and South Carolina) | 120,230 visits | Well and acute visits were significantly decreased in 2020 (42,670) compared to 2019 (77,560); 6616 of these visits in 2020 were telehealth visits  Visits regarding chronic conditions, pediatric primary care (well visits and chronic care follow-up) were also significantly fewer in 2020  Patients attending a visit in 2020 were more likely to be Black or Hispanic, younger, attending an acute visit, or had private insurance |
| Murphy, 2021 [77]  Apr-Jul 2020 and Apr-Jul 2019 | Implementation of remote consulting due to COVID-19 | Mixed-methods study  Longitudinal observational quantitative analysis for comparison of volume and type of consultation; qualitative data from longitudinal interviews | 21 general practices in Bristol, North Somerset and South Gloucestershire (UK) | 350,966 registered patients | Rapid change to 90% remote GP consulting (89% telephone, 1% video) in April 2020, compared to 31% by telephone (no video) in April 2019.  Consultation rates reduced by 11% for doctors and 17% for nurses/paramedics in 2020 compared to 2019.  Telephone was used in most cases and was considered sufficient for many patient problems; video consulting was used infrequently and was less essential as lockdown eased  SMS-messaging increased more than 3-fold |
| Sigurdsson, 2020 [78]  Mar-Apr 2020 and Mar-Apr 2019 or 2018 | Change in primary healthcare in Iceland due to COVID-19 pandemic | Data were extracted from the medical records database | Primary healthcare, Reykjavik, Iceland | 233,000 people | There were 35% more daytime consultations in 2020 compared to 2018 and 2019; office visits decreased 41%, but teleconsultations increased by 69% and web-based consultations by 213%  Out-of-hours telephone consultations increased 156%; office consultations decreased 46%  Maternity and well-childcare decreased only by 4% (p=0.003).  Laboratory tests decreased by 27%  The number of drug prescriptions increased by 10.3%; those from telephone and web-based consultations increased 55.6%  No changes were observed in total antibiotics prescriptions; telephone/web increased 136.4% and in-office decreased 47.1% |
| Mohamed Ibrahim, 2021 [79]  March-Jul 2020 | Telepharmacy during COVID-19 | Prospective observational study; disguised direct observation  Multivariable logistic regression was used as a tool to predict factors associated with effective telepharmacy services in improving dispensing safety and increasing access of patients to pharmaceutical care | People with probable or confirmed COVID-19  52 community pharmacies across the United Arab Emirates; 26 with and 26 without telepharmacy (videoconferencing, home delivery, medication review, patient counselling) | 7,445 with telepharmacy and 1,246 control | 6,371 probable and 1,074 cases of COVID-19 in pharmacies with telepharmacy; 1213 probable and 33 confirmed cases of COVID-19 in control group  COVID-19 related recommendations: 63,714 vs. 15, 539  Dispensing errors: 15.8% vs. 19.4% |

Abbreviations: aOR, adjusted odds ratio; URI, upper respiratory infection; GP, general practitioner; ED, emergency department

**Table S6. Primary studies on virtual care versus in-person care of minor infections**

| **Source** | **Topic** | **Type of Study** | **Population** | **Number of patients** | **Results and conclusions** |
| --- | --- | --- | --- | --- | --- |
| **Antibiotics: respiratory, urinary, various, ophthalmologic** | | | | | |
| Penza, 2021 [80]  May 2016 – May 2017 | Acute sinusitis; encounters with nurse or advanced practice providers (APP) only (not physicians) | Retrospective chart review, comparison of e-visit (asynchronous text reviewed and responded to by APP), retail health clinic (Mayo Clinic Express Care by same APP), or telephone-based nurse protocol.  Outcomes of antibiotic prescribing and follow-up | Primary care patients aged 18-75 yr, clinical encounter for acute sinusitis at Mayo Clinic Rochester  Excluded primary care appointments | 383 e-visits (text); 968 face-to-face; 2,084 telephone protocol encounters.  Reviewed random sample of 150 of each type | Note: e-visits were asynchronous and outside scope of current review; telephone care was by a nurse following a strict protocol, while in-person care was by APP  Initial antibiotic treatment was 56% e-visit, 61% telephone, 72% face-to-face, p=0.01  Subsequent follow-up rates 18% e-visits, 35% telephone, 14% face-to-face  Many providers at the face-to-face and e-visit encounters instructed their patients to use a guideline-recommended watchful waiting approach, which was not an option for the telephone protocol. This may have led to a lower number of patients starting a prescribed course of antibiotics compared to the number of prescriptions written |
| Murray, 2020 [81]  Aug 2016 – May 2017 | Antibiotic prescribing for urinary symptoms and urinary tract infection | Retrospective chart review, comparison of e-visit (asynchronous text reviewed and responded to by APP), retail health clinic (Mayo Clinic Express Care by same APP), or telephone-based nurse protocol.  Outcomes of antibiotic prescribing and follow-up | Female patients of Mayo Clinic Rochester primary care, age 18-65 yr, urinary symptoms, no urinary complaints in prior 30 days  Excludes telephone patients for whom triage indicated they should have an in-person primary care appointment, or the nurse consulted an APP or physician | 1,673 telephone protocol; 779 face-face; 538 e-visits  Random selection of 150 patients from each type of encounter | Note: e-visits were asynchronous and outside scope of current review; telephone care was by a nurse following a strict protocol, while in-person care was by APP  Dipstick testing and urine cultures at initial counter more likely at face-to-face visits  Similar antibiotic prescribing rates in all groups: 81% e‑visit, 81% telephone, 83% face-to-face  Follow-up was recommended more often for telephone (19%) and e-visits (19%) than in-person (8%), but actual follow-up within 30 days were not significantly different (32%, 31%, 26%) |
| Shi, 2018 [82]  2015-2016 | Acute respiratory infections, antibiotic management | Claims data for 2015–16 from a large national commercial insurer, telemedicine visits matched to primary care and urgent care visits | Adults aged 18-64 with acute respiratory infection diagnoses, and with pharmaceutical coverage | 38,839 telemedicine (audio or audiovisual); 942,613 primary care; 186,016 urgent care | Age category, sex, presence or absence of chronic conditions, state, urbanicity of ZIP code, high deductible health plan status, and diagnosis category were used to match visits in order to address selection biases.  Clinically similar antibiotic use, broad-spectrum antibiotic use, and guideline-concordant antibiotic management in matched analyses  Less appropriate streptococcal testing and a higher frequency of follow-up visits in the telemedicine group |
| Ray, 2019 [83]  2015-2016 | Pediatric antibiotic prescribing for acute respiratory infections | Retrospective cohort study using 2015–2016 claims data from a large national commercial health plan  Coarsened-exact matching matched direct-to-consumer (DTC) telemedicine visits to many matched urgent care visits and PCP visits and then weighted each stratum or matched set. | Children aged 0 to 17 yr with pharmaceutical coverage | 4604 DTC; 38,408 urgent care, 485,201 PCP in matched sample | Matched groups by, sex, chronic medical complexity, state, rurality, health plan type, and acute respiratory infection diagnosis category  In unmatched sample:  At DTC telemedicine visits, children were more likely to receive a diagnosis of viral acute respiratory infection (68% vs. 54% urgent care; 59% PCP visits) or sinusitis (18% vs. 8% urgent care; 10% PCP visits) and less likely to receive a diagnosis of otitis media (8% vs. 26% urgent care; 23% PCP).  In matched sample:  Antibiotic prescribing: 52% telemedicine, 42% urgent care, 31% PCP, p<0.001 for telemedicine vs. other groups  Guideline concordant antibiotic use: 59% telemedicine, 67% urgent care, 78% PCP, p<0.001 for telemedicine vs. other groups  Streptococcal testing in patients diagnosed with streptococcal pharyngitis: 4% telemedicine, 75% urgent care, 68% PCP, p<0.001 for telemedicine vs. other groups  DTC telemedicine had higher subsequent visits within 2 days than other settings (5% vs. 2% urgent care; 1% PCP; p<0.001 for both). |
| Ray, 2021 [84]  Apr-Sep 2020; same months in 2018 and 2019 for comparisons | Antibiotics for pediatric acute respiratory tract infections | Retrospective, electronic health record data from pediatric primary care network  Telehealth due to regulatory and payment changes associated with COVID-19 | Visits for acute respiratory tract infection | 8332 visits in 2020 (3003 telemedicine, 36%) | Guideline concordant management: 92.5% telemedicine, 90.7% in-person, p=0.004  Antibiotic prescriptions in April 2020: 59% telemedicine and 71% in person, p<0.001  Antibiotic prescriptions in Sept 2020: 19% telemedicine and 46% in-person, p<0.001  Guideline-concordant management of sinusitis and viral acute respiratory infection 97% in September compared to 88% in April |
| Ewen, 2015 [85]  2006-2010 | Antibiotic prescribing by telephone in primary care | Retrospective cohort, chart review  Practices affiliated with a large US healthcare system (Christiana Care Health System, Newark, Delaware) | Patients who visited teaching and non-teaching internal medicine, pediatrics, family practice, and obstetrics/ gynecology practices for any reason | 114,610; equivalent to 219,282 patient-years.  Rationale for telephone vs. office visit not mentioned | Telephone-based prescribing increased from 2.2 to 4.2 per 100 patient-years during study; of these, 5.7% were due to administrative reasons or drug review  Office-based prescribing increased from 21.4 to 26.1 per 100 patient-years  12.4% of all antibiotic prescriptions were prescribed by telephone. 39% of patients who were prescribed antibiotics by telephone had an office visit within 7 days prior to the prescription |
| **Ophthalmology** | | | | | |
| Frost, 2021 [86]  2017-2020 | Ophthalmic antibiotics for acute infections conjunctivitis in children | Retrospective analysis, multivariable logistic regression modeling. Telephone vs. in-person visits | Children with acute infectious conjunctivitis, Denver Health and Hospital Authority clinics | 5,283 | Ophthalmic antibiotics in 72.7%; this was 67.8% prior to COVID-19 pandemic and 81.9% during the pandemic  Compared to pediatric clinics, antibiotic prescription was lower in family medicine (aOR=0.69) or optometry/ophthalmology clinics (aOR=0.06) and higher in telephone evaluation (aOR=5.43) |
| Penza, 2020 [87]  2016-2017 | Pediatric conjunctivitis without exam |  | Pediatric patients evaluated for conjunctivitis. Mayo Clinic Rochester Employee and Community Health, Minnesota | 202 in-person retail clinic visits (nurse practitioner), 202 nurse telephone calls (nurse protocol and/or triage) | Patients younger for telephone visits (mean 5.9 yr) vs. face-to-face (mean 7.1 yr), p=0.004  Treatment with antibiotics at initial encounter: 41.6% telephone vs. 19.8% face-to-face, p<0.0001  Recommended follow-up (mostly in-person with primary care physician): 38.6% vs. 1.0%, p<0.0001; actual follow-up 45.5% vs. 7.4%, p<0.0001  Follow-up was rapid (mean 0.15 days vs. 3.6 days) and mostly those who were not initially treated  Treatment at follow-up: 54% vs. 47%, p=0.66  Conclusion: higher antibiotic prescribing and more in-person follow-up visits with telephone consultation; may signify poorly designed protocol sets/triage questions |

Abbreviation: APP, advanced practice providers; DTC, direct-to-consumer; PCP, primary care physician; aOR, adjusted odds ratio

**Table S7. Systematic reviews on virtual care versus in-person care of minor infections**

| **Source** | **Topic** | **Patient population** | **Number of included RCTs** | **Number of included non-RCTs** | **Review conclusions** |
| --- | --- | --- | --- | --- | --- |
| **Antibiotics: Sinusitis or urinary** | | | | | |
| Bakhit, 2021 [88] | Antibiotics prescribing | Adult and pediatric patients with a history of a community-acquired acute infection (respiratory, urinary, or skin and soft tissue) receiving synchronous telehealth consultations | 1 | 10 | The impact of telehealth on prescribing varies, with more increases than reduction  There is insufficient evidence to draw strong conclusions; higher quality research is urgently needed |
| Han, 2020 [89] | Antibiotics prescribing | Children and adults receiving remote consultations | 1 | 11 | There is insufficient evidence to confidently conclude that remote consulting has a significant impact on antibiotic prescribing in primary care  Studies indicating higher prescribing rates in remote consultations than in face-to-face consultations are a concern |

Abbreviations: RCT, randomized control trial

**Table S8. Primary studies of virtual care versus in-person care in COVID-19 management**

| **Source** | **Topic** | **Type of Study** | **Population** | **Number of patients** | **Results and conclusions** |
| --- | --- | --- | --- | --- | --- |
| **COVID-19** | | | | | |
| Phillips, 2021 [90]  Mar 23 – May 23 2020 | Assessing and treating patients with symptoms consistent with COVID-19 | Retrospective cross-sectional study: patients with respiratory concerns possibly related to COVID-19 referred to Respiratory Assessment Centre and triaged by telephone to in-person or telehealth visit | Symptoms consistent with COVID-19; large academic primary-care-run Respiratory Assessment Centre | 1305 initial visits: 741 (56.8%) traditional and 564 (43.2%) telehealth | Type of visit determined by clinical judgement and not randomized  33% of visit diagnoses were viral URI and viral URI with cough  Follow-up within 14 days occurred for 25.9% of telehealth visits vs. 21.7% of the office visits; included 12.2% vs. 8.9% of initial visits resulting in subsequent office visit (p=0.033), 11.0% vs. 9.7% with subsequent telehealth visit, 5.0% vs. 3.9% with ED visit, and 2.0% vs. 2.8% hospital admissions |
| Tan, 2021 [91] | COVID-19 | Retrospective health record review, community-based academic family medicine practice, University of California, Los Angeles  March 3-31, 2020 | Patients with potential COVID-19 symptoms in primary care  Due to COVID-19 restrictions, patients urged to have telehealth (smartphone) or telephone visit, but in-person still allowed | 202 patients: 89 telehealth (smartphone), 55 telephone, 52 in-person visit  Definition of telehealth is not given, no mention of whether it is video or text-based | Mean number of interactions with the office varied by type of initial appointment: 6.1 telehealth, 5.2 telephone, 4.5 in person (no significant difference); 9%, 12.7%, and 19.2% had a subsequent in-person or emergency department visit  Older patients and those with subjective fevers or shortness of breath had more interactions  In-person visits had more testing for influenza, more antibiotics prescribed, less COVID-19 testing, more fever, less shortness of breath  Children were more likely to have in-person visits |
| Riese, 2021 [92]  Apr-Aug 2020 | Possible COVID-19 in pediatric primary care | primary care clinic at Hasbro Children’s Hospital in Rhode Island, staffed by general pediatric faculty and residents | Children (up to 18 yr) with symptoms suggesting possible COVID-19 | 476 (383 in-person, 93 telehealth) | Most common symptoms were fever (n=205, 43%), congestion/rhinorrhea (n=201, 42%), and cough (n=177, 37%).  Fever (27% vs. 47%, p<0.001), sore throat (15% vs. 30%), nausea/vomiting (11% vs. 20%) were less prevalent in telehealth. Test ordered for 63% telehealth vs. 79% in-person; positivity rate 14% vs. 7% |
| Irving, 2021 [93]  April 22, 2020 to June 30, 2020 | Drop-box doorstep assessment for COVID-19 to complement telephone/video to reduce in-person assessments | Retrospective case review of patients  Drop-box of equipment to test physiological parameters such as temperature, pulse, blood pressure, oxygen saturation; sounds by digital stethoscope (phase III only) to replace in-person assessment | Adults with suspected COVID-19 after telephone/video assessment with GP clinician in St. Helens (UK) area | 61 doorstep and 138 hot hub (in-person) assessments (staffed by GPs and advanced nurse practitioners) | Two forms of doorstep assessment: (1) by the patient themselves with support of a visiting healthcare assistant if needed or (2) diagnostic review by a GP via video link with full vital signs  Prior to hub start, all patients had face-to-face assessment; decreased to 73%, 72%, 0% in phase I, II, and III (with stethoscope) of doorstep assessment  8% hospital admissions from door-step assessment vs. 5% seen in person at hub |

Abbreviations: aOR, adjusted odds ratio; URI, upper respiratory infection; GP, general practitioner; ED, emergency department

**Table S9. Primary studies on virtual care versus in-person care in chronic disease management**

| **Source** | **Topic** | **Type of Study** | **Population** | **Number of patients** | **Results and conclusions** |
| --- | --- | --- | --- | --- | --- |
| **Asthma** | | | | | |
| Gruffydd-Jones, 2005 [94]  Dec 2002- Mar 2003 | Targeted routine asthma care in general practice using telephone triage | RCT: routine asthma care by asthma nurse either by telephone triage (phone call every 6 months) or in the surgery (visits every 6 months).  For triage, if deemed “high-risk” patient was seen in the clinic until stable | Age 17-70 yr, on asthma list of a semi-rural practice in Wiltshire, England  Stratified according to severe or mild/moderate asthma | 194; of these 11 were considered to have severe asthma | 35% per cent more patients (n = 84 vs. n = 62) received more than one consultation in the telephone group. 15 in-person and 7 telephone group did not attend baseline visit and were excluded  Asthma control parameters were measured by the asthma control questionnaire (ACQ). There were no significant changes in asthma control between the clinic and telephone groups (mean change ACQ = ‑0.11 vs. -0.18). Mean costs per patient per year for the British Nation Health System were £334 for the clinic group and £210 for the telephone group (p=0.071). |
| Pinnock, 2007 [95] | Telephone option for routine asthma review | RCT; phase IV controlled before-and-after implementation study  Patients in 2 centres were randomized to structured recall with a telephone-option for reviews vs. structured recall with face-to-face-only reviews; patients in one centre received usual care | All patients on asthma register (diagnosed with asthma and prescribed medication) in Whitstable Medical Practice, a large general practice in the UK.  Asthma care was by asthma-trained nurses | 1,809  Telephone group had choice of face-to-face or telephone review | A routine asthma review was achieved for 397/598 (66.4%) patients in the telephone-option group, 352/654 (53.8%) in the face-to-face only review group, and 282/557 (50.6%) in the usual care group. The risk difference was 12.6%, p<0.001 between the telephone-option and face-to-face only groups.  There was no difference in morbidity. Enablement and confidence in asthma management were higher in the telephone group  Cost was less for telephone than face-to-face (£10.03 vs. £12.74, p<0.001); usual-care costs were £11.85 per review |
| Pinnock, 2003 [96] | Routine telephone review of asthma | Pragmatic, RCT: telephone vs. face-to-face consultation with asthma nurse | Adults with asthma who did not have a review in the previous 11 month from 4 general practices in England | 278 (137 telephone, 141 face-to-face) | 74% in the telephone group and 48% in face-to-face group were reviewed were reviewed (p<0.001, number needed to treat=3.8).  There was no difference in Juniper Quality of Life score (risk difference ‑0.07) nor satisfaction (risk difference ‑0.07) at three months post-randomization. The average duration of telephone consultations were 10 minutes less than face-to-face reviews (p<0.001). |
| **Diabetes** | | | | | |
| Al Harthi, 2021 [97]  Before pandemic in 2019 to end of 2020 | Impact of COVID-19 on diabetes care | Retrospective, within-patient database study, at least 2 visits pre-pandemic (2019) and followed to end of 2020 | Primary care, age ≥18 yr with diabetes mellitus, Muscat Governorate (Oman) | 937 (793 with HbA1c results)  After start of pandemic, 57.4% had face-to-face alone, 32.4% had combined face to face and telephone consultation, and 10% had telephone consultation alone | Mean difference (before and after) in HbA1c (%) was 0.2±1.4 (95% CI: 0.1 to 0.3), p=0.002  Multivariate analysis of change in HbA1c according to mode of consultations after pandemic started: -0.3 telephone; -0.5 face-to-face; -0.5 both.  Univariate but not multivariate analysis found more increase in HbA1c with telephone consultation and evidence is inconclusive. Patients only receiving face to face consultations did not have a better outcome compared to patients receiving various modes of consultation. |
| Lu, 2021 [98]  Jan 2018 – Dec 2019 | Diabetes | Before and after, propensity-scored matched cohort.  Hub-and-spoke model with hub team of remote primary care providers working with local staff at smaller spoke clinics.  Patient at spoke communicated by videoconference to hub | Patients with diabetes at Veterans Affairs primary care clinics when Virtual Integrated Multisite Patient Aligned Care Teams (V-IMPACT) program was implemented. | 9010 patients, either in V-IMPACT or usual in-person care | V-IMPACT: the change in mean HbA1C was −0.055% (95% CI −0.088 to −0.022%); usual care had a −0.047% (95% CI − 0.080 to − 0.014%).  V-IMPACT was not associated with a significant difference in the proportion with controlled blood pressure  Diabetes care by a longitudinal virtual primary care model was similar if not better than in-person care |
| Turner, 2020 [99]  2013-2017 | Uncontrolled diabetes | Retrospective cohort study  Patients offered community health worker care management in face-to-face meetings in clinic, by telephone, or both.  Urban primary care practices in Texas serving primarily Hispanic patients | Low-income Hispanic primary care patients with HbA1c ≥9%; age 18-75 yr; diagnosis of diabetes at 1 inpatient or 2 outpatient encounters | 827 enrolled, 523 with at least 6 months follow-up are reported  Initial 15-30 min meetings by community health worker and attempt to follow-up at least once | Type of community health worker care management [CM] delivered over 6 months was classified as: CM1—no visits and one or more telephone calls (n=51); CM2—one visit but no calls (n=192); CM3—one visit and one or more calls (n=44); and CM4—two or more visits with or without calls (n=236)  CM2 and CM4 were associated with a shorter time to diabetes control compared to CM1 or CM3, log-rank p=0.053  Face-to-face had significantly greater improvement, even after adjusting for variables involving diverse demographics, treatment, and healthcare |
| **Skin ulcers** | | | | | |
| Wickstrom, 2021, 2018 [100, 101]  Oct 2014 – Sept 2016 | Skin ulcers, hard to heal (chronic) | Video consultation by GP at Blekinge Wound Healing Centre, a primary care centre covering the whole of Blekinge county (Sweden) vs. data from a registry of in-person assessment at any clinic using Registry of Ulcer Treatment management | Swedish patients, age >18 yr, with hard-to-heal ulcers (not healed within 4-6 weeks) | 100 patients diagnosed by video consultation; 1888 control patients with in-person assessment (subgroup of 100 selected for wait time analysis) | Patients are treated according to a structured wound management based on a Swedish national quality registry, the Registry of Ulcer Treatment  Diagnosis of pain by visual analogue scale: no significance between video and in-person (90% vs. 86%, χ^2^ p=0.233; pain score at diagnosis 5.1 vs. 5.7, and severe pain was present in 62% vs. 71% of patients  Etiology of ulcers was significantly different  Analgesics prescribed in 84% vs. 68% of patients (χ^2^ p=0.044)  Median healing time 59 days vs. 82 days (p<0.001); median wait time was 25 days vs. 32 days  Living in remote areas and being unable to reach the healthcare centre for assessment could account for increased healing time in the video group  Comparison of the clinical trial centre to typical centres is also problematic |
| **Opioids** | | | | | |
| Eibl, 2017 [102]  2011-2012 | Telemedicine-delivered opioid agonist therapy | Non-randomized cohort comparison study using an administrative database  Stratified by in-person (<25% telemedicine), mixed, or telemedicine (>75% telemedicine | Patients receiving opioid agonist therapy across 58 clinics in Ontario | 3733 patients | Retention rate at 1 yr: 50% telemedicine vs. 39% in-person, aOR= 1.27, p<0.001).  Mixed group had retention rate of 47% at 1 yr, which was higher than in-person (aOR=1.26; p=0.001)  Telemedicine has potential to be an effective alternative to in-person opioid agonist therapy and a more accessible treatment option for rural, remote, and urban populations. |
| **Pharmacists** | | | | | |
| Rivera, 2020 [103]  2011-2017 | Comprehensive medication reviews | Retrospective | Community pharmacies in Western Massachusetts, USA in a national chain | 297; 56.5% over telephone | No significant differences in clinical and demographic characteristics or type of medications, problems, or interventions between groups (phone vs. face-to-face)  Encounters by telephone were more often documented (42% vs. 28%, p<0.05), and plan documented (40% vs. 27%, p<0.05). Discussion notes mostly documented (98% vs. 97%), as were pharmacist recommendations (95% vs. 92%) |

Abbreviations: RCT, randomized controlled trial; HbA1c, hemoglobin A1c; CI, confidence interval; GP, general practitioner; aOR, adjusted odds ratio

**Table S10. S**ystematic reviews on virtual care versus in-person care in chronic disease management

| **Source** | **Topic** | **Patient population** | **Number of included RCTs** | **Number of included non-RCTs** | **Review conclusions** |
| --- | --- | --- | --- | --- | --- |
| **Opioid** | | | | | |
| Ho 2018a [104]  CADTH rapid response report | Telehealth-delivered opioid agonist therapy | Adolescents (ages 12 to 17) and adults (≥18) with opioid use disorder | 0 | 2 | Limited evidence, based on one non-randomized study, showed that telehealth-delivered opioid agonist therapy seemed to be associated with a higher likelihood of uninterrupted treatment retention at 1 yr than in-person opioid agonist therapy |
| **Dementia** | | | | | |
| Barth, 2018 [105] | Scoping review: diagnosis of cognitive decline and dementia | Videoconference vs. in-person Interventions targeted at rural living elderly to screen and diagnose cognitive decline and dementia | 1 | 11 | Of the 12 studies, 11 showed high similar test scores and significant correlations between videoconference and face-to-face examination |

Abbreviation: CADTH, Canadian agency of drug and technologies in health; RCT, randomized control trial

Table S11. Primary studies of virtual care versus in-person care in medical abortion

| **Source** | **Topic** | **Type of Study** | **Population** | **Number of patients** | **Results and conclusions** |
| --- | --- | --- | --- | --- | --- |
| **Abortion** | | | | | |
| Grossman, 2017 [106]  Jul 2008 – Jun 2015 | Medical abortion by telemedicine | Retrospective cohort non-inferiority study, telemedicine vs. in-person patients.  Data on adverse events came from required reporting forms submitted to the mifepristone distributor and survey of emergency departments | Pregnant women using clinic system in Iowa.  In-person medical history, hemoglobin, physical exam, ultrasound and then discussion with physician either in-person or by videoconference | 8,765 telemedicine and 10,405 in-person medical abortions were performed  Did not include cases of ongoing intrauterine pregnancy as adverse event because this is a known possible outcome of medical abortion | 49 clinically significant adverse events: 0.18% telemedicine vs. 0.32% in-person (p=0.07)  None of the 42 responses from emergency departments (35% response rate) reported treating a woman with an adverse event after medical abortion |
| Grossman, 2013 [107]  2006-2010 | Medical abortion by telemedicine | Service patterns 2 yr before and 2 yr after introduction of telemedicine based on vital statistics records from state of Iowa | Pregnant women using a Planned Parenthood affiliate in Iowa  Ultrasound by technician for all women; either videoconference or in-person physician discussion and dispensing | 17,956 | After the introduction of telemedicine, the abortion rate in Iowa decreased while the proportion of abortion in medical clinics increased (46% to 54%).  Clinic patients had increased odds of obtaining both medical abortion and abortion before 13 weeks of gestation after adjustment for other factors.  The likelihood of obtaining an abortion by women living farther than 50 miles increased  Telemedicine may improve access to medical abortions, improve access for women living in remote areas, and reduce the likelihood of second-trimester abortions. |
| Grossman, 2011 [108]  Nov 2008-Oct 2009 | Medical abortion by telemedicine | Prospective cohort study; medical abortion by telemedicine or face-to-face physician visits | Pregnant women using a Planned Parenthood affiliate in Iowa  Ultrasound by technician for all women; either videoconference or in-person physician discussion and dispensing | 578; follow-up data for 223 telemedicine and 226 face-to-face patients | Successful abortion: 99% telemedicine patients vs. 97% face-to-face patients  In multivariable analysis, telemedicine patients had a higher odds of saying they would recommend the service to a friend compared to face-to-face-patients (OR=1.72); 25% of telemedicine patients reported they would have preferred to be in the same room with the doctor  There was no significant difference in adverse events |
| Grossman, 2018 [109]  Jan 2012-Mar 2013 | Care after medical abortion | Telephone vs. in person follow-up after medical abortion, according to patient choice | Pregnant women considering medical abortion with misoprostol in Peru | 253 | 3% miscarried, 9% decided to continue pregnancy; of those who had abortion, follow-up was 34% telephone, 34% telephone + in-person, 12% in-person only, 20% none  Satisfaction was the same for telephone or in-person  Rates of adverse events were similar across women with in-person and telephone follow-up |
| Cameron, 2012 [110]  May 2010-Feb 2011 | Urine pregnancy testing after early medical abortion | Telephone screen for pregnancy symptoms, bleeding, and self-administered urine pregnancy test vs. in-person follow-up including ultrasound | Royal Infirmary of Edinburgh, Scotland | 616; 77% preferred telephone follow-up (87% of these could be contacted) | 60 (15%) with telephone follow-up screened ‘positive’ (28 positive LSUP tests, 19 invalid tests, 3 pregnancy symptoms, 10 scant bleeding). Of these 60, three had ongoing pregnancies, 5 had incomplete abortions, and one was falsely screened ‘negative’.  For telephone follow-up: sensitivity 75%, specificity 86%, negative predictive value 99.7%, positive predictive value 5%.  100% of women surveyed would recommend telephone follow-up to a friend  21% (30/140) of those who chose clinic follow-up with a routine ultrasound did not attend. Of these 30 nonattenders, one ongoing pregnancy resulted in a live birth, according to the regional hospital database. There was 15% loss to follow-up in the telephone group and 21% in the clinic group (p = 0.09).  Some women may have had difficulty performing the urine test, as LSUP was negative in 85% of the at-home group and 96% of the clinic group |

Abbreviations: OR, odds ratio; LSUP, low-sensitivity urine pregnancy test; RCT, randomized controlled trial; VHA , Veterans Health Administration; IRR, incidence rate ratio; aOR, adjusted odds ratio; ED, emergency department

**Table S12.** Primary studies of virtual care versus in-person care in rehabilitation

| **Source** | **Topic** | **Type of Study** | **Population** | **Number of patients** | **Results and conclusions** |
| --- | --- | --- | --- | --- | --- |
| **Cardiac rehabilitation** | | | | | |
| Maddison, 2019 [111]  Aug 2014-Jan 2016 | Remotely monitored exercise-based cardiac telerehabilitation  ACTRN12614000843651 | RCT, inferiority trial  12 weeks of telerehabilitation (real-time remote monitoring and coaching) vs. centre-based rehabilitation at clinic, New Zealand | Adults with coronary heart disease (atherosclerosis, angina pectoris, myocardial infarction, coronary revascularization) | 162 participants | Maximal oxygen consumption (VO_2_ max) improved by similar amount in both groups (remote 30.5 mL/kg/min at week vs. 27.2 at baseline, centre 29.4 at week 12 vs. 27.7 at baseline); remote rehabilitation was noninferior  Remote costs per person were lower: 4920 NZD vs. 9535 NZD  More adverse events in telerehabilitation group during treatment, including soft tissue injuries and a broken ankle; others were unrelated or possibly related to treatments |
| Scalvini, 2013 [112]  2006-2010 | Home vs. hospital cardiac rehabilitation | Quasi-experimental, not randomized  4 weeks of home-based cardiac rehabilitation (telemonitored by nurse and physical therapist by videoconference) vs. in-hospital  Italy | Patients at low to medium risk of early mortality following cardiac surgery; self-selected for home rehabilitation while authors selected patients for comparison group | 100 adult patients in each group | Both groups improved from baseline in 6-minute walk test  Study showed that home-based cardiac rehabilitation is feasible and yields similar outcomes for the majority of patients |
| **Rehabilitation after arthroplasty** | | | | | |
| Moffet, 2015 [113] | Rehabilitation after total knee arthroplasty | Noninferiority RCT  Physiotherapist (physical therapist) guided in home telerehabilitation using videoconference vs. face-to-face at home | Patients discharged after total knee arthroplasty; surgery was in eight hospitals in three Quebec regions | 205 | Similar demographic and clinic characteristics at baseline  Baseline adjusted values at last follow-up were similar and within the predetermined zone of noninferiority for Western Ontario and McMaster Universities Osteoarthritis Index (WOMAC) for total score for pain, stiffness, function  Secondary outcomes of knee injury and osteoarthritis outcome score, functional and strength tests, and knee range of motion had similar results |
| Piqueras, 2013 [114]  Nov 2008 – Dec 2010 | Telerehabilitation after total knee arthroplasty  Barcelona, Spain | RCT; interactive virtual telerehabilitation system (1 hour/day for 5 days with therapist, then 5 sessions at home) vs. out-patient physical therapy (1 hour/day for 10 days) | Patients who had total knee arthroplasty | 142 | Compared at the end of rehabilitation (2 weeks) and 3 months. Both groups improved to similar extent; virtual group had better muscle quadriceps strength at 2 weeks and 3 months |
| Kuether, 2019 [115] | Telerehabilitation for Total Hip and Knee Arthroplasty | Retrospective pilot study: compared first 40 telerehabilitation patients after a primary total hip or knee arthroplasty with a historical cohort or literature referenced values | Patients scheduled for unilateral primary total hip or total knee arthroplasty who were able to ambulate without an assistive device at baseline | 40 telerehabilitation; 614 historical controls with traditional home or outpatient physical therapy | There was no increase in readmissions, emergency department visits, or closed knee manipulations at 90 days in the telerehabilitation group; accuracy of telerehabilitation exercises was found to be 92%  There were improvements in patient-reported outcome scores in telerehabilitation patients comparable with traditional therapy, in addition to extremely high patient satisfaction scores  Telerehabilitation following primary total hip or knee arthroplasty was found to be feasible with comparable clinical quality and high patient satisfaction |
| **Physiotherapy, movement, activity** | | | | | |
| Nicola, 2018 [116] | Movement assessment in children by physiotherapists or physiotherapy students | Both in-person and remote (video) assessment in same children (test-retest in randomized order). Used Movement Assessment Battery for Children – 2^nd^ Edition | Children of general school population (age 5-11 yr and typically developing) in Queensland Australia whose parents consented | 59 children | 5 students were placed in different categories (switched between no movement difficulty and should be monitored categories); 2 students were identified as having significant movement difficulty by both methods |
| Ewald, 2018 [117] | Physical activity coaching | 3-arm RCT. Face-to-face (5 visits) vs. telephone coaching (1 in person plus 4 by telephone) over 12 weeks by exercise physiologist vs. control (printed pamphlet) to improve activity measured by step counts per week | Sedentary patients seen in general practice, Australia | 203 randomized  Average overall consultation time 165 min face-to-face group and 134 min telephone group | The face-to-face and telephone groups were found to be more active than the control group by 1002 steps per day (95% CI=244 to 1759) at a cost of AUD $245 per person; no change was observed in quality of life and therefore utility values  Step counts baseline, 3 months, and 12 months: control 4415 and 5386 and 4736 steps/day; face-to-face 4549 and 6172 and 5346 steps/day; telephone 4309 and 5949 and 6289 steps/day  There was wide variation and differences were not significant; all 3 groups increased at 3 months while telephone increased further at 12 months and other groups decreased, suggesting there was no real effect of either intervention  No change in quality of life observed |
| Cottrell, 2018 [118] | Advanced-practice physiotherapy assessment | Repeated measures study design; 2 consecutive assessments (in-person, telehealth) within a single clinic session) in randomized order | Patients referred to the Neurosurgical & Orthopaedic Physiotherapy Screening Clinic (Queensland, Australia) for assessment of their chronic lumbar spine, knee, or shoulder condition | 42 | There was substantial agreement (83.3%; 35/42 cases) between in-person and telehealth (videoconference using iPad) assessments for recommended management pathway and for clinical diagnosis (same or similar diagnosis) |

Abbreviations: CI, confidence interval

**Table S13. Systematic reviews of virtual care versus in-person care in rehabilitation**

| **Source** | **Topic** | **Patient population** | **Number of included RCTs** | **Number of included non-RCTs** | **Review conclusions** |
| --- | --- | --- | --- | --- | --- |
| **Cardiac** | | | | | |
| Inglis, 2015 [119]  Cochrane Library | Structured telephone support for heart failure | Adults with a definitive diagnosis of heart failure | 25 | 0 | For people with heart failure, structured telephone support and non‐invasive home telemonitoring reduce the risk of all‐cause mortality and heart failure‐related hospitalizations and improved health‐related quality of life, heart failure knowledge and self‐care behaviours  Studies found participant satisfaction with most of the interventions |
| Anderson, 2017 [120]  Cochrane Library | Home-based cardiac rehabilitation | Adults with myocardial infarction, angina, heart failure or who had undergone revascularization  Most studies were with lower-risk patients following an acute myocardial infarction or revascularization | 23 | 0 | Home-based cardiac rehabilitation had similar benefit to centre-based programs regarding death, exercise capacity, quality of life in short term  Arms often not equivalent in treatment: most home programs based on walking with some intermittent nurse or exercise specialist telephone support; in-person programs usually used supervised cycle and treadmill exercise  Intensity of the rehabilitation program often different between home and centre-based arms  Many of the included studies do not meet the criteria of the current review |
| Jin, 2019 [121] | Secondary prevention of coronary heart disease | Patients with coronary heart disease  Studies of the effects of telehealth interventions on risk factor modification in patients with coronary heart diseases with at least three months’ follow-up compared with cardiac rehabilitation and/or usual care | 30 | 0 | Telehealth was not significantly associated with a lower all-cause mortality than cardiac rehabilitation and/or usual care (RR=0.60, p=0.42).  Telehealth reduced rehospitalization or cardiac events (RR=0.56, p<0.0001) compared with non-intervention groups |
| **Opioid** | | | | | |
| Ho 2018a [104]  CADTH rapid response report | Telehealth-delivered opioid agonist therapy | Adolescents (ages 12 to 17) and adults (≥18) with opioid use disorder | 0 | 2 | Limited evidence, based on one non-randomized study, showed that telehealth-delivered opioid agonist therapy seemed to be associated with a higher likelihood of uninterrupted treatment retention at 1 yr than in-person opioid agonist therapy |
| **Rehabilitation, physiotherapy** | | | | | |
| Cottrell, 2017 [122] | Telerehabilitation for the treatment of musculoskeletal conditions | Adults (≥18 yr) presenting with any diagnosed primary musculoskeletal condition, including post-operatively for surgical procedures as a result of a primary musculoskeletal condition  Trials had to compare synchronous telerehabilitation to face-to-face or usual care | 8 | 6 | Sub-group analysis found that telerehabilitation in addition to usual care is more than usual care alone; treatment delivered solely via telerehabilitation is equivalent to face-to-face intervention for the improvement of physical function  The improvement of pain was comparable between cohorts |
| Jiang, 2018 [123] | Rehabilitation after total knee arthroplasty | Patients who had total knee arthroplasty  Comparison of telerehabilitation vs. face-to-face rehabilitation | 4 | 0 | Telerehabilitation achieved similar pain relief and better Western Ontario and McMaster Universities Osteoarthritis Index improvement compared with face-to-face rehabilitation  Telerehabilitation treatment resulted in a significantly higher extension range (p<0.00001) and quadriceps strength (p=0.0002) than face-to-face rehabilitation |
| Shukla, 2016 [124] | Telerehabilitation after total knee arthroplasty | Patients who had total knee replacement surgery | 4 | 2 | Patients experienced high levels of satisfaction with the use of telerehabilitation alone  There was no significant difference in change in active knee extension and flexion in the home telerehabilitation group as compared to the control group  The patients in the home telerehabilitation and conventional rehabilitation groups showed similar improvement in physical activity and functional |
| Rawstorn, 2016 [125] | Telehealth exercise-based cardiac rehabilitation | Adults diagnosed with coronary heart disease receiving secondary prevention outpatient telehealth exercise-based cardiac rehabilitation (exCR) | 11 | 0 | Telehealth exCR was as effective as centre-based exCR for improving modifiable cardiovascular risk factors and functional capacity and could enhance exCR utilization by providing additional options for patients who cannot attend centre-based exCR  Telehealth exCR must capitalize on technological advances to provide more comprehensive, responsive, and interactive interventions |
| Mani, 2017 [126] | Validity and reliability of Internet-based physiotherapy assessment for musculoskeletal disorders | Telerehabilitation-based physiotherapy assessment for musculoskeletal conditions | 0 | 11 | Telerehabilitation-based physiotherapy assessment was technically feasible with overall good concurrent validity and excellent reliability, except for lumbar spine posture, orthopedic special tests, neurodynamic tests and scar assessment |
| **Speech and language pathology** | | | | | |
| Canadian Agency for Drugs and Technology in Health, 2015 [127] | Telehealth for speech and language pathology | Children with speech and language impairment or disorders | 2 | 0 | Speech-language pathology treatment, delivered via videoconferencing or an in-person service model, improved children’s speech-language impairments, and there were no significant differences found between these two models |

Abbreviations: RR, relative risk; exCR, exercise-based cardiac rehabilitation

Table S14. Primary studies on virtual versus in-person counselling

| **Source** | **Type of Treatment** | **Type of Study** | **Population** | **Number of Patients** | **Results and Conclusions** |
| --- | --- | --- | --- | --- | --- |
| **Depression** | | | | | |
| Alegria, 2014 [128]  May 2011-Sept 2012  See also Alcantara, 2016 [129] for worry substudy | CBT - Engagement and Counseling for Latinos (ECLA) | RCT; Telephone vs. face-to-face vs. usual care | Low-income Latinos recruited from primary care; Boston (Massachusetts) and San Juan (Puerto Pico) | 257 | Both CBT arms significantly better than usual care and not significantly different from each other; higher treatment initiation with telephone than face-to-face (89.7% vs. 78.8%)  Depression worse at baseline and end in San Juan than Boston, but improvement at both sites |
| Kirkness, 2017 [130]; Byun, 2021 [131]  Seattle (WA) | CBT for post-stroke depression  Living Well with Stroke 2 (LWWS 2) | RCT; telephone vs. in-person vs. usual care (home follow-up visits) for 6 weeks with psychosocial nurse practitioner therapist | Within 4 months of an ischemic or hemorrhagic stroke and with clinical depression; medication if prescribed by primary care physician was allowed | 100 (37 telephone, 35 in-person, 28 usual care) | Follow-up at 8, 21, and 52 weeks after entry to study  Results favoured intervention over usual care (remission 37% vs. 27%, p=0.3; Hamilton Rating Scale for Depression score 39% reduction vs. 33% reduction, p=0.3) but not statistically significant; telephone and in-person were of comparable effectiveness  Secondary analysis found fatigue, sleep disturbance, and wake disturbance decreased over 12 months in intervention groups but not control; only wake disturbance was more than minimal clinically important difference but not statistically significant |
| Mohr, 2012 [132]  Kalapatapu, 2014 [133]; Stiles-Shields, 2014, 2015 [134, 135] | CBT | RCT, noninferiority; telephone vs. face-to-face for 18 sessions  Chicago area  2007-2010 | Major depressive disorder, primary care  Secondary analysis on subset with concurrent problematic alcohol use [133] | 325  Subset with alcohol problem: 50 telephone and 53 face-to-face | Attrition (completion vs. non-completion): 20.9% vs. 32.7%, p=0.02  Improvement in depression in both arms, no significant differences between groups at end of treatment (within inferiority margin)  At 6-month follow-up both groups significantly less depressed than baseline, but the face-to-face group significantly less depressed than telephone group  No significant difference in working alliance scores and the scores did not predict depression at follow-up  Demographic and psychological characteristics identified 85% of treatment responders and non-responders  In patients with problematic alcohol use, telephone and face-to-face groups had similar treatment adherence and efficacy for treatment of depression |
| Choi, 2014, 2016 [136, 137] | Problem-solving therapy | RCT; video call therapy (1 in-person + 5 video) vs. in-person therapy (6 sessions) vs. telephone support | Depressed homebound older adults (age ≥50 yr) with low-income | 158 (56 video, 63 in-person, 39 support)  Excluded active suicidal ideation, possible dementia, bipolar disorder or other mental illness, active alcohol or other substance abuse | Both counselling methods were efficacious; video effects were sustained longer than in-person treatment (effect at 36 weeks 0.68 video vs. 0.20 in-person for Hamilton Rating Scale for Depression and 0.47 and 0.25 for WHO Disability Assessment Schedule)  There was wide variation in suicide ideation at baseline, making relative changes due to treatment difficult to interpret; authors indicate telephone more effective than in-person, however, support calls had similar effect |
| Egede, 2015, 2016, 2017, 2018a,2018b [138-142]  Charleston (SC, USA)  Apr 2007-Jul 2011  NCT00324701 | Behavioural activation therapy | RCT; non-inferiority; 8 sessions by telemedicine (videoconference using analogue videophone) or in-person  Subgroup with diabetes [142] | Veterans age ≥58 yr with major depressive disorder  Excluded psychotic, demented, suicidal ideation or intent, substance dependence | 120 telemedicine, 121 in-person  100 telemedicine and 104 in-person included in the per-protocol analysis  Subgroup of 90 patients with diabetes (43 telemedicine, 47 in-person) | Treatment response was not significantly different using Geriatric Depression Scale, Beck Depression Inventory, or the Structured Clinical Interview for DSM-IV; criteria for non-inferiority were met  No difference in quality-of-life scores at end of study period, small differences over some time points  Diabetes: telemedicine was better at maintaining A1c levels |
| Luxton, 2016 [143]; Smolenski, 2017 [144]; Pruitt, 2019 [145]  Aug 2012- Jul 2014  Military treatment facilities in Washington state and Portland (Oregon) | Behavioural activation therapy | RCT, noninferiority trial; 8 sessions by videoconference or in-office | US military service members and veterans with major or minor depressive disorder | 121  87 (45 in-home, 42 in-person) completed post-treatment outcome assessment including satisfaction | Similar reductions in hopelessness and depressive symptoms but did not prove non-inferiority; slight benefit of in-person care on some clinical outcomes  Subgroup with less symptom severity at baseline: no difference in treatment modalities  Subgroup with higher symptom severity at baseline: video group had less improvement than in person; this group included more patients that were older, higher loneliness, and higher anxiety at baseline  No significant differences in patient satisfaction; baseline characteristics differentiated those with preferences. Veterans, those with lower-rank, more severe symptoms, less education were more likely to prefer in-home care |
| **Anxiety, stress, panic** | | | | | |
| Bouchard, 2020 [146]  ISRCTN76456442 | CBT | 2-arm intent-to-treat non-randomized non-inferiority prospective study; videoconferencing (from psychologist office) vs. in-person for 12 weekly sessions  Rural area (Maniwaki) and urban area (Montreal) allocated to video; local urban site (Gatineau) allocated to face-to face | People with panic disorder and agoraphobia, age 18-65 yr, no concurrent psychotherapy or other mental disorder | 71 (40 video, 31 face-to-face)  55 received allocated intervention (31 and 24)  45 (27 and 18) completed 12-month follow-up | Panic disorder, agoraphobia, fear of sensations and depressed mood showed significant improvements; no differences in video or face-to-face delivery  Video noninferior for primary outcome of global panic disorder and agoraphobia severity, as well as 2 secondary outcomes (agoraphobia and depressed mood); did not reach statistical significance for fear of body sensations |
| Milosevic, 2021 [147]  Anxiety Treatment and Research Clinic, St Joseph’s Healthcare, Hamilton, Ontario  May 2018-Dec 2019 face-to-face; May-Sep 2020 video | CBT | Non-randomized, group face-to-face pre-COVID-19 or videoconference (since COVID-19 pandemic), 12 sessions by psychologist or social worker  Separate protocols for social anxiety disorder, panic disorder and/or agoraphobia, generalized anxiety disorder, obsessive-compulsive disorder | Adult outpatients (age ≥18 yr) of tertiary care anxiety disorders clinic with diagnosis of anxiety or related disorders | 413 | Face-to-face had slight benefit over videoconference overall (p<0.001) for symptom severity; for individual groups was significant difference only for generalized anxiety disorder (p<0.008)  Dropout 15.3% video vs. 24.5% face-to-face (p=0.08); slightly higher attendance in videoconference for generalized anxiety disorder (p=0.045) |
| Watts, 2020 [148]  Quebec  Mar 2014-Dec 2016 | CBT based on the Intolerance of Uncertainty Model of generalized anxiety disorder | RCT; videoconference vs. conventional in-person psychotherapy, 15 sessions  Videoconferencing was located at local clinic and remotely connected to psychotherapist | Adults (age 18-75) with generalized anxiety disorder recruited from specialized anxiety disorder clinics in Quebec | 148 randomized  Analyzed 115 (50 video, 65 conventional) | Telepsychotherapy did not interfere with working alliance; alliance was higher in the video group |
| Alcantara, 2016 [129]  -is subset of depression study, Alegria, 2014 [128]  May 2011 – Sep 2012 | CBT - engagement and counseling for Latinos (ECLA) | RCT; telephone vs. face-to-face vs. usual care | Low-income Latinos recruited from primary care; Boston (Massachusetts) and San Juan (Puerto Pico) | 257 (87 telephone, 84 face-to-face, 86 usual care) | Reduction in worry at 4 months from randomization was lower in counselling groups than usual care (p<0.05), and this was greater for telephone than face-to-face (p<0.05) |
| **Posttraumatic Stress Disorder (PTSD)** | | | | | |
| Acierna, 2016 [149]  A large Southeastern VA medical centre | Behavioral activation and therapeutic exposure | RCT, noninferiority; home (video) vs. in clinic, 8 sessions | Veterans with full criteria or predefined subthreshold PTSD and major depression | 232 | Symptom improvement occurred and was comparable in both groups post-treatment and at 3- and 12-months follow-up; home-based therapy was noninferior |
| Acierno, 2017 [150]; Gros, 2018 [151]  Ralph H. Johnson Veterans Affairs Medical Center (VA) and the Medical University of South Carolina  Nov 2010 – Apr 2015 | Prolonged exposure therapy | RCT, noninferiority; home vs. in-person, | Veterans with PTSD | 150 randomized; 133 initiated therapy (65 home, 68 in-person) | Home therapy was noninferior to reduce PTSD scores at posttreatment and at 3- and 6-month follow-up  Home therapy was noninferior for depression only at 6 months  Disability status and treatment condition (telehealth or in-person) were predictors of discontinuation of treatment prior to completion of 8 sessions |
| Acierno, 2021 [152]  NCT02417025  A VAMC in the Southeastern United States  Oct 2014 – Sep 2019 | Prolonged exposure therapy | RCT, home (videoconference) vs. in-person | Women veterans with military sexual trauma survivor-related PTSD | 136 (69 home, 67 in-person) | No differences in dose of therapy (number of sessions) received or PTSD symptom reduction; dose was related to reduction in symptom severity |
| White, 2021 [153]  Subset of data from Acierno 2017, 2021 [150, 152] | Prolonged exposure therapy | RCT, home (videoconference) vs. in-person  Data from 2 concurrent studies by same investigators | Satisfaction data from subset of patients who reported this outcome in 2 studies | 140 | Symptom improvement was associated with greater satisfaction. Two tools did not give consistent results as to whether home or in-person groups were more satisfied  There is a need for additional satisfaction studies and measurement tools |
| Morland, 2020 [154]  San Diego VA Healthcare System  2012-2018 | Prolonged exposure therapy | RCT; home-based telehealth vs. office-based telehealth vs. in-home-in-person; 6-15 sessions depending on treatment response | Within 35 miles of San Diego VA Medical Center, PTSD with specific memory of the traumatic event | 58 home telehealth, 59 office telehealth, 58 in person | Clinical effectiveness did not differ by treatment modality  Higher discontinuation (<8 sessions and not meeting definition of early responder) in telehealth arms (home vs. in-person OR=2.67, p=0.031; office telehealth vs. in-person OR=5.08, p<0.001) |
| Morland 2014, [155]  NCT00879255  4 Veterans Affairs clinical sites and 3 Vet Centres across Hawaii  March 2009 – June 2013 | Cognitive processing therapy | RCT, non-inferiority; videoconference vs. in-person for 12 sessions | Rural ethnically diverse sample of male combat veterans with PTSD | 61 video, 64 in-person | Significant reduction in PTSD severity at posttreatment, 3 months, and 6 months; video was noninferior to in-person treatment  High therapeutic alliance, treatment compliance, satisfaction with no difference between groups |
| Morland, 2015 [156]  NCT02362477  Department of Veterans Affairs, Honolulu, Hawaii  March 2009 – June 2013 | Cognitive processing therapy | RCT, non-inferiority; videoconference vs. in-person for 12 sessions | Ethnically diverse sample of women with PTSD, included 21 veterans and 105 civilians | 126 (63 video, 63 in-person) | Substantial improvement in PTSD symptoms posttreatment and at 3- and 6-months follow-up; veterans had less improvement than civilians  Video was noninferior to in-person treatment |
| Glassman, 2019 [157] | Cognitive processing therapy | Videoconferencing vs. in-person  Secondary analysis of two RCTS: The Men’s study [155] and The Women’s study [156] | See above | 125 male veterans; 126 female veterans and civilians | No effect of treatment modality on changes in QoL scores over time; scores improved at end of treatment and 3 months, but declined at 6-month follow-up  Video was noninferior to in-person therapy |
| Liu, 2020 [158] | Cognitive processing therapy | RCT, noninferiority; videoconference vs. in-person for 12 sessions  VA San Diego Healthcare System | Adults (age ≥18 yr) with primary diagnosis of PTSD | 207 (103 video, 104 in-person); 65 video and 73 in-person were completers of the intervention | Video group improvement in symptom severity was inferior to in-person at the end of treatment but was noninferior at 6-month follow-up  Secondary outcomes of patient-reported outcomes of stress and depressive symptoms indicated video was noninferior in patients who completed treatment |
| Maieritsch, 2016 [159]  Two Midwestern VHA hospitals in the US  2008-2013 | Cognitive processing therapy | RCT; videoconference vs. in-person | Veterans of Iraq/ Afghanistan conflict receiving mental health services and diagnosed with PTSD due to a military-related traumatic event | 90 (45 video, 45 in-person); 51 analyzed (25 video, 26 in-person) | High drop-out rate (43.3%) and therefore underpowered  Trend suggesting video and in-person may be equivalent (p=0.094)  Analysis of both groups combined found significant reduction of PTSD symptoms and depression |
| Morland, 2010, 2011 [160, 161]; Greene, 2010 [162]  Aug 2005 – Oct 2008  NCT00122109 | Group CBT for anger management | RCT, non-inferiority; group therapy by videoconference vs. in-person  3 VA clinical sites and 3 Vet Centres across Hawaii | Male veterans with PTSD and anger difficulties | 160 randomized; 125 participated (61 video, 64 in-person)  Secondary analysis of 112 patients who completed the intervention [162] | Both groups had significant and clinically meaningful reductions in anger symptoms at posttreatment and at 3 months, and 6 months follow-up; effect was similar (noninferior) in video and in-person groups  No between group differences in attrition, adherence, satisfaction  Video group reported lower therapeutic alliance (mean 4.2 vs. 4.5)  Therapist adherence to protocol was excellent and was similar in both groups |
| Valentine, 2020 [163]  2010-2016 | Prolonged exposure therapy or cognitive processing therapy | Participant choice prolonged exposure therapy or cognitive processing therapy and of video or in person treatment | Veterans with PTSD following military sexual trauma; excluded patients deemed at too high of risk for remote care | 171 (31 video); 56.4% cognitive processing therapy and 43.6% prolonged exposure therapy | Full completion did not differ significantly by treatment delivery; video users less likely to complete a minimally adequate care number of sessions  Results may be related to patient characteristics related to self-selection into treatment groups |
| **Pain** | | | | | |
| Kelleher, 2019 [164]; Check, 2021 [165]  Duke University Cancer Center  2014-2017 | CBT – pain coping skills training | RCT, noninferiority; videoconference vs. in-person, 4 sessions | Diagnosis of breast, lung, prostate, or colorectal cancer within the last 2 yr, age ≥18 yr, two clinical pain ratings of ≥3 (on a scale of 0 to 10) | 178 (89 video, 89 in-person)  Secondary analysis of 135 who completed at least one follow-up assessment [165] | Video had higher feasibility (attrition, adherence, time to complete)  Similar patient burden, engagement, and acceptability  Video noninferior for all intervention outcomes (pain severity, pain interference, physical symptoms, psychological distress, physical well-being, self-efficacy) except for physical symptoms at 3-months posttreatment  In both groups combined, 34% had reduction in pain severity and 46% had reduction in pain interference; reduction in pain interference was greater in colorectal than breast cancer |
| Herbert, 2017 [166]  VA San Diego Healthcare System (VASDHS) facilities in the San Diego area  NCT01055639  Apr 2010 – Apr 2013 | CBT-acceptance and commitment therapy | RCT, noninferiority; videoconference vs. in-person, 8-week therapy | Veterans with chronic pain, age 25-89 yr | 128 (63 video, 65 in-person)  111 started treatment (52 video, 59 in-person) | Significant improvement in all outcomes in both groups (pain interference, pain severity, mental and physical health-related QoL, pain acceptance, activity level, depression, pain-related anxiety) except for sleep quality  Improvement in activity level at 6 months was greater in the in-person group  Overall, video was noninferior  Treatment satisfaction similar but more in video group withdrew |
| Levy, 2017 [167]  4 pediatric GI clinics in USA  2012-2015 | Social learning and CBT | RCT; telephone vs. in-person therapy (3 sessions of 1 hour) vs. control (phone education and support)  Interventions were directed at the parents (not children) | Parents of children aged 7-12 yr with at least weekly functional abdominal pain or irritable bowel syndrome, no physical or laboratory findings that would explain the pain | 316 dyads (parent/child) | Treatment did not affect pain severity, parent and child-reported gastrointestinal symptoms, or child-reported QoL or coping  Therapy improved parental solicitousness, pain beliefs, and catastrophizing, and additional outcomes of parent-reported functional disability, pain behaviours, child health care visits for abdominal pain  Only telephone therapy improved QoL and missed school days |
| Chavooshi, 2017 [168]  An academic health centre in Tehran, Iran | Intensive short-term dynamic psychotherapy | RCT; videoconferencing vs. in-person | Medically unexplained pain of duration ≥6 months, age 18-45 yr | 81 (39 video, 42 in-person)  Most patients were on psychiatric medication and had severe/ extreme depression or anxiety | In-person group had significantly lower pain intensity and greater improvement in depression, anxiety, emotion regulation function, mindfulness, and QoL than video both at end of intervention and 12-month follow-up  Dropout rate 33% video and 23% in-person |
| **Insomnia** | | | | | |
| Arnedt, 2021 [169]  University of Michigan, Ann Arbor, MI  Nov 2017 – Jun 2019  NCT03293745 | CBT | RCT, noninferiority; telemedicine (video) vs. face-to-face CBT (6 sessions) to improve insomnia/sleep and daytime functioning | Chronic insomnia, age ≥18 yr, met ICSD-3 criteria for chronic insomnia disorder | 65 (33 telemedicine; 32 face-to-face) | Telemedicine was noninferior at posttreatment and follow up  Daytime functioning (except physical composite scale) significantly improved, no difference between groups  Therapeutic alliance similar in both groups |
| **Eating Disorder** | | | | | |
| Crow, 2009 [170]; Mitchell, 2008 [171]; Marrone, 2009 [172]; Ertelt, 2011 [173]  9 regional sites in eastern North Dakota and northwestern Minnesota  Oct 1999 – Sep 2003 | CBT for bulimia nervosa | RCT, telemedicine vs. face-to-face, 20 sessions over 16 weeks  Telemedicine system linking regional healthcare system facility to remote psychologist | Women age ≥18 yr with DSM-IV bulimia nervosa or eating disorder not otherwise specified, body weight ≥85% of ideal weight | 128 (62 telemedicine, 66 face-to-face) | Telemedicine costs less ($7,300 vs. $9,325) mostly due to therapist travel cost  Telemedicine and face-to-face were similarly effective; abstinence rates at end of treatment were slightly higher for face-to-face but not statistically significant difference  Face-to-face group had more reduction in eating disordered cognitions and depression, but this was of marginal significance  Patient ratings of session adherence to therapeutic tasks and goals did not differ between groups |
| Zerwas, 2017 [174]; Watson, 2017a, 2018 [175, 176]  University of North Carolina at Chapel Hill; University of Pittsburgh Medical Center  NCT00877786 | CBT for bulimia nervosa | RCT, noninferiority; therapeutic online chat group (synchronous) vs. face-to-face group, 16 sessions over 20 weeks | DSM-IV diagnosis of bulimia nervosa, age ≥18 yr, BMI ≥18.5 kg/m^2^ | 179 (89 chat, 90 face-to-face) | Internet inferior at post-treatment but noninferior at 1-yr follow-up for abstinence from binge eating and purging  Mean cost per abstinent patient at posttreatment $11,870 chat vs. $7,757 face-to-face  Mean cost per abstinent patient at 1-yr follow-up was $14,561 chat vs. $16,777 face-to-face  Differences in cost were not statistically significant  Out-of-pocket costs were lower for chat group ($50 vs. $178) |
| **Malnutrition** | | | | | |
| Lindegaard Pedersen, 2017 [177] | Nutritional counselling by clinical dietitian | RCT; telephone vs. home visit vs. no follow-up (control) after hospital discharge | Patients malnourished or at risk of malnutrition (MNA<24), aged 75 yr and older, living at home and alone | 208 (68 telephone, 73 home visit, 67 control) | Risk of readmission to hospital was significantly lower with home visit than control; risk was lower with telephone than control but not significant  Risk of readmission after home visit was significantly lower than in the telephone group at 30 and 90 days |
| **Obsessive Compulsive Disorder** | | | | | |
| Lovell, 2006 [178]  2 psychology outpatient departments, greater Manchester, UK  2001-2002  ISRCTN500103984 | CBT - exposure therapy and response prevention | RCT, noninferiority; telephone vs. face-to-face, 10 weekly sessions | Obsessive Compulsive Disorder was main presenting problem, age 16-65 yr | 72 (36 telephone, 36 face-to-face) | Patient satisfaction high in both groups  Clinical outcome was equivalent |
| Turner, 2014 [179]; Nair, 2019 [180]; Tie, 2019 [181]  UK National Health Service  2008-2011  ISRCTN27070832 | CBT | RCT, noninferiority, telephone vs. face-to-face, 14 sessions with the child plus 10 minutes at end of each session including the parent | Children aged 11-18 yr with obsessive compulsive disorder by DSM‑IV criteria and their parents | 72 child and their parents (36 telephone, 36 face-to-face) | Telephone noninferior to face-to-face at posttreatment, 3 months, 6 months; at 12 months there was no difference, but confidence intervals exceeded noninferiority threshold  Both groups reported high level of satisfaction  Total health and social care cost less for video (mean £2475 vs. £2965) but difference not significant  No significant group difference obsessive-compulsive scale or quality-adjusted life years; video should be considered clinically noninferior alternative when access to in-person care is limited or patient prefers video |
| **Coping with cancer** | | | | | |
| Watson, 2017b [182]; Rodrigues, 2021 [183]  Royal Marsden Hospital | CBT | RCT, prospective equivalence; telephone vs. face-to-face; median 4 sessions (range 1-9) | Age ≥18 yr with cancer diagnosis (except non-melanoma skin cancer) | 118 (60 telephone, 58 in-person) | Significant improvement (p<0.01) for both groups in anxiety, depression, cancer concerns  Reduction in cancer coping stress and worry compared to baseline for both groups combined (p=0.003)  In person group had significant change in positive focus and helpless/hopeless  Equivalence was not observed but telephone was noninferior and can be offered according to patient choice |
| Lleras de Frutos, 2020 [184]  Comprehensive cancer network in Barcelona  Jan 2016 – Jan 2019 NCT03010371 | Positive psychotherapy | Pragmatic RCT; videoconference group positive psychotherapy vs. face-to-face group positive psychotherapy by clinical psychologists | Adult women with range of cancer diagnoses and emotional distress at end of primary oncological treatment | 269 (124 online vs. 145 face-to-face)  Most patients were randomized, but those with strong preference could choose group (16 online, 28 face-to-face) | Emotional distress decreased significantly over time; post-traumatic growth increased significantly  No significant differences between video and face-to-face groups |
| Guzman, 2020 [185] | Psychosocial counselling | Retrospective chart review; Outreach care by either video or telephone vs. face-to-face outpatient care in the Supportive Care Center.  Outreach care is offered to those who require more immediate follow-up, and is video or telephone according to patient preference | Patients with cancer in outpatient palliative care clinic with psychological distress | 2072 patients, of which 1620 outpatient only and 452 outpatient plus outreach  404 outreach patients had also had a face-to-face counsellor encounter first; 48 outreach patients had seen a physician but not counsellor | Outpatient only group: 70% had 1 session and 29% had 2-5 sessions; Outpatient + outreach group 5% had 1 session, 69% had 2-5 sessions  Of Outreach encounters, 94% telephone, 4% facetime, 2% video |
| **Mental health** | | | | | |
| Morgan, 2008 [186]  USA | Mental health services | Video vs. face-to-face with psychologist; assigned to modality available at the institution | Adult male inmates who received mental health services in an adult correctional institution | Psychology: 50 face-to-face, 36 telemental (videoconferencing) | No significant difference in working alliance, patient’s evaluation (depth, smoothness, positivity), satisfaction (quality, met needs) |
| Ospina-Pinillos, 2018 [187]  Jul 2015 to Aug 2016 | Assessment of clinical stage in youth mental health | Both online and standard face-to-face assessment, order was randomized and counterbalanced 1:3.  Online include web-based survey plus video visit with clinician | Attendees of youth-specific mental health services in Sydney Australia, ages 16-25 yr | 72 | Fair agreement (kappa=0.39, p<0.001) with concordance in 68% of cases |
| **Counselling (various)** | | | | | |
| Fukkink, 2009a,b [188, 189] | Helpline counselling | Online chat service of the Dutch Kindertelefoon vs. telephone service | Children aged 8-18 yr consulting Kinderteefoon, Amsterdam, Netherlands who chose to participate in study and answered pre- and post-intervention questions | 902 with pretest and posttest: 339 chat (80% girls), 563 telephone (71% girls)  213 with follow-up test (119 chat, 94 telephone) | Children had higher sense of well-being and reduced problem severity after consultation  Chat was slightly more favourable  There were differences in groups between type of support, length of interaction, topic, age; chat group were older and seeking more emotional support compared to information |
| King, 2006 [190] | Helpline counselling | Kids Help Line, Australia; online (real-time text exchange) vs. telephone. Naturalistic, user determined method of counselling; single session of 50-80 minutes online or 45-60 minutes by telephone | Young people in Australia who called helpline and who agree prior to counselling to be part of study and who completed pre- and post-counselling measure (GHQ12) | 86 online (mean age 15.4 yr, 95% female, pre-GHQ 39.0)  101 telephone (mean age 13.1 yr, 66% female, pre-GHQ 32.9) | Online group was older, higher distress, and more females  Counselling had positive effect in both groups, larger in the telephone group  Telephone group had higher total alliance, lower resistance, higher collaboration, no significant difference in mutual liking and acceptance  Pre-session GHQ significantly predicted post-session GHQ |
| Murphy, 2009 [191]  Apr 2006-May 2008 | Counselling | Patients screened by telephone and offered online counselling if considered appropriate, otherwise face-to-face counselling | Online clients who completed assessment; convenience sample of face-to-face clients | GAF score: 26 online and 101 face-to-face  CSS score: 45 online and 43 face-to-face | GAF (Global Assessment of Function): differences in initial assessment, change (improvement) was similar in both groups  CSS (Client Satisfaction Survey): excluding 3 outliers, the results were similar between groups |
| Laver, 2020 [192] | Dyadic dementia care program by occupational therapist | RCT; telehealth (2 visits in home + 6 videoconference) vs. home (8 sessions in home) | People with dementia or probable dementia and a caregiver | 63 | Improvement, but not significant, in both groups  No significant differences between groups  Intervention is feasible |
| **Relationship counselling** | | | | | |
| Wade, 2019 [193]  CHIPS study, NCT02368366 | Family problem solving treatment | RCT, therapist online vs. face-to-face. Randomized if <25 miles from hospital, otherwise assigned to on-line therapy  Patient and family completed up to 10 core session and 4 focusing additional sessions on areas of concern of each family | Parents of adolescents with traumatic brain injury and behavioural impairment | 56 online, 34 face-to-face | Baseline depression and psychiatric symptoms were less than cut points at baseline and decreased in both groups. Whether changes are clinically significant was not reported |
| Corona, 2021 [194]  Was shift to telemedicine due to COVID-19 pandemic | Behavioural intervention and support for families of children assessed for autism spectrum disorder | Telemedicine (video), in-person, or hybrid for up to 6 sessions of 60-90 minutes each; provided by board-certified behaviour analysis, speech-language pathologists, or early childhood educators with expertise in autism spectrum disorder and behaviour analysis | Caregivers of young children (age 16-33 months) with or evaluated for autism spectrum disorder | 115 families: 78% autism spectrum disorder, 13% developmental delay, 9% other | Satisfied with service, positive child outcomes  Slightly less improvement in telemedicine group for some domains as rated by parents (caregiving routines, social interactions), or as rated by consultants (play, nonverbal communication, social interactions)  Completion rate for 6 sessions was 80% telemedicine, in-person 78%, hybrid 90% |
| **HIV** | | | | | |
| Kalichman, 2021a [195] | Self-regulation counselling to improve HIV treatment outcomes | RCT using Wennberg Randomized Preferential Design; telephone vs. in-office | HIV and at risk for discontinuing care and treatment failure, living in rural are of southeastern USA | 251 | 69% of patients preferred telephone counselling and completed more sessions  Few differences in clinical appointment attendance, antiretroviral adherence, and HIV viral load. No difference in overall health outcomes  Telephone group had more improvement in depression, in-office group had greater reduction in alcohol use |
| Kalichman, 2021b [196]  Central Georgia (USA), June 2017-Jul 2018 | Behavioural self-regulation theory | RCT, telephone vs. in-clinic, second randomization to either community nurse or paraprofessional patient navigator.  Up to 6 sessions of counselling followed for monthly telephone assessments of treatment adherence and daily text messages regarding alcohol use for 12 months | Patients age 18+ receiving HIV care and identified as in‑need due to history of poor retention of care, non-adherence to antiretroviral therapy, or treatment failure | 77 telephone, 80 in-person, 83 education control | Both counselling groups benefited in short term, with better adherence to treatment than non-treatment controls  Alcohol used decreased more with in-person counselling than telephone or control, no other differences  No difference between nurse and patient navigator counselling |
| Phanuphak, 2018 [197]  Dec 2015-Jun 2017  NCT03203265  Thailand | Counselling | Patient choice of on-line counselling and HIV self-testing vs. on-line pre-test counselling + in-person HIV testing and post-test counselling vs. in-person HIV counselling and testing | Thai men who have sex with men and transgender women | 211 online, 158 mixed, 202 in-person | On-line group had more first-time testers and highest HIV prevalence  First-time testers: 47.3% online, 18.1% mixed, 42.4% in-person  HIV prevalence: 15.9% online, 3.4% mixed, 13.0% in-person  Providers used smartphones 79.2% and laptops 37.5%  Concluded online HIV services are feasible and can engage first-time testers and those with high prevalence |
| **Diabetes** | | | | | |
| Delahanty, 2018, 2020 [198, 199]  REAL HEALTH-diabetes trial  NCT02320253 | Modified look AHEAD lifestyle intervention for weight loss in type 2 diabetes | RCT; registered dietician delivered telephone vs. in-person group lifestyle intervention vs. standard of care (nutrition therapy)  37 sessions over 2 yr, plus 5 optional sessions | Type 2 diabetes, age 18+, overweight or obese, HbA1c of 6.5% to 11.5% | 72 telephone, 70 in-person, 69 control | Weight loss at 6 and 12 months: 4.6% and 4.8% telephone, 5.6% and 4.6% in-person, 1.1% and 2.0% control; both intervention arms significantly different than control but not each other  HbA1c improved in all participants  Cost per kg weight loss: $1223 telephone vs. $789 in-person |
| Duke, 2016 [200]; Harris, 2015 [201]; Riley, 2015 [202]  US Pacific Northwest | Behavioural family systems therapy – diabetes (BFST-D) | RCT; telehealth (videoconference) vs. in-clinic, up to 10 sessions of 1 to 1.5 hours each over 12 weeks provided by psychologist | Adolescents (age 12-19 yr) with type 1 diabetes and suboptimal glycemic control (HbA1c ≥9.0%) | 90 adolescents and their parents (46 telehealth, 44 clinic) | Both groups had improvement in family conflict, adjustment to illness, glycemic control, adherence, depressive symptoms; no significant differences between groups |
| Nevanpera, 2015 [203]  Finland | Constructivism-based dietary group counselling | Videoconference or face-to-face counselling depending on place of residence in northern Finland; 5 video (90-minute ) or six in-person group sessions by registered nutritionist | High risk of type 2 diabetes based on Finnish Diabetes Risk Score of 12 points or more or elevated fasting blood glucose 6.1-6.9 mmol/L or impaired glucose tolerance (7.8-11 mmol/L) in past 12 months | 72 | Cognitive restraint eating increased and uncontrolled eating decreased between baseline and 6 months in both groups; benefit was maintained at 21 months (although no longer significant for video group)  Emotional eating decreased but this was only significant in the video group |
| **Drugs and alcohol** | | | | | |
| Harder, 2020 [204]  Primary health centre in Kenya  Oct 2014-Mar 2015 | Motivational interviewing to encourage change in alcohol use | RCT; one session of immediate (mobile telephone), in-person, or delayed (wait list) | Patients age ≥18 yr at health centre who screened positive for alcohol use problems | 300 randomized but 230 participated: 89 mobile, 65 in-person, 76 control | Alcohol Use Disorder Identification Test (AUDIT) and the shorter AUDIT-C used at baseline, 1 month, 6 months  Both groups improved compared to control; no difference between mobile and in-person groups at 1 month and inconclusive at 6 months |
| **Contraception** | | | | | |
| Lohr, 2018 [205]  2011-2014 | Contraceptive counselling prior to abortion consultation | User selects method: telephone consultation prior to but separate from abortion consultation vs. face-to-face integrated into abortion consultation | Pregnant women seeking abortion at British Pregnancy Advisory Service clinics and received contraception there | 18,753; 31.2% telephone counselling, 68.8% integrated counselling | Tier 1: implant, intrauterine device, sterilization  Tier 2: injectable, pill, patch, ring, diaphragm  Tier 3: condoms, withdrawal, sponge, fertility awareness, spermicide  Telephone group included more non-White racial/ethnic groups (33.8% vs. 22.1%) and had more difficulty obtaining contraception in the past (40.4% vs. 3.0%)  Telephone group chose more tier 1 (54.1% vs. 42.6%) and tier 2 (34.7% vs. 30.7%) methods |
| **Physical activity** | | | | | |
| James, 2017 [206]  Australia  2011-2014 | Physical activity counselling | Pragmatic RCT: 1 face-to face and 4 telephone calls vs. 5 face-to-face sessions with exercise specialist vs. usual care control (mailed brochure) | Insufficiently active primary care practice patients, 70% female, aged ≥18 yr | 203 randomized: 64 telephone, 68 in-person, 71 control | Intervention groups had mean of 1,002 more steps/day at 12 months (p=0.01) compared to usual care  Face-to-face group had 619 more steps/day than telephone group (p=0.27) |
| **Smoking cessation** | | | | | |
| Andrews, 2016 [207]; Baker, 2015, 2018 [208, 209]  Australia | Psychosocial treatment: healthy lifestyles intervention | RCT; 90 min face to face session plus nicotine replacement therapy; randomized to 14 telephone calls (about 10 min) + 2 face-to-face sessions vs. 16 face-to-face Healthy Lifestyles Intervention sessions | Smokers with psychotic disorder | 235 randomized; 211 attended at least one session | Phone intervention included content areas of Health Lifestyles intervention used in person, but less intensively and without CBT or contingent reinforcement  Early alliance did not predict treatment retention overall, although some elements predict some outcomes  Mean sessions attended: 12.4 telephone vs. 9.2 in person  By sessions 2-3, 67% telephone and 49% in-person reported using nicotine replacement therapy; by 12 months this was 88% vs. 85%. No difference in smoking reduction status between groups but was more in those who attended more sessions  Was reduction in cardiovascular disease risk overall; this was not different between groups  No significant differences from baseline in quality of life, mental health indices, health behaviours  At 36 months follow-up, was significant reductions in cardiovascular disease risk and smoking in both groups but no between group differences; changes in other outcomes were modest except for significant improvement in both groups for depression and global functioning |
| Berndt, 2016 [210]  ACTRN12609001039279  The Netherlands | Smoking cessation counselling interventions | RCT; telephone counselling for 3 months + nicotine replacement therapy vs. face-to-face counselling + nicotine replacement therapy vs. usual care (standard in-hospital treatment including smoking assessment, brief quit advice, sometimes a brochure).  Randomization was by institution, then a cross-over | Cardiac patients in hospital wards who smoked prior to admission | 223 telephone, 157 face-to-face, 245 usual care | Telephone counselling was by professional telephone counselors from the Dutch Expert Center for Tobacco Control, included 7 calls of 10-15 min each over 3 months  In-person counselling was by nurses trained in providing cessation counselling, including 6 sessions of 30-45 min each over 3 months plus telephone call 5 weeks later  No significant group differences in quality-adjusted life-years; higher continued abstinence in counselling groups than usual care; no difference between the counselling groups  Telephone more cost effective; both counselling more cost-effective than usual care |
| Byaruhanga, 2020a,b, 2021 [211-213]  ACTRN12617000514303  Australia | CBT and motivational interviewing | RCT; real-time video (6 sessions) vs. telephone counselling (6 sessions) vs. written materials | Daily tobacco users age ≥18 yr in rural or remote areas of New South Wales, Australia | 201 video, 229 telephone, 225 written material | No difference in retention rates  93.5% video and 96.2% telephone indicated mode was acceptable or very acceptable; both were rated better than control in motivation to quit and coping with cravings but no difference between video and telephone groups  Interim analysis of abstinence at 4 months: video and telephone similar (18.9% and 12.7%); video was better than control (8.9%)  13-month data not yet available |
| Carlson, 2012 [214]  Sept 2005-Apr 2008  Calgary and rural sites (16 Alberta, 1 NWT) | Large-group behavioural smoking cessation intervention | Group counselling by video to rural sites vs. in-person to Calgary residents; 8 sessions of 90 min over 15 weeks led by clinical psychologists | Smokers referred by family physicians and centre staff or self-referral by pamphlets, websites, other media | 184 video, 370 in-person | Rural sites were connected to in-person meetings by video so were part of the same meetings and could ask questions; short separations of time for remote group to have smaller local discussions  Continuous abstinence 25.5% rural, 27.5% Calgary by intent-to-treat analysis; 37.2% vs. 39.2% using available data |
| Nomura, 2019 [215]  Mar-Jun 2018  Japan | Smoking cessation program | Non-inferiority RCT; 12 weeks of video vs. face-to-face visits; both groups received mobile exhaled carbon monoxide checker | Nicotine-dependent adults | 58 internet-based video, 57 face-to-face | Continuous abstinence rates similar weeks 9-12 (81.0% vs. 78.9%); conclude video not inferior to in-person program |
| Richter, 2015 [216]  NCT00843505  Kansas | Smoking cessation counselling | RCT; Real-time video by computer/webcam in clinic exam rooms vs. telephone | Smokers from primary care and safety net clinics in Kansas, smoked at least 5 cigarettes/day | 566 (280 video, 286 telephone) | Abstinence at 12 months similar (9.8% vs. 12%, p=0.406)  Video group used more cessation medications (55.9% vs. 46.1%, p-0.03); had higher patient satisfaction, and was more likely to be recommended to others  Phone was less costly from societal perspective as video was conducted in the clinic |
| **Weight loss, obesity** | | | | | |
| Appel, 2011 [217]; Dalcin, 2015 [218]; Daumit, 2020 [219]  NCT00783315  2008-2009 | Behavioural weight-loss interventions; motivational interviewing  Practice-based opportunities for weight reduction (POWER) Hopkins trial | RCT; remote (telephone, website, and email) vs. in-person (individual and group sessions, telephone if visits created a hardship) vs. self-directed control group; delivered by trained lifestyle coaches | Obese patients with at least one cardiovascular risk factor (hypertension, hyper-cholesterolemia, or diabetes), recruited from primary care practices in Baltimore area | 415 (139 remote, 138 in-person, 138 control) | Change in weight at 24 months (compared to baseline): -4.6 kg vs. -5.1 kg vs. -0.8 kg  Loss of ≥5% of initial weight: 38.2% vs. 41.4% vs. 18.8%  Remote intervention cost at 6 months was $99/kg lost vs. $117/kg lost; at 24 months it was $275/kg lost vs. $342/kg lost |
| Befort, 2021 [220]; Kurz, 2021 [221]  24 primary care practices in rural Midwestern US  Enrolment Feb 2016-Oct 2017 | Lifestyle intervention  The Rural Engagement in Primary Care for Optimizing Weight Reduction (REPOWER) trial | RCT, randomized at clinic level; telephone conference calls vs. group visits in clinic for 2 yr | Patients with obesity in rural clinics, aged 20-75 yr and BMI of 30-45 kg/m^2^ | 466 telephone group vs. 468 clinic group | Mean weight loss at 24 months: telephone group intervention -3.9 kg, in-clinic group intervention -4.4 kg  Patients in telephone group reported lower satisfaction than in-clinic group |
| Fujii, 2017 [222]  2011-2012  Japan | Health counselling to prevent metabolic syndrome | RCT, noninferiority; remote (headphones and computer screen) vs. face-to-face | Obese people aged 30-75 yr | 150 (66 remote and 73 face-to-face had intervention) | Video was not inferior (within 20%) to in-person weight loss (-2.84% vs. -2.22%) |
| Harrigan, 2016 [223]  The Lifestyle, Exercise, and Nutrition (LEAN) Study | Counselling focused on reducing caloric intake, increasing physical activity, and behavioural therapy | RCT; telephone vs. in-person counselling vs. usual care (pamphlets, referred to 2-session weight management program).  Counselling was 11 sessions (30 min each) over 6 months | Women treated for breast cancer, BMI ≥25 kg/m^2^ | 100 (34 telephone, 33 in-person, 33 usual care) | Average 6-month weight loss was 5.4%, 6.4%, and 2.0% for telephone, in-person, and usual care groups  C-reactive protein decreased 30% in counselling groups vs. 1% decrease in usual care |
| Harvey-Berino, 1998 [224]  Vermont, USA | Behavioural weight-control treatment | RCT, interactive television vs. in-person group treatment; treatment was 12 consecutive weeks by behavioural therapist. Only participants at university site were randomized.  Live group in studio and broadcast to interactive television sites | Over 18 yr old, ≥20% over ideal body weight | 133 television, 33 in-person | No difference in weight loss, calories, exercise changes |
| Lukenbill, 2021 [225]  Jan 2017 – Sept 2019  Charleston, SC and rural areas | Weight management clinic | Retrospective cohort study, noninferiority; telehealth for rural patients with child in primary care physician office and video connection to Heart Health dietitian vs. in-person counselling by pediatrician and dietitians at Heart Health Clinics.  In-person patients had option of ancillary exercise sessions and weekly group sessions | Pediatric patients (age 2-22) visiting weight management clinic | 58 telehealth, 1019 in-person | In person 47.9% Black, 23.7% White, 28.4% Other; in telehealth 29.3% Black, 19.0% White, 51.7% Other  Telehealth was non-inferior to in-person visits |
| Lutes, 2013 [226]; Damschroder, 2014 [227]  Vimalananda, 2016 [228]  2010-2012 | Behavioural treatment and motivational interviewing with small changes (SC) approach  ASPIRE-VA Trial | RCT by stratified random blocks; SC telephone (individual) vs. SC in-person (group) vs. usual care (MOVE!)  SC groups met with health coaches 28 times (weekly then gradually longer intervals) over 12 months | Overweight / obese veterans, BMI ≥30 kg/m^2^ or between 25 and 30 mg/m^2^ with at least one obesity-related chronic health condition | 481 | Weight loss at 12 months: -1.4 kg telephone, -2.8 kg in-person, -1.4 kg MOVE! control; when analyzed by sex, for women there was weight gain on telephone but equal loss for both in-person group and MOVE!; for men all 3 groups lost weight, with most for in-person, then telephone, and least for MOVE! (only difference between in-person and MOVE! was significant)  The in-person group had better improvement in anthropometric measures compared to MOVE! and all except waist circumference compared to telephone  Women reported greater satisfaction with ASPIRE SC programs then MOVE! |
| Lutes, 2017 [229] | Behavioural treatment with small changes (SC) approach  Extension of ASPIRE-VA trial | Participants in ASPIRE-VA were invited to extend participation in the same group for another year; included 6 sessions of SC by telephone or in-person group or 12 sessions of MOVE! | See above | 332 | At 24 months from baseline was modest weight loss in all groups: -2.13 kg telephone, -1.4 kg in-person, -1.78 kg MOVE! (no significant differences)  Patients with diabetes benefited initially with in-person (-2.6 kg) but had 2.8 kg regain |

Abbreviations: BMI, body mass index; CBT, cognitive behavioural therapy; DSM-IV, Diagnostic and Statistical Manual of Mental Disorders, 4th edition; GHQ, General Health Questionnaire; HbA1c, hemoglobin A1c; HIV, human immunodeficiency virus; ICSD-3, International classification of sleep disorders-third edition; MNA, Mini Nutritional Assessment; OR, odds ratio; PTSD, posttraumatic stress disorder; QoL, quality of life; RCT, randomized controlled trial

Table S15. Systematic reviews on virtual versus in-person counselling

| **Source** | **Topic** | **Patient population** | **Included studies** | **Review conclusions** |
| --- | --- | --- | --- | --- |
| Fernandez, 2021 [230] | Video-delivered psychotherapy | 1681 participants in within-group studies and 3564 patients in between-group studies receiving live video psychotherapy | 47 between-group studies and 56 within-group studies | Video-delivered psychotherapy was significantly better in outcome than wait list controls (p=0.77) but negligible in difference from in-person psychotherapy  No clinical difference in efficacy between videoconferencing and in-person, with efficacy most pronounced in CBT for affective disorders  Live psychotherapy by video emerges not only as a popular and convenient choice but also one that is now upheld by meta-analytic evidence |
| Thomas, 2021 [231] | Videoconferencing psychotherapy | Adult populations experiencing adult mental health disorders or clinically significant symptoms of mental disorder participating in one-on-one psychological interventions delivered via videoconferencing | 21 RCTs and 48 other studies | Videoconferencing has an established evidence base in the delivery of cognitive behavioural therapies for posttraumatic stress disorder and depression, with prolonged exposure, cognitive processing therapy, and behavioural activation non-inferior to in-person delivery  Large trials found similar efficacy for health anxiety and bulimia nervosa  Sometimes more difficult to judge non-verbal behaviour by videoconferencing; patients find similar therapeutic alliance, videoconferencing may be less confronting  Videoconferencing may be useful in embedding therapy delivery within the client's own environment |
| Byaruhanga, 2020 [232] | Video counselling | Patients participating in real-time video communication interventions on smoking, nutrition, alcohol consumption, physical activity, and obesity | 13 RCTs | Video counselling is potentially more effective than a control group or other modes of support in addressing physical inactivity and obesity and is not less effective in modifying smoking and alcohol consumption |
| Irvine, 2020 [233] | Telephone psychological therapy | Patients receiving telephone psychological therapy for mental health problems | 8 experimental studies and 7 observational studies | Telephone does not have a detrimental effect on interactional aspects of psychological therapy |
| Ho 2018b [234]  CADTH rapid response report | E-therapy interventions for depression | Adult patients with diagnosed depression with or without co-morbid mental health conditions | 4 RCTs and 3 systematic reviews | Therapist-guided e-therapy was found to be equivalent to standard face-to-face CBT for patients with major depressive disorder |
| Turgoose, 2018 [235] | Tele-therapy for posttraumatic stress disorder | Ex-military personnel with PTSD receiving tele-therapy interventions via video or telephone | 28 experimental studies and 14 non-experimental studies | Tele-therapy is viable alternative and could increase access to therapy  Tele-therapy should continue to be evaluated to determine the most effective delivery methods |
| Jobes, 2020 [236] | Telepsychotherapy for suicidal risk during COVID-19 | At risk of suicide  This is not a systematic review but appears the most relevant publication; it notes other reviews and points to resources |  | Collaborative Assessment and Management of Suicidality (CAMS) may be useful [see <http://www.cams-care.com/> for resources]  Some Dialectical Behaviour Therapy resources are also mentioned |
| Rosen, 2020 [237] | Telepsychotherapy during a pandemic | Synthesis of findings from prior reviews (not new systematic review) |  | Reviews literature on the mental health impact of pandemics, crisis counselling approaches developed from prior disasters, and clinical research on telepsychotherapy treatment posttraumatic stress disorder  Telepsychotherapy may be used as a preventive intervention to help people cope with distress during a period of disruption, life-threat, and loss, and as treatment for chronic conditions in response to traumatic stress |

Abbreviations: CADTH, Canadian Agency for Drugs and Technologies in Health; CBT, cognitive behavioural therapy; RCT, randomized controlled trial

**REFERENCES**

19. Ho K, Lauscher HN, Stewart K, Abu-Laban RB, Scheuermeyer F, Grafstein E, et al. Integration of virtual physician visits into a provincial 8-1-1 health information telephone service during the COVID-19 pandemic: A descriptive study of HealthLink BC Emergency iDoctor-in-assistance (HEiDi). CMAJ Open. 2021;9(2):E635-E41. doi:10.9778/cmajo.20200265.

20. Newbould J, Exley J, Ball S, Corbett J, Pitchforth E, Roland M. GPs' and practice staff's views of a telephone first approach to demand management: A qualitative study in primary care. Br J Gen Pract. 2019a;69(682):e321-e8. doi:10.3399/bjgp19X702401.

21. Newbould J, Ball S, Abel G, Barclay M, Brown T, Corbett J, et al. A ‘telephone first’ approach to demand management in English general practice: A multimethod evaluation. Health Serv Deliv Res. 2019b;5:5. doi:10.3310/hsdr07170.

22. Ball SL, Newbould J, Corbett J, Exley J, Pitchforth E, Roland M. Qualitative study of patient views on a 'telephone-first' approach in general practice in England: Speaking to the GP by telephone before making face-to-face appointments. BMJ Open. 2018;8(12):e026197. doi:10.1136/bmjopen-2018-026197.

23. Newbould J, Abel G, Ball S, Corbett J, Elliott M, Exley J, et al. Evaluation of telephone first approach to demand management in English general practice: Observational study. BMJ. 2017;358:j4197. doi:10.1136/bmj.j4197.

24. Miller D, Loftus AM, O'Boyle PJ, McCloskey M, O'Kelly J, Mace D, et al. Impact of a telephone-first consultation system in general practice. Postgrad Med J. 2019;95(1129):590-5. doi:10.1136/postgradmedj-2019-136557.

25. Jiwa M, Mathers N, Campbell M. The effect of GP telephone triage on numbers seeking same-day appointments. Br J Gen Pract. 2002;52(478):390-1.

26. Edwards HB, Marques E, Hollingworth W, Horwood J, Farr M, Bernard E, et al. Use of a primary care online consultation system, by whom, when and why: Evaluation of a pilot observational study in 36 general practices in South West England. BMJ Open. 2017;7(11):e016901. doi:10.1136/bmjopen-2017-016901.

27. Farr M, Banks J, Edwards HB, Northstone K, Bernard E, Salisbury C, et al. Implementing online consultations in primary care: A mixed-method evaluation extending normalisation process theory through service co-production. BMJ Open. 2018;8(3):e019966. doi:10.1136/bmjopen-2017-019966.

28. Holt TA, Fletcher E, Warren F, Richards S, Salisbury C, Calitri R, et al. Telephone triage systems in UK general practice: Analysis of consultation duration during the index day in a pragmatic randomised controlled trial. Br J Gen Pract. 2016;66(644):e214-8. doi:10.3399/bjgp16X684001.

29. Varley A, Warren FC, Richards SH, Calitri R, Chaplin K, Fletcher E, et al. The effect of nurses' preparedness and nurse practitioner status on triage call management in primary care: A secondary analysis of cross-sectional data from the ESTEEM trial. Int J Nurs Stud. 2016;58:12-20. doi:10.1016/j.ijnurstu.2016.02.001.

30. Warren FC, Calitri R, Fletcher E, Varley A, Holt TA, Lattimer V, et al. Exploring demographic and lifestyle associations with patient experience following telephone triage by a primary care doctor or nurse: Secondary analyses from a cluster randomised controlled trial. BMJ Qual Saf. 2015;24(9):572-82. doi:10.1136/bmjqs-2015-003937.

31. Calitri R, Warren FC, Wheeler B, Chaplin K, Fletcher E, Murdoch J, et al. Distance from practice moderates the relationship between patient management involving nurse telephone triage consulting and patient satisfaction with care. Health Place. 2015;34:92-6. doi:10.1016/j.healthplace.2015.04.002.

32. Campbell JL, Fletcher E, Britten N, Green C, Holt T, Lattimer V, et al. The clinical effectiveness and cost-effectiveness of telephone triage for managing same-day consultation requests in general practice: A cluster randomised controlled trial comparing general practitioner-led and nurse-led management systems with usual care (the ESTEEM trial). Health Technol Assess. 2015;19(13):1-212, vii-viii. doi:10.3310/hta19130.

33. Campbell JL, Fletcher E, Britten N, Green C, Holt TA, Lattimer V, et al. Telephone triage for management of same-day consultation requests in general practice (the ESTEEM trial): A cluster-randomised controlled trial and cost-consequence analysis. Lancet. 2014;384(9957):1859-68. doi:10.1016/S0140-6736(14)61058-8.

34. Murdoch J, Varley A, Fletcher E, Britten N, Price L, Calitri R, et al. Implementing telephone triage in general practice: a process evaluation of a cluster randomised controlled trial. BMC Fam Pract. 2015;16(47). doi:10.1186/s12875-015-0263-4.

35. Lawless M, Wright E, Davidson J. A collaborative approach to improving patient access in general practice: Impact of three different pilot schemes in 12 general practices in Greenwich. London J Prim Care (Abingdon). 2016;8(4):56-65. doi:10.1080/17571472.2016.1173946.

36. Villarreal M, Leach J, Ngianga-Bakwin K, Dale J. Can a partnership between general practitioners and ambulance services reduce conveyance to emergency care? Emerg Med J. 2017;34(7):459-65. doi:10.1136/emermed-2015-204924.

37. Siddiqui F, Sidhu B, Tahir MA. Using 'Active Signposting' to streamline general practitioner workload in two London-based practices. BMJ Open Qual. 2017;6(2):e000146. doi:10.1136/bmjoq-2017-000146.

38. Elliott M, Jones S, Johnson C, Wallace C. What are the benefits of nurse-led triage in primary care? Prim Health Care. 2020;30(3). doi:10.7748/phc.2020.e1607.

39. Huibers L, Moth G, Carlsen AH, Christensen MB, Vedsted P. Telephone triage by GPs in out-of-hours primary care in Denmark: A prospective observational study of efficiency and relevance. Br J Gen Pract. 2016;66(650):e667-73. doi:10.3399/bjgp16X686545.

40. Jansen T, Hek K, Schellevis FG, Kunst AE, Verheij RA. Income-related differences in out-of-hours primary care telephone triage using national registration data. Emerg Med J. 2021;38(6):460-6. doi:10.1136/emermed-2020-209649.

41. Eccles A, Hopper M, Turk A, Atherton H. Patient use of an online triage platform: A mixed-methods retrospective exploration in UK primary care. Br J Gen Pract. 2019;69(682):e336-e44. doi:10.3399/bjgp19X702197.

42. Christensen MB, Noroxe KB, Moth G, Vedsted P, Huibers L. Drug prescriptions in Danish out-of-hours primary care: A 1-year population-based study. Scand J Prim Health Care. 2016;34(4):453-8. doi:10.1080/02813432.2016.1248622.

43. Lake R, Georgiou A, Li J, Li L, Byrne M, Robinson M, et al. The quality, safety and governance of telephone triage and advice services - an overview of evidence from systematic reviews. BMC Health Serv Res. 2017;17(1):614. doi:10.1186/s12913-017-2564-x.

44. Rushton S, Boggan JC, Lewinski AA, Gordon AM, Shoup JP, Van Voorhees E, et al. Effectiveness of remote triage: A systematic review. Washington (DC): Department of Veterans Affairs; 2019 Jul 29 [modified 2020 Jan 6; cited 2021 Nov 26]. Available from: https://www.hsrd.research.va.gov/publications/esp/reports.cfm.

45. Dixon RF, Stahl JE. A randomized trial of virtual visits in a general medicine practice. J Telemed Telecare. 2009;15(3):115-7. doi:10.1258/jtt.2009.003003.

46. Stahl JE, Dixon RF. Acceptability and willingness to pay for primary care videoconferencing: A randomized controlled trial. J Telemed Telecare. 2010;16(3):147-51. doi:10.1258/jtt.2009.090502.

47. McKinstry B, Walker J, Campbell C, Heaney D, Wyke S. Telephone consultations to manage requests for same-day appointments: A randomised controlled trial in two practices. Br J Gen Pract. 2002;52(477):306-10.

48. McKinstry B, Hammersley V, Burton C, Pinnock H, Elton R, Dowell J, et al. The quality, safety and content of telephone and face-to-face consultations: A comparative study. Qual Saf Health Care. 2010;19:298-303. doi:10.1136/qshc.2008.027763.

49. Gujral K, Scott JY, Ambady L, Dismuke-Greer CE, Jacobs J, Chow A, et al. A primary care telehealth pilot program to improve access: Associations with patients' health care utilization and costs. Telemed J E Health. 2021;https://dx.doi.org/10.1089/tmj.2021.0284. doi:10.1089/tmj.2021.0284.

50. Llorian ER, Mason G. Healthcare utilization and telemedicine: An evaluation using linked administrative data from Manitoba. J Telemed Telecare. 2021;https://dx.doi.org/10.1177/1357633X20981227. doi:10.1177/1357633X20981227.

51. Reed M, Huang J, Graetz I, Muelly E, Millman A, Lee C. Treatment and follow-up care associated with patient-scheduled primary care telemedicine and in-person visits in a large integrated health system. JAMA Netw Open. 2021;4(11):e2132793. doi:10.1001/jamanetworkopen.2021.32793.

52. Graetz I, Huang J, Muelly E, Gopalan A, Lee C, Reed ME. Patient choice of telemedicine increases timeliness of primary care visits. AMIA Annu Symp Proc. 2020;2020:502-3.

53. Reed ME, Huang J, Graetz I, Lee C, Muelly E, Kennedy C, et al. Patient characteristics associated with choosing a telemedicine visit vs office visit with the same primary care clinicians. JAMA Netw Open. 2020;3(6):e205873. doi:10.1001/jamanetworkopen.2020.5873.

54. Ryskina KL, Shultz K, Zhou Y, Lautenbach G, Brown RT. Older adults' access to primary care: Gender, racial, and ethnic disparities in telemedicine. J Am Geriatr Soc. 2021;69(10):2732-40. doi:10.1111/jgs.17354.

55. Cecil E, Bottle A, Majeed A, Aylin P. Factors associated with potentially missed acute deterioration in primary care: Cohort study of UK general practices. Br J Gen Pract. 2021;71(708):e547-e54. doi:10.3399/BJGP.2020.0986.

56. Gonzalez F, Cimadevila B, Garcia-Comesana J, Cerqueiro S, Andion E, Prado J, et al. Telephone consultation in primary care. J Health Organ Manag. 2018;32(2):321-37. doi:10.1108/JHOM-08-2017-0201.

57. Ross AA, Yap TL, Nest JV, Martin K, Edie AH. Increasing primary care access close to home for residents of remote communities in northern Alberta. Healthc Q. 2016;19(3):61-6. doi:10.12927/hcq.2016.24863.

58. Bernstein P, Ko KJ, Israni J, Cronin AO, Kurliand MM, Shi JM, et al. Urgent and non-emergent telehealth care for seniors: Findings from a multi-site impact study. J Telemed Telecare. 2021;https://dx.doi.org/10.1177/1357633X211004321. doi:10.1177/1357633X211004321.

59. Lovell T, Albritton J, Dalto J, Ledward C, Daines W. Virtual vs traditional care settings for low-acuity urgent conditions: An economic analysis of cost and utilization using claims data. J Telemed Telecare. 2021;27(1):59-65. doi:10.1177/1357633X19861232.

60. Ohta M, Ohira Y, Uehara T, Keira K, Noda K, Hirukawa M, et al. How accurate are first visit diagnoses using synchronous video visits with physicians? Telemed J E Health. 2017;23(2):119-29. doi:10.1089/tmj.2015.0245.

61. Gordon AS, Adamson WC, DeVries AR. Virtual visits for acute, nonurgent care: A claims analysis of episode-level utilization. J Med Internet Res. 2017;19(2):e35. doi:10.2196/jmir.6783.

62. Grech CK, Laux MA, Burrows HL, Macy ML, Pomeranz ES. Pediatric emergency department resource utilization among children with primary care clinic contact in the preceding 2 days: A cross-sectional study. J Pediatr. 2017;188:245-51.e2. doi:10.1016/j.jpeds.2017.06.007.

63. Abrashkin KA, Washko JD, Li T, Berkowitz J, Poku A, Zhang J, et al. Video or telephone? A natural experiment on the added value of video communication in community paramedic responses. Ann Emerg Med. 2021;77(1):103-9. doi:10.1016/j.annemergmed.2020.04.026.

64. Al Mazrouei N, Ibrahim RM, Al Meslamani AZ, Abdel-Qader DH, Mohamed Ibrahim O. Virtual pharmacist interventions on abuse of over-the-counter medications during COVID-19 versus traditional pharmacist interventions. J Am Pharm Assoc (2003). 2021;61(3):331-9. doi:10.1016/j.japh.2021.02.003.

65. Carrillo de Albornoz S, Sia KL, Harris A. The effectiveness of teleconsultations in primary care: Systematic review. Fam Pract. 2021;39(1):168-82. doi:10.1093/fampra/cmab077.

66. Hui D, Dolcine B, Loshak H. CADTH health technology review: Approaches to evaluations of virtual care in primary care. Environmental scan. Can J Health Technol. 2022;2(1). doi:10.51731/cjht.2022.238.

67. Gray C, Mason J, Loshak H. CADTH horizon scan: An overview of direct-to-patient virtual visits in Canada. Can J Health Technol. 2021;1(6). doi:10.51731/cjht.2021.80.

68. Totten AM, Hansen RN, Wagner J, Stillman L, Ivlev I, Davis-O’Reilly C, et al. Telehealth for acute and chronic care consultations. Comparative effectiveness review no. 216. (Prepared by Pacific Northwest Evidence-based Practice Center under Contract No. 290-2015-00009-I.) AHRQ Publication No. 19-EHC012-EF. Rockville, MD: Agency for Healthcare Research and Quality; April 2019 [cited 2022 Jan 31]. Available from: 10.23970/AHRQEPCCER216.

69. Totten AM, McDonagh MS, Wagner JH. The evidence base for telehealth: Reassurance in the face of rapid expansion during the COVID-19 pandemic. White paper commentary. (Pacific Northwest Evidence-based Practice Center, Oregon Health & Science University under Contract No. 290-2015-00009-I). AHRQ Publication No. 20-EHC015. Rockville, MD: Agency for Healthcare Research and Quality. May 2020 [cited 2022 Jan 31]. Available from: 10.23970/AHRQEPCCOVIDTELEHEALTH.

70. Totten AM, Womack DM, Eden KB, McDonagh MS, Griffin JC, Grusing S, et al. Telehealth: mapping the evidence for patient outcomes from systematic reviews. Technical Brief 26 (ARCHIVED). Agency for Healthcare Research and Quality (US), Rockville (MD); 2016 [cited 2022 Jan 31]. Available from: https://effectivehealthcare.ahrq.gov/products/telehealth/technical-brief.

71. Schifeling CH, Shanbhag P, Johnson A, Atwater RC, Koljack C, Parnes BL, et al. Disparities in video and telephone visits among older adults during the COVID-19 pandemic: Cross-sectional analysis. JMIR Aging. 2020;3(2):e23176. doi:10.2196/23176.

72. Eberly LA, Kallan MJ, Julien HM, Haynes N, Khatana SAM, Nathan AS, et al. Patient characteristics associated with telemedicine access for primary and specialty ambulatory care during the COVID-19 pandemic. JAMA Netw Open. 2020;3(12):e2031640. doi:10.1001/jamanetworkopen.2020.31640.

73. Schenker RB, Laguna MC, Odisho AY, Okumura MJ, Burnett H. Are we reaching everyone? A cross-sectional study of telehealth inequity in the COVID-19 pandemic in an urban academic pediatric primary care clinic. Clin Pediatr (Phila). 2021;61(1):26-33. doi:10.1177/00099228211045809.

74. Lopez Segui F, Hernandez Guillamet G, Pifarre Arolas H, Marin-Gomez FX, Ruiz Comellas A, Ramirez Morros AM, et al. Characterization and identification of variations in types of primary care visits before and during the COVID-19 pandemic in Catalonia: Big data analysis study. J Med Internet Res. 2021;23(9):e29622. doi:10.2196/29622.

75. van der Velden AW, Bax EA, Bongard E, Munck Aabenhus R, Anastasaki M, Anthierens S, et al. Primary care for patients with respiratory tract infection before and early on in the COVID-19 pandemic: An observational study in 16 European countries. BMJ Open. 2021;11(7):e049257. doi:10.1136/bmjopen-2021-049257.

76. Brown CL, Montez K, Amati JB, Simeonsson K, Townsend JD, Orr CJ, et al. Impact of COVID-19 on pediatric primary care visits at four academic institutions in the Carolinas. Int J Environ Res Public Health. 2021;18(11):27. doi:10.3390/ijerph18115734.

77. Murphy M, Scott LJ, Salisbury C, Turner A, Scott A, Denholm R, et al. Implementation of remote consulting in UK primary care following the COVID-19 pandemic: A mixed-methods longitudinal study. Br J Gen Pract. 2021;71(704):e166-e77. doi:10.3399/BJGP.2020.0948.

78. Sigurdsson EL, Blondal AB, Jonsson JS, Tomasdottir MO, Hrafnkelsson H, Linnet K, et al. How primary healthcare in Iceland swiftly changed its strategy in response to the COVID-19 pandemic. BMJ Open. 2020;10(12):e043151. doi:10.1136/bmjopen-2020-043151.

79. Mohamed Ibrahim O, Ibrahim RM, Abdel-Qader DH, Al Meslamani AZ, Al Mazrouei N. Evaluation of telepharmacy services in light of COVID-19. Telemed J E Health. 2021;27(6):649-56. doi:10.1089/tmj.2020.0283.

80. Penza KS, Murray MA, Myers JF, Furst JW, Pecina JL. Management of acute sinusitis via e-visit. Telemed J E Health. 2021;27(5):532-6. doi:10.1089/tmj.2020.0047.

81. Murray MA, Penza KS, Myers JF, Furst JW, Pecina JL. Comparison of evisit management of urinary symptoms and urinary tract infections with standard care. Telemed J E Health. 2020;26(5):639-44. doi:10.1089/tmj.2019.0044.

82. Shi Z, Mehrotra A, Gidengil CA, Poon SJ, Uscher-Pines L, Ray KN. Quality of care for acute respiratory infections during direct-to-consumer telemedicine visits for adults. Health Aff (Millwood). 2018;37(12):2014-23. doi:10.1377/hlthaff.2018.05091.

83. Ray KN, Shi Z, Gidengil CA, Poon SJ, Uscher-Pines L, Mehrotra A. Antibiotic prescribing during pediatric direct-to-consumer telemedicine visits. Pediatrics. 2019;143(5):05. doi:10.1542/peds.2018-2491.

84. Ray KN, Martin JM, Wolfson D, Schweiberger K, Schoemer P, Cepullio C, et al. Antibiotic prescribing for acute respiratory tract infections during telemedicine visits within a pediatric primary care network. Acad Pediatr. 2021;21(7):1239-43. doi:10.1016/j.acap.2021.03.008.

85. Ewen E, Willey VJ, Kolm P, McGhan WF, Drees M. Antibiotic prescribing by telephone in primary care. Pharmacoepidemiol Drug Saf. 2015;24(2):113-20. doi:10.1002/pds.3686.

86. Frost HM, Sebastian T, Durfee J, Jenkins TC. Ophthalmic antibiotic use for acute infectious conjunctivitis in children. J AAPOS. 2021;25(6):350 e1- e7. doi:10.1016/j.jaapos.2021.06.006.

87. Penza KS, Murray MA, Myers JF, Maxson J, Furst JW, Pecina JL. Treating pediatric conjunctivitis without an exam: An evaluation of outcomes and antibiotic usage. J Telemed Telecare. 2020;26(1-2):73-8. doi:10.1177/1357633X18793031.

88. Bakhit M, Baillie E, Krzyzaniak N, van Driel M, Clark J, Glasziou P, et al. Antibiotic prescribing for acute infections in synchronous telehealth consultations: A systematic review and meta-analysis. BJGP Open. 2021;5(6):BJGPO.2021.0106. doi:10.3399/BJGPO.2021.0106.

89. Han SM, Greenfield G, Majeed A, Hayhoe B. Impact of remote consultations on antibiotic prescribing in primary health care: Systematic review. J Med Internet Res. 2020;22(11):e23482. doi:10.2196/23482.

90. Phillips JC, Lord RW, Davis SW, Burton AA, Kirk JK. Comparing telehealth to traditional office visits for patient management in the COVID-19 pandemic: A cross-sectional study in a respiratory assessment clinic. J Telemed Telecare. 2021;https://dx.doi.org/10.1177/1357633X21990197. doi:10.1177/1357633X21990197.

91. Tarn DM, Hintz C, Mendez-Hernandez E, Sawlani SP, Bholat MA. Using virtual visits to care for primary care patients with COVID-19 symptoms. J Am Board Fam Med. 2021;34(Suppl):S147-S51. doi:10.3122/jabfm.2021.S1.200241.

92. Riese A, Kelly JM, Chu TC, Heinly A, Kamath S, Golova N, et al. Visits for possible COVID-19 in a pediatric primary care practice early in the pandemic. R I Med. 2021;104(6):43-8.

93. Irving G, Lawson D, Tinsley A, Parr H, Whittaker C, Jones H, et al. Evaluation of a 'drop box' doorstep assessment service to aid remote assessments for COVID-19 in general practice. BMJ Open Qual. 2021;10(1):03. doi:10.1136/bmjoq-2020-001081.

94. Gruffydd-Jones K, Hollinghurst S, Ward S, Taylor G. Targeted routine asthma care in general practice using telephone triage. Br J Gen Pract. 2005;55(521):918-23.

95. Pinnock H, Adlem L, Gaskin S, Harris J, Snellgrove C, Sheikh A. Accessibility, clinical effectiveness, and practice costs of providing a telephone option for routine asthma reviews: Phase IV controlled implementation study. Br J Gen Pract. 2007;57(542):714-22.

96. Pinnock H, Bawden R, Proctor S, Wolfe S, Scullion J, Price D, et al. Accessibility, acceptability, and effectiveness in primary care of routine telephone review of asthma: Pragmatic, randomised controlled trial. BMJ. 2003;326(7387):477-9. doi:10.1136/bmj.326.7387.477.

97. Al Harthi T, Anwar H, Al Lawati A, Al Shuriqi F, Al Rashdi F, Al Mahrouqi A, et al. The impact of Covid-19 on diabetes care in Muscat Governorate: A Retrospective Cohort Study In Primary Care. J Prim Care Community Health. 2021;12:21501327211051930. doi:10.1177/21501327211051930.

98. Lu AD, Gunzburger E, Glorioso TJ, Smith WB, 2nd, Kenney RR, Whooley MA, et al. Impact of longitudinal virtual primary care on diabetes quality of care. J Gen Intern Med. 2021;36(9):2585-92. doi:10.1007/s11606-020-06547-x.

99. Turner BJ, Liang Y, Ramachandran A, Poursani R. Telephone or visit-based community health worker care management for uncontrolled diabetes mellitus: A longitudinal study. J Community Health. 2020;45(6):1123-31. doi:10.1007/s10900-020-00849-1.

100. Wickstrom H, Oien RF, Midlov P, Anderberg P, Fagerstrom C. Pain and analgesics in patients with hard-to-heal ulcers: Using telemedicine or standard consultations. J Wound Care. 2021;30(Suppl 6):S23-S32. doi:10.12968/jowc.2021.30.Sup6.S23.

101. Wickstrom HL, Oien RF, Fagerstrom C, Anderberg P, Jakobsson U, Midlov PJ. Comparing video consultation with inperson assessment for Swedish patients with hard-to-heal ulcers: Registry-based studies of healing time and of waiting time. BMJ Open. 2018;8(2):e017623. doi:10.1136/bmjopen-2017-017623.

102. Eibl JK, Gauthier G, Pellegrini D, Daiter J, Varenbut M, Hogenbirk JC, et al. The effectiveness of telemedicine-delivered opioid agonist therapy in a supervised clinical setting. Drug Alcohol Depend. 2017;176:133-8. doi:10.1016/j.drugalcdep.2017.01.048.

103. Rivera J, Shcherbakova N, Vala C, Capoccia K. Community pharmacists' interventions and documentation during medication therapy management encounters delivered face-to-face versus via telephone: The devil is in the details. Res Social Adm Pharm. 2020;16(10):1447-51. doi:10.1016/j.sapharm.2019.12.020.

104. Ho C, Argáez C. Telehealth-delivered opioid agonist therapy for the treatment of adults with opioid use disorder: Review of clinical effectiveness, cost-effectiveness, and guidelines. CADTH rapid response reports: Summary with critical appraisal. Ottawa: Canadian Agency for Drugs and Technologies in Health; 2018 Oct 5 [cited 2022 Mar 18]. Available from: https://www.cadth.ca/telehealth-delivered-opioid-agonist-therapy-treatment-adults-opioid-use-disorder-review-clinical.

105. Barth J, Nickel F, Kolominsky-Rabas PL. Diagnosis of cognitive decline and dementia in rural areas - A scoping review. Int J Geriatr Psychiatry. 2018;33(3):459-74. doi:10.1002/gps.4841.

106. Grossman D, Grindlay K. Safety of medical abortion provided through telemedicine compared with in person. Obstet Gynecol. 2017;130(4):778-82. doi:10.1097/aog.0000000000002212.

107. Grossman DA, Grindlay K, Buchacker T, Potter JE, Schmertmann CP. Changes in service delivery patterns after introduction of telemedicine provision of medical abortion in Iowa. Am J Public Health. 2013;103(1):73-8. doi:10.2105/ajph.2012.301097.

108. Grossman D, Grindlay K, Buchacker T, Lane K, Blanchard K. Effectiveness and acceptability of medical abortion provided through telemedicine. Obstet Gynecol. 2011;118(2 Pt 1):296-303. doi:10.1097/AOG.0b013e318224d110.

109. Grossman D, Baum SE, Andjelic D, Tatum C, Torres G, Fuentes L, et al. A harm-reduction model of abortion counseling about misoprostol use in Peru with telephone and in-person follow-up: A cohort study. PLoS One. 2018;13(1):(no pagination). doi:10.1371/journal.pone.0189195.

110. Cameron ST, Glasier A, Dewart H, Johnstone A, Burnside A. Telephone follow-up and self-performed urine pregnancy testing after early medical abortion: A service evaluation. Contraception. 2012;86(1):67-73. doi:10.1016/j.contraception.2011.11.010.

111. Maddison R, Rawstorn JC, Stewart RAH, Benatar J, Whittaker R, Rolleston A, et al. Effects and costs of real-time cardiac telerehabilitation: Randomised controlled non-inferiority trial. Heart. 2019;105(2):122-9. doi:10.1136/heartjnl-2018-313189.

112. Scalvini S, Zanelli E, Comini L, Dalla Tomba M, Troise G, Febo O, et al. Home-based versus in-hospital cardiac rehabilitation after cardiac surgery: A nonrandomized controlled study. Phys Ther. 2013;93(8):1073-83. doi:10.2522/ptj.20120212.

113. Moffet H, Tousignant M, Nadeau S, Mérette C, Boissy P, Corriveau H, et al. In-home telerehabilitation compared with face-to-face rehabilitation after total knee arthroplasty: A noninferiority randomized controlled trial. J Bone Joint Surg Am. 2015;97(14):1129-41. doi:10.2106/jbjs.N.01066.

114. Piqueras M, Marco E, Coll M, Escalada F, Ballester A, Cinca C, et al. Effectiveness of an interactive virtual telerehabilitation system in patients after total knee arthoplasty: A randomized controlled trial. J Rehabil Med. 2013;45(4):392-6. doi:10.2340/16501977-1119.

115. Kuether J, Moore A, Kahan J, Martucci J, Messina T, Perreault R, et al. Telerehabilitation for total hip and knee arthroplasty patients: A pilot series with high patient satisfaction. HSS J. 2019;15(3):221-5. doi:10.1007/s11420-019-09715-w.

116. Nicola K, Waugh J, Charles E, Russell T. The feasibility and concurrent validity of performing the Movement Assessment Battery for Children - 2nd Edition via telerehabilitation technology. Res Dev Disabil. 2018;77:40-8. doi:10.1016/j.ridd.2018.04.001.

117. Ewald B, Stacey F, Johnson N, Plotnikoff RC, Holliday E, Brown W, et al. Physical activity coaching by Australian Exercise Physiologists is cost effective for patients referred from general practice. Aust N Z J Public Health. 2018;42(1):12-5. doi:10.1111/1753-6405.12733.

118. Cottrell MA, O'Leary SP, Swete-Kelly P, Elwell B, Hess S, Litchfield MA, et al. Agreement between telehealth and in-person assessment of patients with chronic musculoskeletal conditions presenting to an advanced-practice physiotherapy screening clinic. Musculoskelet Sci Pract. 2018;38:99-105. doi:10.1016/j.msksp.2018.09.014.

119. Inglis SC, Clark RA, Dierckx R, Prieto-Merino D, Cleland JG. Structured telephone support or non-invasive telemonitoring for patients with heart failure. Cochrane Database Syst Rev. 2015 (10):Cd007228. doi:10.1002/14651858.CD007228.pub3.

120. Anderson L, Sharp GA, Norton RJ, Dalal H, Dean SG, Jolly K, et al. Home-based versus centre-based cardiac rehabilitation. Cochrane Database Syst Rev. 2017 (6):CD007130-CD. doi:10.1002/14651858.CD007130.pub4.

121. Jin K, Khonsari S, Gallagher R, Gallagher P, Clark AM, Freedman B, et al. Telehealth interventions for the secondary prevention of coronary heart disease: A systematic review and meta-analysis. Eur J Cardiovasc Nurs. 2019;18(4):260-71. doi:10.1177/1474515119826510.

122. Cottrell MA, Galea OA, O'Leary SP, Hill AJ, Russell TG. Real-time telerehabilitation for the treatment of musculoskeletal conditions is effective and comparable to standard practice: A systematic review and meta-analysis. Clin Rehabil. 2017;31(5):625-38. doi:10.1177/0269215516645148.

123. Jiang S, Xiang J, Gao X, Guo K, Liu B. The comparison of telerehabilitation and face-to-face rehabilitation after total knee arthroplasty: A systematic review and meta-analysis. J Telemed Telecare. 2018;24(4):257-62. doi:10.1177/1357633x16686748.

124. Shukla H, Nair SR, Thakker D. Role of telerehabilitation in patients following total knee arthroplasty: Evidence from a systematic literature review and meta-analysis. J Telemed Telecare. 2016;23(2):339-46. doi:10.1177/1357633X16628996.

125. Rawstorn JC, Gant N, Direito A, Beckmann C, Maddison R. Telehealth exercise-based cardiac rehabilitation: A systematic review and meta-analysis. Heart. 2016;102(15):1183-92. doi:10.1136/heartjnl-2015-308966.

126. Mani S, Sharma S, Omar B, Paungmali A, Joseph L. Validity and reliability of Internet-based physiotherapy assessment for musculoskeletal disorders: A systematic review. J Telemed Telecare. 2017;23(3):379-91. doi:10.1177/1357633x16642369.

127. Canadian Agency for Drugs and Technologies in Health. Telehealth for speech and language pathology: A review of clinical effectiveness, cost-effectiveness, and guidelines. CADTH Rapid Response Reports. Ottawa: Canadian Agency for Drugs and Technologies in Health; 2015 Apr 7 (cited 2022 Mar 18). Available from: https://www.cadth.ca/telehealth-speech-and-language-pathology-review-clinical-effectiveness-cost-effectiveness-and.

128. Alegria M, Ludman E, Kafali EN, Lapatin S, Vila D, Shrout PE, et al. Effectiveness of the Engagement and Counseling for Latinos (ECLA) intervention in low-income Latinos. Med Care. 2014;52(11):989-97. doi:10.1097/MLR.0000000000000232.

129. Alcantara C, Li X, Wang Y, Canino G, Alegria M. Treatment moderators and effectiveness of Engagement and Counseling for Latinos intervention on worry reduction in a low-income primary care sample. J Consult Clin Psychol. 2016;84(11):1016-22. doi:10.1037/ccp0000146.

130. Kirkness CJ, Cain KC, Becker KJ, Tirschwell DL, Buzaitis AM, Weisman PL, et al. Randomized trial of telephone versus in-person delivery of a brief psychosocial intervention in post-stroke depression. BMC Res Notes. 2017;10(1):500. doi:10.1186/s13104-017-2819-y.

131. Byun E, Becker KJ, Kohen R, Kirkness CJ, Mitchell PH. Brief psychosocial intervention to address poststroke depression may also benefit fatigue and sleep-wake disturbance. Rehabil Nurs. 2021;46(4):222-31. doi:10.1097/RNJ.0000000000000304.

132. Mohr DC, Ho J, Duffecy J, Reifler D, Sokol L, Burns MN, et al. Effect of telephone-administered vs face-to-face cognitive behavioral therapy on adherence to therapy and depression outcomes among primary care patients: A randomized trial. JAMA. 2012;307(21):2278-85. doi:10.1001/jama.2012.5588.

133. Kalapatapu RK, Ho J, Cai X, Vinogradov S, Batki SL, Mohr DC. Cognitive-behavioral therapy in depressed primary care patients with co-occurring problematic alcohol use: effect of telephone-administered vs. face-to-face treatment-a secondary analysis. J Psychoactive Drugs. 2014;46(2):85-92. doi:10.1080/02791072.2013.876521.

134. Stiles-Shields C, Kwasny MJ, Cai X, Mohr DC. Therapeutic alliance in face-to-face and telephone-administered cognitive behavioral therapy. J Consult Clin Psychol. 2014;82(2):349-54. doi:10.1037/a0035554.

135. Stiles-Shields C, Corden ME, Kwasny MJ, Schueller SM, Mohr DC. Predictors of outcome for telephone and face-to-face administered cognitive behavioral therapy for depression. Psychol Med. 2015;45(15):3205-15. doi:10.1017/S0033291715001208.

136. Choi NG, Marti CN, Bruce ML, Hegel MT, Wilson NL, Kunik ME. Six-month postintervention depression and disability outcomes of in-home telehealth problem-solving therapy for depressed, low-income homebound older adults. Depress Anxiety. 2014;31(8):653-61. doi:10.1002/da.22242.

137. Choi NG, Marti CN, Conwell Y. Effect of problem-solving therapy on depressed low-income homebound older adults' death/suicidal ideation and hopelessness. Suicide Life Threat Behav. 2016;46(3):323-36. doi:10.1111/sltb.12195.

138. Egede LE, Acierno R, Knapp RG, Lejuez C, Hernandez-Tejada M, Payne EH, et al. Psychotherapy for depression in older veterans via telemedicine: A randomised, open-label, non-inferiority trial. Lancet Psychiatry. 2015;2(8):693-701. doi:10.1016/S2215-0366(15)00122-4.

139. Egede LE, Acierno R, Knapp RG, Walker RJ, Payne EH, Frueh BC. Psychotherapy for depression in older veterans via telemedicine: Effect on quality of life, satisfaction, treatment credibility, and service delivery perception. J Clin Psychiatry. 2016;77(12):1704-11. doi:10.4088/JCP.16m10951.

140. Egede LE, Gebregziabher M, Walker RJ, Payne EH, Acierno R, Frueh BC. Trajectory of cost overtime after psychotherapy for depression in older veterans via telemedicine. J Affect Disord. 2017;207:157-62. doi:10.1016/j.jad.2016.09.044.

141. Egede LE, Dismuke CE, Walker RJ, Acierno R, Frueh BC. Cost-effectiveness of behavioral activation for depression in older adult veterans: In-person care versus telehealth. J Clin Psychiatry. 2018a;79(5):28. doi:10.4088/JCP.17m11888.

142. Egede LE, Walker RJ, Payne EH, Knapp RG, Acierno R, Frueh BC. Effect of psychotherapy for depression via home telehealth on glycemic control in adults with type 2 diabetes: Subgroup analysis of a randomized clinical trial. J Telemed Telecare. 2018b;24(9):596-602. doi:10.1177/1357633X17730419.

143. Luxton DD, Pruitt LD, Wagner A, Smolenski DJ, Jenkins-Guarnieri MA, Gahm G. Home-based telebehavioral health for U.S. military personnel and veterans with depression: A randomized controlled trial. J Consult Clin Psychol. 2016;84(11):923-34. doi:10.1037/ccp0000135.

144. Smolenski DJ, Pruitt LD, Vuletic S, Luxton DD, Gahm G. Unobserved heterogeneity in response to treatment for depression through videoconference. Psychiatr Rehabil J. 2017;40(3):303-8. doi:10.1037/prj0000273.

145. Pruitt LD, Vuletic S, Smolenski DJ, Wagner A, Luxton DD, Gahm GA. Predicting post treatment client satisfaction between behavioural activation for depression delivered either in-person or via home-based telehealth. J Telemed Telecare. 2019;25(8):460-7. doi:10.1177/1357633X18784103.

146. Bouchard S, Allard M, Robillard G, Dumoulin S, Guitard T, Loranger C, et al. Videoconferencing psychotherapy for panic disorder and agoraphobia: Outcome and treatment processes from a non-randomized non-inferiority trial. Front Psychol. 2020;11:2164-. doi:10.3389/fpsyg.2020.02164.

147. Milosevic I, Cameron DH, Milanovic M, McCabe RE, Rowa K. Face-to-face versus video teleconference group cognitive behavioural therapy for anxiety and related disorders: A preliminary comparison: Therapie cognitivo-comportementale de groupe en personne contre par video teleconference pour l'anxiete et les troubles connexes: Une comparaison preliminaire. Can J Psychiatry. 2021;67(5):391-402. doi:10.1177/07067437211027319.

148. Watts S, Marchand A, Bouchard S, Gosselin P, Langlois F, Belleville G, et al. Telepsychotherapy for generalized anxiety disorder: Impact on the working alliance. J Psychother Integr. 2020;30(2):208-25. doi:10.1037/int0000223.

149. Acierno R, Gros DF, Ruggiero KJ, Hernandez-Tejada BM, Knapp RG, Lejuez CW, et al. Behavioral activation and therapeutic exposure for posttraumatic stress disorder: A noninferiority trial of treatment delivered in person versus home-based telehealth. Depress Anxiety. 2016;33(5):415-23. doi:10.1002/da.22476.

150. Acierno R, Knapp R, Tuerk P, Gilmore AK, Lejuez C, Ruggiero K, et al. A non-inferiority trial of Prolonged Exposure for posttraumatic stress disorder: In person versus home-based telehealth. Behav Res Ther. 2017;89:57-65. doi:10.1016/j.brat.2016.11.009.

151. Gros DF, Allan NP, Lancaster CL, Szafranski DD, Acierno R. Predictors of treatment discontinuation during prolonged exposure for PTSD. Behav Cogn Psychother. 2018;46(1):35-49. doi:10.1017/S135246581700039X.

152. Acierno R, Jaffe AE, Gilmore AK, Birks A, Denier C, Muzzy W, et al. A randomized clinical trial of in-person vs. home-based telemedicine delivery of Prolonged Exposure for PTSD in military sexual trauma survivors. J Anxiety Disord. 2021;83:102461. doi:10.1016/j.janxdis.2021.102461.

153. White CN, Kauffman BY, Acierno R. Factors contributing to veterans' satisfaction with PTSD treatment delivered in person compared to telehealth. J Telemed Telecare. 2021;http://dx.doi.org/10.1177/1357633X20987704. doi:10.1177/1357633X20987704.

154. Morland LA, Mackintosh MA, Glassman LH, Wells SY, Thorp SR, Rauch SAM, et al. Home-based delivery of variable length prolonged exposure therapy: A comparison of clinical efficacy between service modalities. Depress Anxiety. 2020;37(4):346-55. doi:10.1002/da.22979.

155. Morland LA, Mackintosh MA, Greene CJ, Rosen CS, Chard KM, Resick P, et al. Cognitive processing therapy for posttraumatic stress disorder delivered to rural veterans via telemental health: a randomized noninferiority clinical trial. J Clin Psychiatry. 2014;75(5):470-6. doi:10.4088/JCP.13m08842.

156. Morland LA, Mackintosh MA, Rosen CS, Willis E, Resick P, Chard K, et al. Telemedicine versus in-person delivery of cognitive processing therapy for women with posttraumatic stress disorder: A randomized noninferiority trial. Depress Anxiety. 2015;32(11):811-20. doi:10.1002/da.22397.

157. Glassman LH, Mackintosh MA, Talkovsky A, Wells SY, Walter KH, Wickramasinghe I, et al. Quality of life following treatment for PTSD: Comparison of videoconferencing and in-person modalities. J Telemed Telecare. 2019;25(2):123-7. doi:10.1177/1357633X17740610.

158. Liu L, Thorp SR, Moreno L, Wells SY, Glassman LH, Busch AC, et al. Videoconferencing psychotherapy for veterans with PTSD: Results from a randomized controlled non-inferiority trial. J Telemed Telecare. 2020;26(9):507-19. doi:10.1177/1357633X19853947.

159. Maieritsch KP, Smith TL, Hessinger JD, Ahearn EP, Eickhoff JC, Zhao Q. Randomized controlled equivalence trial comparing videoconference and in person delivery of cognitive processing therapy for PTSD. J Telemed Telecare. 2016;22(4):238-43. doi:10.1177/1357633X15596109.

160. Morland LA, Greene CJ, Rosen CS, Foy D, Reilly P, Shore J, et al. Telemedicine for anger management therapy in a rural population of combat veterans with posttraumatic stress disorder: A randomized noninferiority trial. J Clin Psychiatry. 2010;71(7):855-63. doi:10.4088/JCP.09m05604blu.

161. Morland LA, Greene CJ, Grubbs K, Kloezeman K, Mackintosh MA, Rosen C, et al. Therapist adherence to manualized cognitive-behavioral therapy for anger management delivered to veterans with PTSD via videoconferencing. J Clin Psychol. 2011;67(6):629-38. doi:10.1002/jclp.20779.

162. Greene CJ, Morland LA, Macdonald A, Frueh BC, Grubbs KM, Rosen CS. How does tele-mental health affect group therapy process? Secondary analysis of a noninferiority trial. J Consult Clin Psychol. 2010;78(5):746-50. doi:10.1037/a0020158.

163. Valentine LM, Donofry SD, Broman RB, Smith ER, Rauch SA, Sexton MB. Comparing PTSD treatment retention among survivors of military sexual trauma utilizing clinical video technology and in-person approaches. J Telemed Telecare. 2020;26(7-8):443-51. doi:10.1177/1357633X19832419.

164. Kelleher SA, Winger JG, Dorfman CS, Ingle KK, Moskovich AA, Abernethy AP, et al. A behavioral cancer pain intervention: A randomized noninferiority trial comparing in-person with videoconference delivery. Psychooncology. 2019;28(8):1671-8. doi:10.1002/pon.5141.

165. Check DK, Winger JG, Jones KA, Somers TJ. Predictors of response to an evidence-based behavioral cancer pain management intervention: An exploratory analysis from a clinical trial. J Pain Symptom Manage. 2021;62(2):391-9. doi:10.1016/j.jpainsymman.2020.12.020.

166. Herbert MS, Afari N, Liu L, Heppner P, Rutledge T, Williams K, et al. Telehealth versus in-person acceptance and commitment therapy for chronic pain: A randomized noninferiority trial. J Pain. 2017;18(2):200-11. doi:10.1016/j.jpain.2016.10.014.

167. Levy RL, Langer SL, van Tilburg MAL, Romano JM, Murphy TB, Walker LS, et al. Brief telephone-delivered cognitive behavioral therapy targeted to parents of children with functional abdominal pain: A randomized controlled trial. Pain. 2017;158(4):618-28. doi:10.1097/j.pain.0000000000000800.

168. Chavooshi B, Mohammadkhani P, Dolatshahee B. Telemedicine vs. in-person delivery of intensive short-term dynamic psychotherapy for patients with medically unexplained pain: A 12-month randomized, controlled trial. J Telemed Telecare. 2017;23(1):133-41. doi:10.1177/1357633X15627382.

169. Arnedt JT, Conroy DA, Mooney A, Furgal A, Sen A, Eisenberg D. Telemedicine versus face-to-face delivery of cognitive behavioral therapy for insomnia: A randomized controlled noninferiority trial. Sleep. 2021;44(1):21. doi:10.1093/sleep/zsaa136.

170. Crow SJ, Mitchell JE, Crosby RD, Swanson SA, Wonderlich S, Lancanster K. The cost effectiveness of cognitive behavioral therapy for bulimia nervosa delivered via telemedicine versus face-to-face. Behav Res Ther. 2009;47(6):451-3. doi:10.1016/j.brat.2009.02.006.

171. Mitchell JE, Crosby RD, Wonderlich SA, Crow S, Lancaster K, Simonich H, et al. A randomized trial comparing the efficacy of cognitive-behavioral therapy for bulimia nervosa delivered via telemedicine versus face-to-face. Behav Res Ther. 2008;46(5):581-92. doi:10.1016/j.brat.2008.02.004.

172. Marrone S, Mitchell JE, Crosby R, Wonderlich S, Jollie-Trottier T. Predictors of response to cognitive behavioral treatment for bulimia nervosa delivered via telemedicine versus face-to-face. Int J Eat Disord. 2009;42(3):222-7. doi:10.1002/eat.20603.

173. Ertelt TW, Crosby RD, Marino JM, Mitchell JE, Lancaster K, Crow SJ. Therapeutic factors affecting the cognitive behavioral treatment of bulimia nervosa via telemedicine versus face-to-face delivery. Int J Eat Disord. 2011;44(8):687-91. doi:10.1002/eat.20874.

174. Zerwas SC, Watson HJ, Hofmeier SM, Levine MD, Hamer RM, Crosby RD, et al. CBT4BN: A randomized controlled trial of online chat and face-to-face group therapy for bulimia nervosa. Psychother Psychosom. 2017;86(1):47-53. doi:10.1159/000449025.

175. Watson HJ, Levine MD, Zerwas SC, Hamer RM, Crosby RD, Sprecher CS, et al. Predictors of dropout in face-to-face and internet-based cognitive-behavioral therapy for bulimia nervosa in a randomized controlled trial. Int J Eat Disord. 2017a;50(5):569-77. doi:10.1002/eat.22644.

176. Watson HJ, McLagan N, Zerwas SC, Crosby RD, Levine MD, Runfola CD, et al. Cost-effectiveness of internet-based cognitive-behavioral treatment for bulimia nervosa: Results of a randomized controlled trial. J Clin Psychiatry. 2018;79(1). doi:10.4088/JCP.16m11314.

177. Lindegaard Pedersen J, Pedersen PU, Damsgaard EM. Nutritional follow-up after discharge prevents readmission to hospital - A randomized clinical trial. J Nutr Health Aging. 2017;21(1):75-82. doi:10.1007/s12603-016-0745-7.

178. Lovell K, Cox D, Haddock G, Jones C, Raines D, Garvey R, et al. Telephone administered cognitive behaviour therapy for treatment of obsessive compulsive disorder: Randomised controlled non-inferiority trial. BMJ. 2006;333(7574):883. doi:10.1136/bmj.38940.355602.80.

179. Turner CM, Mataix-Cols D, Lovell K, Krebs G, Lang K, Byford S, et al. Telephone cognitive-behavioral therapy for adolescents with obsessive-compulsive disorder: A randomized controlled non-inferiority trial. J Am Acad Child Adolesc Psychiatry. 2014;53(12):1298-307.e2. doi:10.1016/j.jaac.2014.09.012.

180. Nair A, Turner C, Heyman I, Mataix-Cols D, Lovell K, Krebs G, et al. Moderators and predictors of outcomes in telephone delivered compared to face-to-face cognitive behaviour therapy for paediatric obsessive-compulsive disorder: Preliminary evidence from a non-inferiority RCT. Cogn Behav Ther. 2019;48(5):353-68. doi:10.1080/16506073.2018.1513555.

181. Tie H, Krebs G, Lang K, Shearer J, Turner C, Mataix-Cols D, et al. Cost-effectiveness analysis of telephone cognitive-behaviour therapy for adolescents with obsessive-compulsive disorder. BJPsych Open. 2019;5(1):e7. doi:10.1192/bjo.2018.73.

182. Watson M, White C, Lynch A, Mohammed K. Telephone-delivered individual cognitive behavioural therapy for cancer patients: An equivalence randomised trial. Psychooncology. 2017b;26(3):301-8. doi:10.1002/pon.4338.

183. Rodrigues P, Watson M, White C, Lynch A, Mohammed K, Sagoo GS. Cost-effectiveness analysis of telephone-based cognitive behaviour therapy compared to treatment as usual CBT for cancer patients: Evidence from a small, randomised controlled trial. Psychooncology. 2021;30(10):1691-8. doi:10.1002/pon.5751.

184. Lleras de Frutos M, Medina JC, Vives J, Casellas-Grau A, Marzo JL, Borras JM, et al. Video conference vs face-to-face group psychotherapy for distressed cancer survivors: A randomized controlled trial. Psychooncology. 2020;29(12):1995-2003. doi:10.1002/pon.5457.

185. Guzman D, Ann-Yi S, Bruera E, Wu J, Williams JL, Najera J, et al. Enhancing palliative care patient access to psychological counseling through outreach telehealth services. Psychooncology. 2020;29(1):132-8. doi:10.1002/pon.5270.

186. Morgan RD, Patrick AR, Magaletta PR. Does the use of telemental health alter the treatment experience? Inmates' perceptions of telemental health versus face-to-face treatment modalities. J Consult Clin Psychol. 2008;76(1):158-62. doi:10.1037/0022-006x.76.1.158.

187. Ospina-Pinillos L, Davenport T, Iorfino F, Tickell A, Cross S, Scott EM, et al. Using new and innovative technologies to assess clinical stage in early intervention youth mental health services: Evaluation study. J Med Internet Res. 2018;20(9):e259. doi:10.2196/jmir.9966.

188. Fukkink RG, Hermanns JM. Children's experiences with chat support and telephone support. J Child Psychol Psychiatry. 2009a;50(6):759-66. doi:10.1111/j.1469-7610.2008.02024.x.

189. Fukkink R, Hermanns J. Counseling children at a helpline: Chatting or calling? J Community Psychol. 2009b;37(8):939-48. doi:10.1002/jcop.20340.

190. King R, Bambling M, Reid W, Thomas I. Telephone and online counselling for young people: A naturalistic comparison of session outcome, session impact and therapeutic alliance. Couns Psychother Res. 2006;6(3):175-81. doi:10.1080/14733140600874084.

191. Murphy L, Parnass P, Mitchell DL, Hallett R, Cayley P, Seagram S. Client satisfaction and outcome comparisons of online and face-to-face counselling methods. Br J Soc Work. 2009;39(4):627-40. doi:10.1093/bjsw/bcp041.

192. Laver K, Liu E, Clemson L, Davies O, Gray L, Gitlin LN, et al. Does telehealth delivery of a dyadic dementia care program provide a noninferior alternative to face-to-face delivery of the same program? A randomized, controlled trial. Am J Geriatr Psychiatry. 2020;28(6):673-82. doi:10.1016/j.jagp.2020.02.009.

193. Wade SL, Cassedy AE, McNally KA, Kurowski BG, Kirkwood MW, Stancin T, et al. A randomized comparative effectiveness trial of family-problem-solving treatment for adolescent brain injury: Parent outcomes from the Coping with Head Injury Through Problem Solving (Chips) Study. J Head Trauma Rehabil. 2019;34(6):E1-E9. doi:10.1097/HTR.0000000000000487.

194. Corona LL, Stainbrook JA, Simcoe K, Wagner L, Fowler B, Weitlauf AS, et al. Utilization of telemedicine to support caregivers of young children with ASD and their Part C service providers: A comparison of intervention outcomes across three models of service delivery. J Neurodev Disord. 2021;13(38):13 pages. doi:10.1186/s11689-021-09387-w.

195. Kalichman SC, Katner H, Eaton LA, Banas E, Hill M, Kalichman MO. Comparative effects of telephone versus in-office behavioral counseling to improve HIV treatment outcomes among people living with HIV in a rural setting. Transl Behav Med. 2021a;11(3):852-62. doi:10.1093/tbm/ibaa109.

196. Kalichman SC, Katner H, Eaton LA, Hill M, Ewing W, Kalichman MO. Randomized community trial comparing telephone versus clinic-based behavioral health counseling for people living with HIV in a rural setting. J Rural Health. 2021b;http://dx.doi.org/10.1111/jrh.12618. doi:10.1111/jrh.12618.

197. Phanuphak N, Anand T, Jantarapakde J, Nitpolprasert C, Himmad K, Sungsing T, et al. What would you choose: Online or offline or mixed services? Feasibility of online HIV counselling and testing among Thai men who have sex with men and transgender women and factors associated with service uptake. J Int AIDS Soc. 2018;21 Suppl 5:e25118. doi:10.1002/jia2.25118.

198. Delahanty LM, Chang Y, Levy DE, Porneala B, Dushkin A, Bissett L, et al. Design and participant characteristics of a primary care adaptation of the Look AHEAD Lifestyle Intervention for weight loss in type 2 diabetes: The REAL HEALTH-diabetes study. Contemp Clin Trials. 2018;71:9-17. doi:10.1016/j.cct.2018.05.018.

199. Delahanty LM, Levy DE, Chang Y, Porneala BC, Goldman V, McCarthy J, et al. Effectiveness of lifestyle intervention for type 2 diabetes in primary care: the REAL HEALTH-Diabetes randomized clinical trial. J Gen Intern Med. 2020;35(9):2637-46. doi:10.1007/s11606-019-05629-9.

200. Duke DC, Wagner DV, Ulrich J, Freeman KA, Harris MA. Videoconferencing for teens with diabetes: Family matters. J Diabetes Sci Technol. 2016;10(4):816-23. doi:10.1177/1932296816642577.

201. Harris MA, Freeman KA, Duke DC. Seeing is believing: Using skype to improve diabetes outcomes in youth. Diabetes Care. 2015;38(8):1427-34. doi:10.2337/dc14-2469.

202. Riley AR, Duke DC, Freeman KA, Hood KK, Harris MA. Depressive symptoms in a trial behavioral family systems therapy for diabetes: A post hoc analysis of change. Diabetes Care. 2015;38(8):1435-40. doi:10.2337/dc14-2519.

203. Nevanpera N, Keranen AM, Ukkola O, Laitinen J. Effects of group counseling transmitted through videoconferencing on changes in eating behaviors. J Nutr Educ Behav. 2015;47(6):555-9.e1. doi:10.1016/j.jneb.2015.07.004.

204. Harder VS, Musau AM, Musyimi CW, Ndetei DM, Mutiso VN. A randomized clinical trial of mobile phone motivational interviewing for alcohol use problems in Kenya. Addiction. 2020;115(6):1050-60. doi:10.1111/add.14903.

205. Lohr PA, Aiken ARA, Forsyth T, Trussell J. Telephone or integrated contraception counselling before abortion: Impact on method choice and receipt. BMJ Sex Reprod Health. 2018;44(2):114-21. doi:10.1136/bmjsrh-2017-101818.

206. James EL, Ewald BD, Johnson NA, Stacey FG, Brown WJ, Holliday EG, et al. Referral for expert physical activity counseling: A pragmatic RCT. Am J Prev Med. 2017;53(4):490-9. doi:10.1016/j.amepre.2017.06.016.

207. Andrews M, Baker AL, Halpin SA, Lewin TJ, Richmond R, Kay-Lambkin FJ, et al. Early therapeutic alliance, treatment retention, and 12-month outcomes in a healthy lifestyles intervention for people with psychotic disorders. J Nerv Ment Dis. 2016;204(12):894-902. doi:10.1097/NMD.0000000000000585.

208. Baker AL, Richmond R, Kay-Lambkin FJ, Filia SL, Castle D, Williams JM, et al. Randomized controlled trial of a healthy lifestyle intervention among smokers with psychotic disorders. Nicotine Tob Res. 2015;17(8):946-54. doi:10.1093/ntr/ntv039.

209. Baker AL, Richmond R, Kay-Lambkin FJ, Filia SL, Castle D, Williams JM, et al. Randomised controlled trial of a healthy lifestyle intervention among smokers with psychotic disorders: Outcomes to 36 months. Aust N Z J Psychiatry. 2018;52(3):239-52. doi:10.1177/0004867417714336.

210. Berndt N, Bolman C, Lechner L, Max W, Mudde A, de Vries H, et al. Economic evaluation of a telephone- and face-to-face-delivered counseling intervention for smoking cessation in patients with coronary heart disease. Eur J Health Econ. 2016;17(3):269-85. doi:10.1007/s10198-015-0677-x.

211. Byaruhanga J, Paul CL, Wiggers J, Byrnes E, Mitchell A, Lecathelinais C, et al. Connectivity of real-time video counselling versus telephone counselling for smoking cessation in rural and remote areas: An exploratory study. Int J Environ Res Public Health. 2020a;17(8):22. doi:10.3390/ijerph17082891.

212. Byaruhanga J, Wiggers J, Paul CL, Byrnes E, Mitchell A, Lecathelinais C, et al. Acceptability of real-time video counselling compared to other behavioural interventions for smoking cessation in rural and remote areas. Drug Alcohol Depend. 2020b;217:108296. doi:10.1016/j.drugalcdep.2020.108296.

213. Byaruhanga J, Paul CL, Wiggers J, Byrnes E, Mitchell A, Lecathelinais C, et al. The short-term effectiveness of real-time video counselling on smoking cessation among residents in rural and remote areas: An interim analysis of a randomised trial. J Subst Abuse Treat. 2021;131:108448. doi:10.1016/j.jsat.2021.108448.

214. Carlson LE, Lounsberry JJ, Maciejewski O, Wright K, Collacutt V, Taenzer P. Telehealth-delivered group smoking cessation for rural and urban participants: Feasibility and cessation rates. Addict Behav. 2012;37(1):108-14. doi:10.1016/j.addbeh.2011.09.011.

215. Nomura A, Tanigawa T, Muto T, Oga T, Fukushima Y, Kiyosue A, et al. Clinical efficacy of telemedicine compared to face-to-face clinic visits for smoking cessation: Multicenter open-label randomized controlled noninferiority trial. J Med Internet Res. 2019;21(4):e13520-e. doi:10.2196/13520.

216. Richter KP, Shireman TI, Ellerbeck EF, Cupertino AP, Catley D, Cox LS, et al. Comparative and cost effectiveness of telemedicine versus telephone counseling for smoking cessation. J Med Internet Res. 2015;17(5):e113. doi:10.2196/jmir.3975.

217. Appel LJ, Clark JM, Yeh H-C, Wang N-Y, Coughlin JW, Daumit G, et al. Comparative effectiveness of weight-loss interventions in clinical practice. N Engl J Med. 2011;365(21):1959-68. doi:10.1056/NEJMoa1108660.

218. Dalcin AT, Jerome GJ, Fitzpatrick SL, Louis TA, Wang NY, Bennett WL, et al. Perceived helpfulness of the individual components of a behavioural weight loss program: Results from the Hopkins POWER Trial. Obes Sci Pract. 2015;1(1):23-32. doi:10.1002/osp4.6.

219. Daumit GL, Janssen EM, Jerome GJ, Dalcin AT, Charleston J, Clark JM, et al. Cost of behavioral weight loss programs implemented in clinical practice: The POWER trial at Johns Hopkins. Transl Behav Med. 2020;10(1):103-13. doi:10.1093/tbm/iby120.

220. Befort CA, VanWormer JJ, Desouza C, Ellerbeck EF, Gajewski B, Kimminau KS, et al. Effect of behavioral therapy with in-clinic or telephone group visits vs in-clinic individual visits on weight loss among patients with obesity in rural clinical practice: A randomized clinical trial. JAMA. 2021;325(4):363-72. doi:10.1001/jama.2020.25855.

221. Kurz D, McCrea-Robertson S, Nelson-Brantley H, Befort C. Rural engagement in primary care for optimizing weight reduction (REPOWER): A mixed methods study of patient perceptions. Patient Educ Couns. 2021;10.1016/j.pec.2021.11.028. doi:10.1016/j.pec.2021.11.028.

222. Fujii H, Yokoyama T, Yoshimi I, Mizushima S. A randomized controlled trial to evaluate the effects of health guidance with video call as compared to face-to-face health guidance. Int Medical J. 2017;24(2):186-91.

223. Harrigan M, Cartmel B, Loftfield E, Sanft T, Chagpar AB, Zhou Y, et al. Randomized trial comparing telephone versus in-person weight loss counseling on body composition and circulating biomarkers in women treated for breast cancer: The lifestyle, exercise, and nutrition (LEAN) study. J Clin Oncol. 2016;34(7):669-76. doi:10.1200/JCO.2015.61.6375.

224. Harvey-Berino J. Changing health behavior via telecommunications technology: Using interactive television to treat obesity. Behav Ther. 1998;29(3):505-19. doi:10.1016/S0005-7894(98)80046-4.

225. Lukenbill T, Giovanni CS, Simpson A, Chew M, Basco W, Roberts J. Assessing anthropometric and laboratory outcomes of a paediatric telehealth weight management program. J Telemed Telecare. 2021;http://dx.doi.org/10.1177/1357633X20986022. doi:10.1177/1357633X20986022.

226. Lutes LD, Dinatale E, Goodrich DE, Ronis DL, Gillon L, Kirsh S, et al. A randomized trial of a small changes approach for weight loss in veterans: Design, rationale, and baseline characteristics of the ASPIRE-VA trial. Contemp Clin Trials. 2013;34(1):161-72. doi:10.1016/j.cct.2012.09.007.

227. Damschroder LJ, Lutes LD, Kirsh S, Kim HM, Gillon L, Holleman RG, et al. Small-changes obesity treatment among veterans: 12-month outcomes. Am J Prev Med. 2014;47(5):541-53. doi:10.1016/j.amepre.2014.06.016.

228. Vimalananda V, Damschroder L, Janney CA, Goodrich D, Kim HM, Holleman R, et al. Weight loss among women and men in the ASPIRE-VA behavioral weight loss intervention trial. Obesity. 2016;24(9):1884-91. doi:10.1002/oby.21574.

229. Lutes LD, Damschroder LJ, Masheb R, Kim HM, Gillon L, Holleman RG, et al. Behavioral treatment for veterans with obesity: 24-month weight outcomes from the aspire-va small changes randomized trial. J Gen Intern Med. 2017;32(Suppl 1):40-7. doi:10.1007/s11606-017-3987-0.

230. Fernandez E, Woldgabreal Y, Day A, Pham T, Gleich B, Aboujaoude E. Live psychotherapy by video versus in-person: A meta-analysis of efficacy and its relationship to types and targets of treatment. Clin Psychol Psychother. 2021;07. doi:10.1002/cpp.2594.

231. Thomas N, McDonald C, de Boer K, Brand RM, Nedeljkovic M, Seabrook L. Review of the current empirical literature on using videoconferencing to deliver individual psychotherapies to adults with mental health problems. Psychol Psychother. 2021;94(3):854-83. doi:10.1111/papt.12332.

232. Byaruhanga J, Atorkey P, McLaughlin M, Brown A, Byrnes E, Paul C, et al. Effectiveness of individual real-time video counseling on smoking, nutrition, alcohol, physical activity, and obesity health risks: Systematic review. J Med Internet Res. 2020;22(9):e18621. doi:10.2196/18621.

233. Irvine A, Drew P, Bower P, Brooks H, Gellatly J, Armitage CJ, et al. Are there interactional differences between telephone and face-to-face psychological therapy? A systematic review of comparative studies. J Affect Disord. 2020;265:120-31. doi:10.1016/j.jad.2020.01.057.

234. Ho C, Severn M. e-Therapy interventions for the treatment of patients with depression: A review of clinical effectiveness. CADTH rapid response report: Summary with critical appraisal. Ottawa: Canadian Agency for Drugs and Technologies in Health; 2018 May 18 [cited 2022 Mar 18]. Available from: https://cadth.ca/e-therapy-interventions-treatments-patients-depression-review-clinical-effectiveness.

235. Turgoose D, Ashwick R, Murphy D. Systematic review of lessons learned from delivering tele-therapy to veterans with post-traumatic stress disorder. J Telemed Telecare. 2018;24(9):575-85. doi:10.1177/1357633X17730443.

236. Jobes DA, Crumlish JA, Evans AD. The COVID-19 pandemic and treating suicidal risk: The telepsychotherapy use of CAMS. J Psychother Integr. 2020;30(2):226-37. doi:10.1037/int0000208.

237. Rosen CS, Glassman LH, Morland LA. Telepsychotherapy during a pandemic: A traumatic stress perspective. J Psychother Integr. 2020;30(2):174-87. doi:10.1037/int0000221.
